# tRNA-Cys gene clusters exhibit high variability in *Arabidopsis thaliana*

**DOI:** 10.1186/s12870-023-04632-x

**Published:** 2023-12-07

**Authors:** Maciej Szymanski, Anand Maurya, Piotr Kopec, Wojciech M. Karlowski

**Affiliations:** Department of Computational Biology, Adam Mickiewicz University in Poznan, Uniwersytetu Poznanskiego 6, 61-614 Poznan, Poland

**Keywords:** tRNA, tRNA gene clusters, Transposable elements, Helitron

## Abstract

Although most of the genes encoding tRNAs in plants are dispersed throughout the genome, a fraction of them form tRNA gene clusters. In *Arabidopsis thaliana*, the smallest of tRNA clusters on chromosome 5 consists of four tRNA-Cys-GCA genes placed within repeating units of 0.4 kbp. A systematic analysis of the genomic sequences of syntenic regions from various ecotypes of *A. thaliana* showed that the general structure of the cluster, consisting of a tRNA-Cys pseudogene followed by repeating units containing tRNA-Cys genes, is well conserved. However, there is significant heterogeneity in the number of repeating units between different ecotypes. A unique feature of this cluster is the presence of putative transposable elements (Helitron). In addition, two further tRNA-Cys gene mini-clusters (gene pairs) in *A. thaliana* were identified. RNA-seq-based evaluation of expression of tRNA-Cys-GCA genes showed a positive signal for 11 out of 13 unique transcripts. An analysis of the conservation of the tRNA-Cys clusters from *A. thaliana* with the corresponding regions from four other *Arabidopsis* species suggests a sequence of events that led to the divergence of these regions.

**Supplementary Information:**

The online version contains supplementary material available at 10.1186/s12870-023-04632-x.

## Introduction

Transfer RNAs (tRNAs) play a vital role in all organisms. In the process of protein biosynthesis, aminoacylated tRNA molecules serve as an interface between the genetic information contained in the protein-coding genes and its expression in the form of functional proteins. Thus, the efficient and error-free production of proteins in the cell depends on the expression of the full set of tRNAs capable of decoding all the codons used in messenger RNAs. In addition, it has been recently shown [1] that the redundancy in tRNA pools shows a fitness effect depending on nutrient availability.

Although the tRNAs from all domains of life share all the essential features of a common cloverleaf secondary structure, the mechanisms of their expression and gene organization differ between prokaryotes (and organelles) and eukaryotes. In Bacteria, Archaea, and organelles tRNAs can be expressed either as monocistronic transcripts from individual genes or as parts of polycistronic transcripts from tRNA and ribosomal RNA operons. A common feature of the prokaryotic tRNA genes transcription is their dependence on typical bacterial promoters consisting of -35 and − 10 elements [2].

With a few exceptions, in eukaryotes, each tRNA is transcribed from an independent gene by a specialized RNA polymerase III (Pol III). Transcription of eukaryotic tRNA genes depends on the internal promoter elements, boxes A and B recognized by transcription factor IIIC. The positions of boxes A and B within the tRNA gene correspond to tRNA structure elements: D- and T-stem-loop, respectively [3].

The majority of tRNA genes in higher eukaryotes are more or less evenly dispersed throughout the genome. In some instances, as a consequence of gene duplication, tRNA gene clusters can be formed. The tRNA gene clusters in eukaryotes are defined as genomic regions containing only tRNA genes. Sometimes in defining such clusters an additional criterion of the maximum size of the intergenic regions between tRNA genes (usually 1 kbp) is considered. In contrast to Bacteria, in which the clusters of co-expressed tRNA genes are usually composed of genes encoding tRNAs with different amino acid specificities, the eukaryotic tRNA gene clusters are often composed of closely related tRNA specific for one or two amino acids [4].

In the *Arabidopsis* genome, three clusters of tRNA-Pro genes, one cluster of tRNA-Ser and tRNA-Tyr, and one cluster of tRNA-Cys were identified [5, 6]. In the case of tRNA-Pro clusters, the genes and pseudogenes often overlap with the intronic regions of the protein-coding and ncRNA genes on the opposite strand. Conversely, in the largest cluster, spanning almost 40 kbp of chromosome 1 and consisting of 27 tandemly repeating units composed of one tRNA-Ser and two tRNA-Tyr genes, there are no other annotated genes. The smallest of the tRNA gene clusters described so far in *A. thaliana* reference genome consists of four tRNA-Cys-GCA genes and overlaps with three annotated Helitron superfamily transposable elements.

In this study, we present a detailed analysis of the variability of the tRNA-Cys gene cluster based on the genomic sequences of various *A. thaliana* accessions. We analyzed syntenic regions of chromosome 5 from 16 genomes derived from different ecotypes. The genomic sequences from these ecotypes show a high variation in the copy number of the repeating units and the nucleotide sequences of the tRNA genes. Moreover, the survey of the genomic sequences revealed two additional mini-clusters of tRNA-Cys genes on chromosomes 1 and 2, that in *A. thaliana* consist of two genes in head-to-tail or head-to-head orientation.

In this paper, we also show that although the entire chromosome 5 tRNA-Cys cluster is apparently epigenetically silenced [6], the transcriptional inactivation is not complete. An analysis of the tRNA expression in the reference Col-0 strain demonstrated that one of the tRNA genes located at the cluster’s boundary shows a low-level expression.

## Results

### The Cys-tRNA cluster on chromosome 5 is preceded by a pseudogene and contains marks of transposable elements

**Fig. 1:**
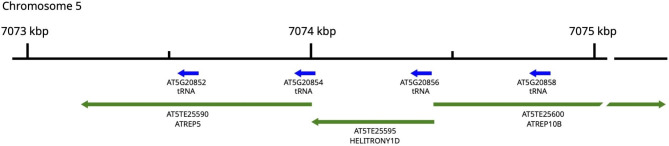
Localization of the tRNA-Cys gene cluster on chromosome 5 in the reference genome of A. thaliana (TAIR10). The tRNA genes and transposable elements annotated within this region are shown with blue and green arrows, respectively

In the reference sequence of the *A. thaliana* genome (TAIR10) [7] the Cys-tRNA cluster is located on chromosome 5 and consists of four tandemly arranged, ~ 420 bp long, repeating units, each of which contains a tRNA gene (Fig. 1). The pairwise identity of particular repeats of the cluster ranges from 84 to 94%. All four tRNA genes annotated in the TAIR10 genome pass the tRNAScan-SE [8] high-confidence filter which suggests that they may encode fully functional tRNA molecules. The nucleotide sequences of three of the tRNA genes are unique for the chromosome 5 cluster. For one of the genes (tRNA-Cys-GCA-9; AT5G20854), there is another copy encoding identical mature tRNA on chromosome 4. Moreover, in the region flanking the first repeating unit (AT5G20858), we have identified a sequence that is derived from the tRNA-Cys. This sequence was not identified in the tRNAScan-SE predictions and can be regarded as a tRNA-Cys pseudogene (see later in the text).

A unique feature of the tRNA-Cys cluster, when compared with other tRNA gene clusters, is the presence of transposable elements (TEs) in this region (Fig. 1). In the annotation of the reference genome, the entire cluster overlaps with three transposable elements belonging to the Helitron superfamily: ATREP5 (AT5TE25590), HELITRONY1D (AT5TE25595) and ATREP10B (AT5TE25600). The HELITRONY1D and ATREP5 have the same polarity as the tRNA genes and they have lengths corresponding to one or two tRNA-Cys gene cluster repeating units, respectively. Considering a high degree of identity between the repeats, the cluster could also be regarded as being composed of tandem repeats of these TEs. However, the 5’-terminal fragment of ATREP10B overlaps the first two repeats with the reversed polarity with respect to the tRNA genes.

Since the TAIR10 annotation of this region is based on the sequence similarity to the database of repetitive elements [9], we further explored the properties of this genomic fragment using bioinformatic tools designed for the identification of Helitron elements: HelitronFinder [10], HelSearch [11], HelitronScanner [12] and EAHelitron [13]. The first tool (HelitronFinder) was not available for download at the time of the study. Analysis of both strands of the genomic fragment containing the tRNA-Cys cluster (with 500 bp extensions on both sides), as well as the entire chromosome 5 with the remaining three algorithms, did not reveal any reliable prediction of Helitron transposable element that would overlap the tRNA genes located within the cluster.

### The structure of the chromosome 5 tRNA-Cys gene cluster shows high variability in *A. thaliana* accessions

**Fig. 2:**
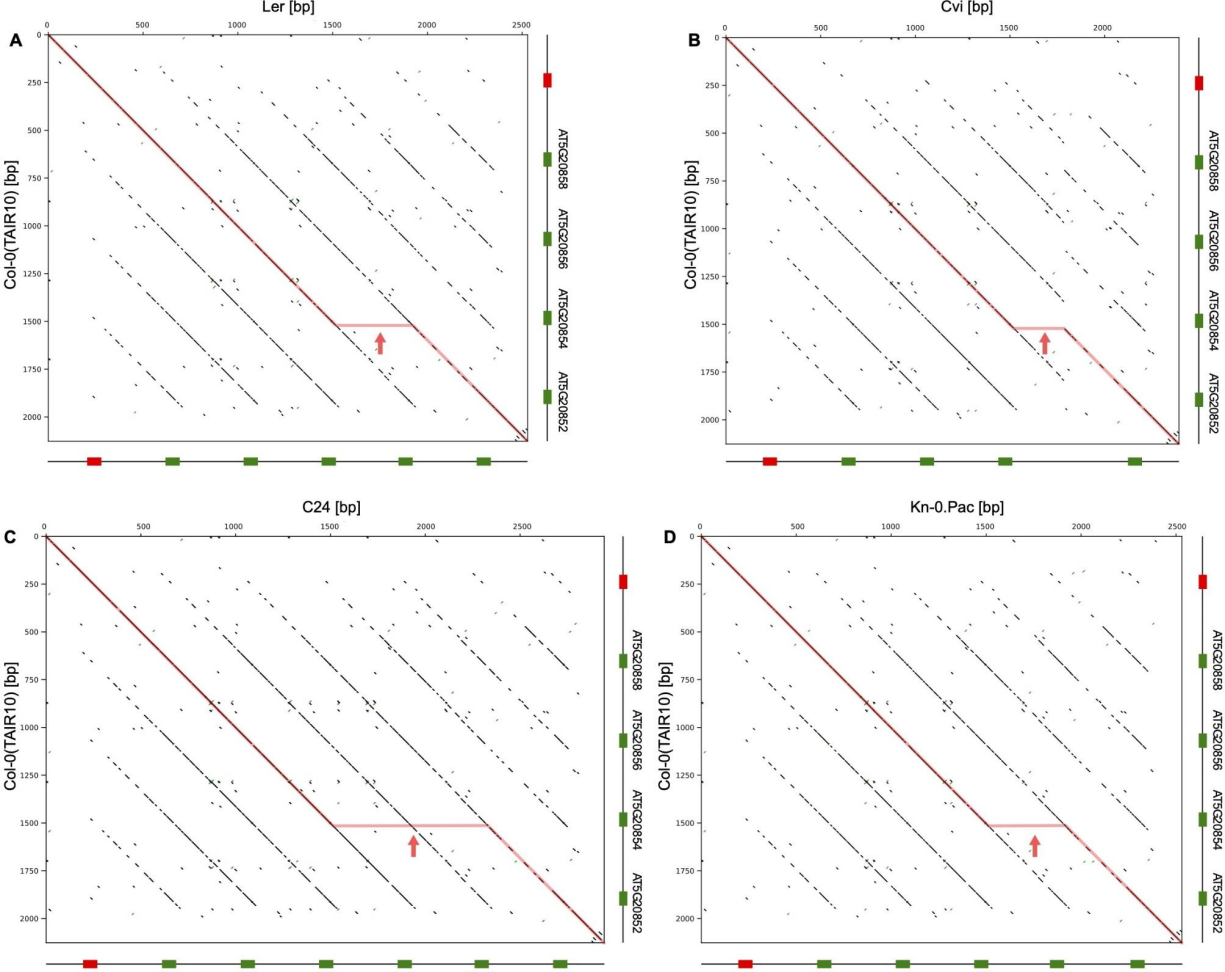
Dot-plot analysis of the heterogeneity of the chromosome 5 tRNA-Cys clusters for selected A. thaliana accessions. The dot-plots represent alignments of the chromosome 5 region from the reference genome (Col-0, TAIR10) with syntenic regions from genomic sequences of (**A**) Ler (**B**) Cvi (**C**) C24 and (**D**) Kn-0 (PacBio sequence). The tRNA-Cys genes and the pseudogenes are shown as green and red boxes, respectively. The red arrows indicate the inserted sequences observed in the tested Arabidopsis thaliana accessions

**Fig. 3:**
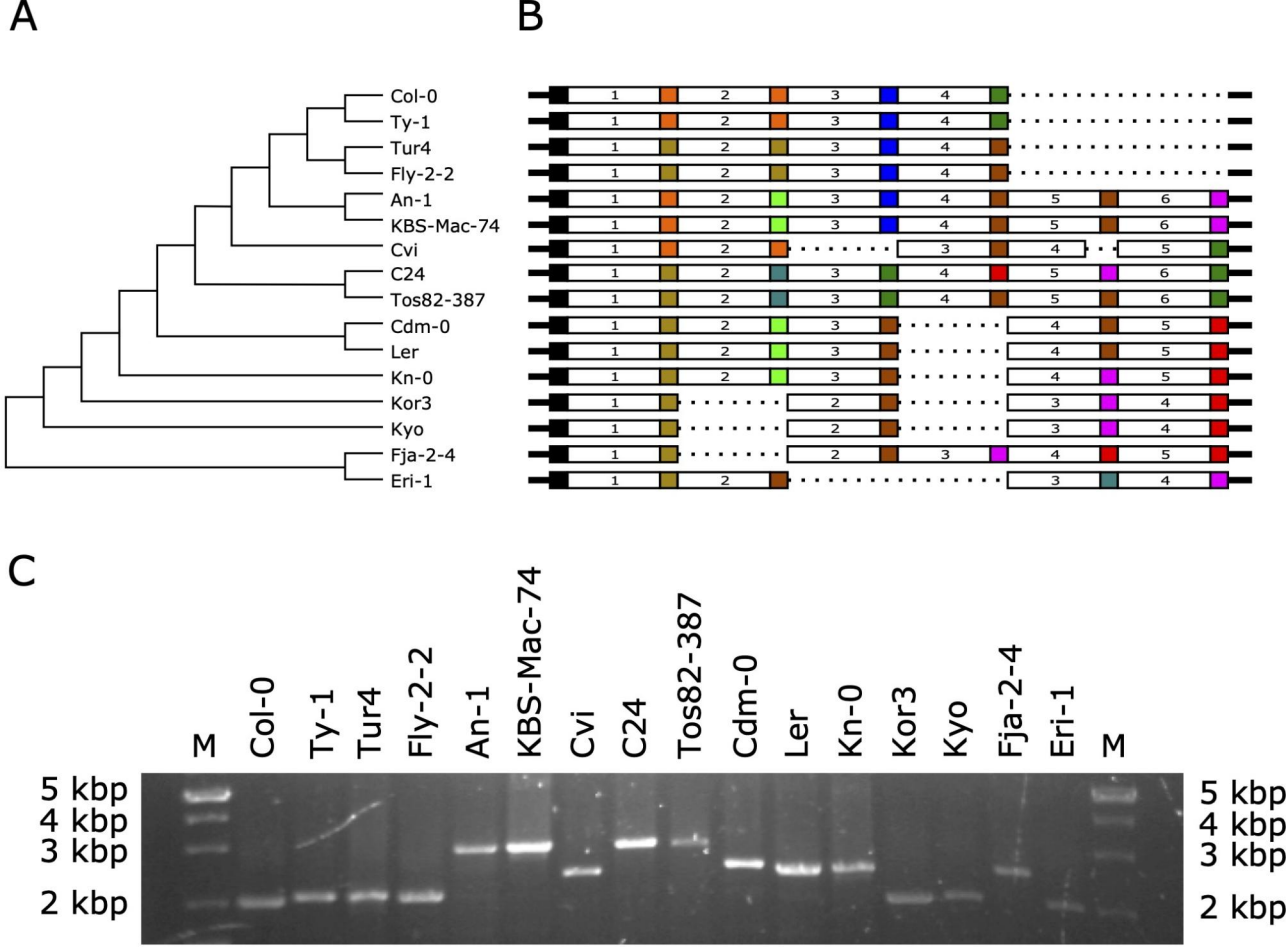
**A**) The evolutionary relationship between the chromosome 5 tRNA-Cys cluster sequences from the analyzed genomes and **B**) the structures of the clusters. Different colors are used for tRNAs belonging to distinct groups defined based on 97% sequence identity. **C**) PCR-based analysis of the size heterogeneity of the clusters. The fragments’ lengths confirm variations in the size of the cluster in tested A. thaliana genomes (see also Supplementary Figure S7)

Comparative analysis of the tRNA-Cys cluster from the reference genome with the corresponding regions from the genomic sequences of other *A. thaliana* accessions using dot-plots revealed a significant size heterogeneity of this region due to a variable number of repeats (Fig. 2). Detailed analysis of these regions showed the number of repeating units in the genomic sequences from different *A. thaliana* accessions vary from four to six. In the majority of genomes, there are four repeats, as in the case of the reference Col-0 genome. Sequence comparison of the clusters and flanking regions suggested that the first repeating units and the sequences downstream of the last tRNA gene are homologous. Thus, the heterogeneity results from the variation in the number of copies of repeats between these regions. In most cases, the heterogeneity in the length of the cluster is a consequence of the insertion or deletion of the entire repeating units including tRNA genes and spacer regions (Fig. 3). The only exception is the cluster from the Cvi accession in which there is a deletion of a 167 bp region that includes 95 base pairs of the repeat 3 and the tRNA gene of the repeat 4. The detailed location and multiple sequence alignment of all chromosome 5 tRNACys cluster regions are provided in the supplementary materials (Supplementary Table T2 and Figure S3).

Overall, pairwise identities between the tRNA-Cys clusters in all the analyzed genomes are in the range of ~ 80 to 100%. However, due to relatively high sequence similarity between repeating units, it is very difficult to establish proper alignment of the entire genomic fragments that would unambiguously reflect the relationship between particular regions in different genomic sequences. Using the phylogenetic analysis of the individual repeats, we were able to group the corresponding repeat units in all analyzed genomic sequences. The maps of the relative arrangement of these units presented in Fig. 3 were constructed to account for the sequence similarities between corresponding repeats from different genomes. The optimal phylogenetic tree of all the individual repeat unit sequences is presented in Supplementary Figure S1. The tree shows six branches that correspond to six repeating units of the longest clusters and the relationships between distinct groups of the repeats from different genomes.

To confirm the size heterogeneity of the chromosome 5 tRNA-Cys cluster observed in the genomic sequences we performed PCR with unique primers matching the conserved sequences flanking the genes. The results of the PCR reaction confirmed observed variations in the length of the cluster in tested *A. thaliana* accessions (Fig. 3). The size of the PCR products correlated with the existence of cluster variants consisting of four, five, and six repeating units.

### The tRNA-Cys genes exhibit high sequence variability within the cluster and between *A. thaliana* ecotypes

All of the tRNA genes in the tRNA-Cys gene cluster are derived from the same variant of the tRNA-Cys-GCA. There is, however, a notable sequence variation both between genes within one genome (90–100% identity) as well as between ecotypes (88–100% identity). Almost all the tRNA genes in all the analyzed regions were classified as ‘high confidence’ by the EukHighConfidenceFilter (Fig. 3B colored boxes). The ‘high confidence set’ consists of sequences best matching the canonical model of eukaryotic tRNAs. The sequences rejected by the filter do not possess all features of the secondary or tertiary structure of the ideal tRNA cloverleaf or show other unusual features like anticodon-tRNA isotype mismatch [8]. There are 34 sequence variants of tRNA regions associated with the repeats. However, a significant number of the repeats show substitutions that can affect the formation of the proper tRNA secondary structure by introducing mispairings in double-stranded regions including the acceptor stem, T-stem, and anticodon stem. The clustering of individual tRNA gene sequences is shown in Supplementary Figure S2.

A common feature of the chromosome 5 tRNACys gene clusters is the presence of the tRNACys pseudogene preceding the first repeat of the cluster. The pseudogene in this region was predicted by tRNAScan-SE only in the genomic sequence of the KBS-Mac-74. However, in all genomes, in the corresponding region, there are almost identical sequences present that can also be regarded as pseudogenes derived from the tRNA-Cys-GCA gene. Thus, in all the analyzed *A. thaliana* accessions, the cluster is flanked on one side by the well-conserved pseudogene showing, in pairwise comparison between any two sequences, 94 to 100% identity.

### Two novel mini clusters of the tRNA-Cys genes are located on chromosomes 1 and 2

**Fig. 4:**
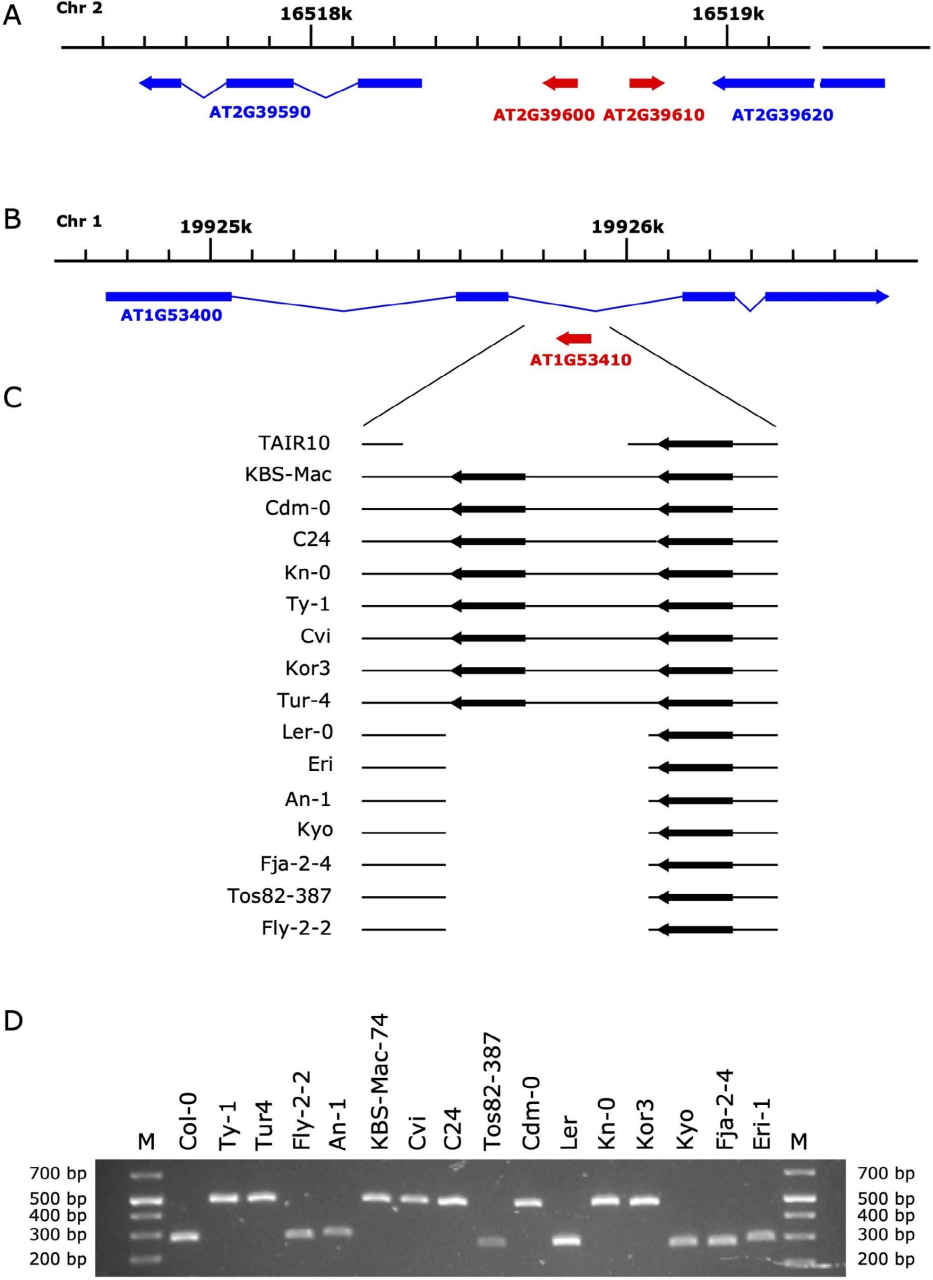
Mini clusters of tRNA-Cys genes. (**A**) A cluster of divergently transcribed tRNA-Cys genes on chromosome 2 conserved in all A. thaliana accessions analyzed in this study. (**B**) A cluster of tandemly repeated tRNA-Cys genes overlapping in antisense orientation the second intron of the gene encoding ubiquitin domain-containing protein on chromosome 1. (**C**) Schematic alignment of the chromosome 1 tRNA-Cys cluster region from various A. thaliana accessions. (**D**) Experimental evaluation of the observed clusters’ size variation using PCR amplification on genomic DNA (see also Supplementary Figure S8)

**Fig. 5:**
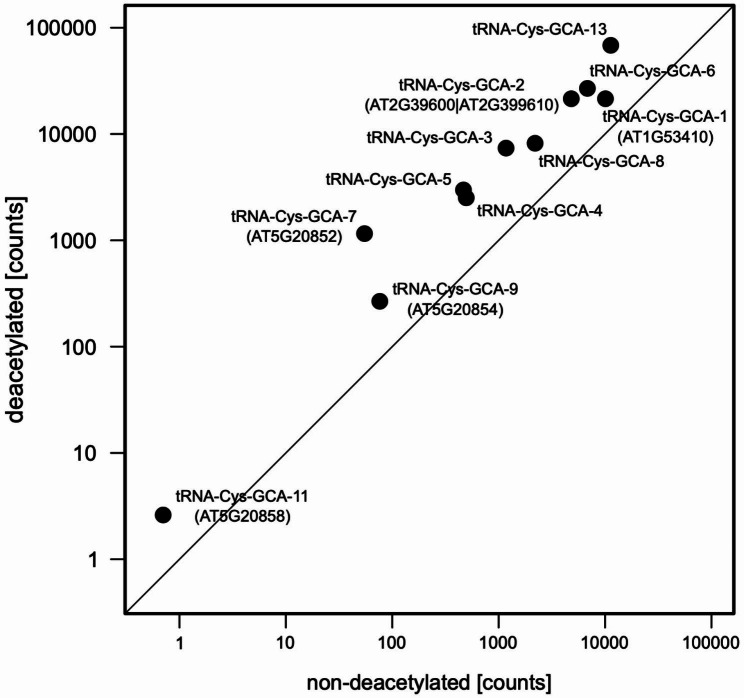
Normalized read counts for tRNA-Cys-GCA gene variants in Arabidopsis thaliana Col-0, for both deacylated and non-deacylated (control) samples (for supporting data see Supplementary Table T3). The transcripts (tRNA-Cys-GCA-12, tRNA-Cys-GCA-13 and the chromosome 5 cluster pseudogene) showing zero counts in either library are not shown. The diagonal line represents 1:1 counts ratio between libraries

An analysis of the tRNAScan-SE predictions of tRNA genes in the genomic sequences of various *A. thaliana* accessions revealed additional, previously not described clusters of tRNA-Cys genes. In the TAIR10 genome, there is a short region on chromosome 2 containing two tRNA-Cys genes in the head-to-head orientation separated by a 134 bp spacer (Fig. 4A). Both genes encode the same sequence variant of tRNA which shows evidence of high expression in the high-throughput sequencing data (Fig. 5 and Supplementary Table T3). The mature tRNAs transcribed from both genes would have identical sequences and it is impossible to discriminate between transcripts produced from either of them.

A survey of the genomic sequences from different *A. thaliana* accessions demonstrated that such an arrangement of the two genes is highly conserved in all genomes analyzed in this study. There is very little variation in the tRNA-coding sequences in this mini-cluster, represented by only three tRNA sequence variants with single substitutions within the T or acceptor stems. In Cvi genome, one of the tRNA genes shows a deletion of 11 nucleotides within the region corresponding to the T-loop and two base pairs of the T-stem (the sequence alignment of the cluster is presented in Supplementary Figure S4).

The second mini-cluster of tRNA-Cys genes was identified on chromosome 1 overlapping in antisense orientation the second intron of the gene encoding ubiquitin domain protein (AT1G53400). In the reference TAIR10 genome, there is only one gene, but in eight of the analyzed genomes, we have detected two tandemly arranged tRNA-Cys genes separated by a short (120 bp) spacer (Fig. 4B and **C**). We confirmed by PCR the size polymorphism of these genomic regions in tested *A. thaliana* accessions (Fig. 4D). In the genomes in which there is only one tRNA-Cys gene, elements of the spacer and sequences flanking the missing gene are still present. The sequence of the AT1G53410 gene in TAIR10 is identical to the sequence of another copy of the tRNA-Cys gene on chromosome 1. The sequencing data show that the signal corresponding to either of these genes accounts for ~ 13% of all tRNA-Cys reads, but it is impossible to determine whether these reads are the products of transcription of both genes or only one of them.

Most of the tRNA sequences within this region can form a canonical tRNA secondary structure. In the accessions Ty-1, Cdm-0, Cvi, and KBS-Mac-74, in which there are two tRNA-Cys gene copies, one of them shows single nucleotide substitutions resulting in mispairing within the T-stem (Ty-1, Cdm-0, Cvi) or acceptor stem (KBS-Mac-74). In these cases, the sequence of the other gene copy can form a perfect tRNA structure. For details on the arrangement of the genes and sequence variation see the supplementary file with multiple sequence alignment of the syntenic regions from all analyzed genomes (Supplementary Figure S5).

### The expression can not be detected for only 2 out of the 13 tRNA-Cys sequence variants in *A. thaliana* Col-0

In the TAIR10 reference sequence of the *A. thaliana* genome, there are 16 predicted tRNA-Cys-GCA genes represented by 13 unique mature tRNA sequence variants [14]. Using the high throughput tRNA sequencing data (PRJNA505412) we investigated the expression levels of all the predicted tRNA-Cys variants (Fig. 5). The comparison of the sequencing results from the libraries constructed with deacylated and control tRNAs showed detectable expression of all, except for tRNA-Cys-GCA-11 (AT5G20858) and tRNA-Cys-GCA-12 (AT5G20856) (Supplementary Table T3). Both transcriptionally silent genes are located within the tRNA-Cys gene cluster on chromosome 5. For the other two genes, tRNA-Cys-GCA-9 (AT5G20854) and tRNA-Cys-GCA-7 (AT5G20852), low-level expression was detected. The tRNA-Cys-GCA-9 transcript can be encoded by an additional gene on chromosome 4 whose expression could account for the observed mature tRNA reads. An analysis of all the reads, including those representing unprocessed precursors that can discriminate between transcripts of these two genes, demonstrates that the tRNA-Cys-GCA-9-2 gene in the cluster is in fact silent. However, the reads matching the tRNA-Cys-GCA-7 variant can only be attributed to the expression of the last tRNA-Cys gene in the cluster. The higher level of transcripts detected in the deacylated libraries (Fig. 5) indicates that the tRNAs are aminoacyl-charged and likely involved in protein translation.

### The tRNA-Cys gene clusters are very variable in other Arabidopsis species

To check if the clustered arrangement of the tRNA-Cys genes is a conserved feature, we surveyed the available genomes from four other species belonging to the genus Arabidopis: *A. halleri* (GCA_003711535.1) [15], *A. lyrata* (GCF_000004255.2) [16], *A. suecica* (GCA_905175345.1), and *A. arenosa* (GCA_905175405.1) [17]. We could identify the entire clusters or individual repeat elements in different assemblies of genomic sequences from all four species.

The single repeating units of the chromosome 5 cluster could be found in all genomes. In *A. lyrata*, *A. halleri*, and *A. arenosa* the fragments consisting of the spacer region with one or two flanking tRNA genes were found. The largest cluster, consisting of four repeating units, was identified in the *A. suecica* genome. The sequence of this region shows a high sequence identity with the sequence from *A. thaliana*. This finding suggests that the amplification of the cluster occurred in *A. thaliana* genome before the origin of *A. suecica* which is a hybrid between *A. thaliana* and *A. arenosa*.

The mini-cluster of tRNA-Cys genes on chromosome 2 is conserved in *A. halleri*, *A. suecica*, and *A. arenosa* where, apart from the differences in the nucleotide sequences, there are no structural variations between different species. We could not identify the entire cluster in either of the available genomic sequences from *A. lyrata* in which a single gene is present.

**Fig. 6:**
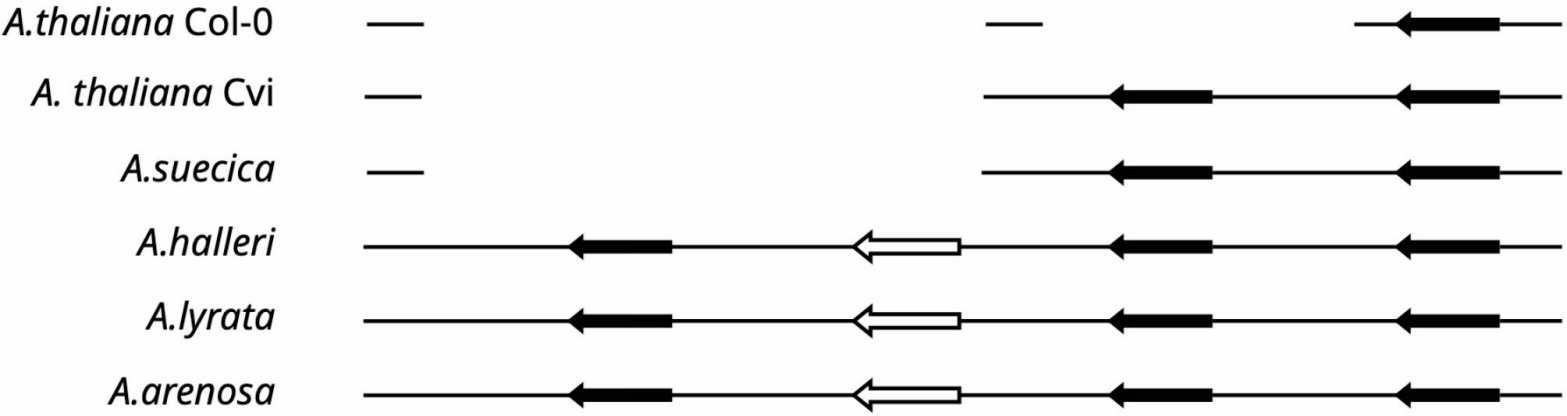
The structures of the tRNA-Cys clusters corresponding to the A. thaliana cluster on chromosome 1. The clusters from A. suecica, A. halleri, A. lyrata, and A. arenosa are compared with corresponding clusters from A. thaliana Col-0 and Cvi. The pseudogenes are shown as open arrows

The second mini-cluster overlapping the intronic sequence of the *A. thaliana* AT1G53400 gene is also present in all analyzed genomes and consists of at least two genes as in the case of eight *A. thaliana* ecotypes discussed above. However, in *A. halleri*, *A. lyrata*, and *A. arenosa*, the cluster is expanded and includes an additional tRNA-Cys gene and a pseudogene (Fig. 6). The alignment comparing sequences of this cluster from different *Arabidopis* species is a part of the supplementary material (Supplementary Figure S6). The sequence similarity, including the conserved anticodon loop, suggests that the pseudogene is derived from the tRNA-Cys-GCA gene. The distances between the additional copies also indicate that the larger cluster was a result of the duplication of a fragment containing the entire pair of tRNA-Cys genes (the min-cluster preserved in some *A. thaliana* ecotypes).

## Discussion

In this study, we analyzed the clustered arrangement of the tRNA-Cys genes in the genomic sequences of various *A. thaliana* accessions. In addition to the previously described cluster on chromosome 5, we identified two additional mini clusters on chromosomes 1 and 2. Two of the analyzed regions, on chromosome 5 and chromosome 1 show heterogeneity in the number of tRNA gene copies between different ecotypes.

The tRNA-CysGCA cluster on chromosome 5 is the smallest of tRNA clusters initially identified in the *A. thaliana* genome with a relatively small size of the repeating units (~ 420 bp). The comparison of regions identified by tRNAscan-SE showed that the majority of tRNA genes within the tRNA-Cys clusters may represent functional genes, classified as ‘high confidence’. Only one of the regions was classified as a pseudogene. The comparison of the syntenic regions from the complete genomic sequences of various *A. thaliana* accessions suggested that there is significant structural heterogeneity between different ecotypes. In genomic sequences, we identified variants consisting of four to six repeating units. A common feature in all the analyzed genomes is the presence of a tRNA-Cys pseudogene in the 5’-flanking region (relative to the direction of transcription). In all cases, the regions flanking the cluster are almost identical. A striking feature of the chromosome 5 cluster is a regular pattern of variation. The genomic sequences always differ by the presence or absence of the entire repeating unit.

Earlier studies on the expression of clustered tRNA genes in *A. thaliana* suggested that all of them are transcriptionally silent. The silencing of all four tRNA clusters was attributed to increased DNA methylation and the presence of histone modifications associated with transcriptionally repressed heterochromatic regions [6]. Thus, despite the presence of intact promoter elements and Pol III termination signals, the genes within the chromosome 5 tRNA-Cys cluster were presumed to be transcriptionally inactive. Our high-throughput tRNA sequencing data analysis confirmed that three of the four genes in the reference strain Col-0 are not transcribed, but we observed sequencing reads corresponding to the last tRNA-Cys gene in the cluster. The presence of sequencing reads in both deacylated and non-deacylated tRNA libraries also suggests that the product of this gene is correctly processed and aminoacylated. However, when compared with other tRNA-Cys loci in the genome, the expression of this gene is relatively low. These results suggest that despite epigenetic modifications of DNA and histones associated with transcriptionally silent chromatin in this region, the silencing effects may be less efficient closer to the boundary of the cluster.

The high sequence similarity of the tRNA sequences in the clusters on *(A) thaliana* chromosomes 1 and 2, including the regulatory sequences (boxes A and B) [3], suggests that the mechanisms of their transcription regulation are conserved and similar to those observed in the Col-0 accession. The more divergent tRNA sequences in the cluster on chromosome 5 show single nucleotide deviations from the consensus sequence in some cases. Most likely, these differences are the result of transcription silencing of these genes and random accumulation of sequence substitutions. Interestingly, the transcriptionally active AT5G20852 gene also has a single nucleotide substitution in the sequence corresponding to box (B) This sequence change, along with epigenetic modifications, may be responsible for the lower transcriptional activity of this gene.

An interesting feature of the chromosome 5 tRNA-Cys cluster is the presence of the putative transposable elements. According to the TAIR10 annotation, the tRNA-Cys cluster overlaps with three transposable elements belonging to the Helitron family. The high sequence similarity of those regions across all tested accessions indicates that this genomic region has conserved properties. It has been suggested that the TEs from this group transpose using a rolling circle mechanism via a single-stranded DNA intermediate, analogous to the rolling-circle mechanism involved in the propagation of bacterial transposons. Helitrons encode a Y2-type tyrosine recombinase responsible for catalytic activity associated with transposition, and at the 5’- and 3’-ends they have conserved TC and CTRR sequences, respectively. In addition, the Helitron TEs frequently have a short palindromic sequence located very close to the 3’ end [9].

Helitrons have been reported to carry genomic fragments from different chromosomal locations. However, in most of the studies, only protein-coding genes and gene fragments were reported as potential cargo. If the Helitron transposition were in fact involved in the amplification of the tRNA-Cys cluster, it would represent the first example of the non-coding RNA captured by a Helitron family transposon.

The requirement of the Helitron replication process for the hairpin structure at the 3’ termini and the presence of tRNA in this location, open a tempting opportunity for speculation about the possible role of this tRNA in its replication process. As a first option, the tRNA structure could be used as a strong termination signal during amplification. The alternative scenario would assume that the presence of a strong tRNA structure close to the 3’ end of the transposon disturbs the hairpin terminator. In addition, we have conducted a manual search for TC/CTRR signals (data not shown) and found at least one CTAG motif followed by T or C for each of the repeating units containing one tRNA gene. Such an arrangement of the Helitron conserved signals supports the tandem copy duplication mode of the tRNA-Cys containing TEs. It has been reported for bacterial *IS91* elements that only one end is necessary to initiate transposition. Such one-ended transposition, in the absence of a precise termination signal, results in the generation of tandem copies of the donor plasmid [18]. Hence, failure to recognize the termination signal for Helitron transposition may result in local duplication of the element. In such a case, the tRNA-Cys cluster could be regarded as an array of short Helitron elements with a captured tRNA-Cys gene preventing the formation of the correct termination signal for transposition.

The structural variation of the chromosome 1 tRNA-Cys cluster can be explained by two alternative evolutionary models. The first one assumes the presence of the four-gene cluster in the common ancestor of all tested Arabidopsis species that is preserved in the *A. halleri*, *A. lyrata*, and *A. arenosa* lineages. In this model, the cluster would have undergone a reduction in the *A. thaliana* lineage leading to the origin of the two- or one-gene arrangements. In some cases, such reduction may have even included only a part of the tRNA gene. A second model would assume the presence of the two-gene cluster in the common ancestor that underwent a reduction in the *A. thaliana* genome and expansion of the whole ancestral cluster in the *A. halleri*, *A. lyrata*, and *A. arenosa* lineage.

In addition, regardless of the evolutionary pathways and molecular mechanisms involved in the creation of the observed variability of the tRNA-Cys clusters, the presented study expands the repertoire of the reference tRNA genes and transcripts that can be assigned to the *A. thaliana*.

## Methods

### Sources of genomic data

The genomic sequences for various accessions of *A. thaliana* exclusively sequenced by the long-read NGS technologies were downloaded from the NCBI Assembly database (https://www.ncbi.nlm.nih.gov/assembly/) [19]. The syntenic regions of chromosome 5 corresponding to the Cys-tRNA gene cluster on chromosome 5 in the reference TAIR10 genome were identified by BLAST [20]. In addition, we investigated the presence of homologous protein-coding flanking (AT2G39590/AT2G39620 for chromosome 2 and AT5G20850/AT5G20860 for chromosome 5) or overlapping (AT1G53400 for chromosome 1) genes using a reciprocal best hit approach to establish the synteny relationship between the tested genomes. Only the unique sequences for particular accessions were retained. The data were further filtered to eliminate low-quality sequences based on the content of ambiguous (non-A/T/C/G) positions. The sequences containing over 1% of ambiguous positions were removed from the data set. The summary of the sources of genomic sequence in the final dataset used for particular accessions is shown in Supplementary Table T1.

### Predictions of tRNA genes

The regions of the tRNA genes were predicted using tRNAscan-SE 2.0 software [8]. The results of predictions were validated using the EukHighConfidenceFilter included in the tRNAscan-SE package to identify regions that can be regarded as high confidence tRNA genes and regions potentially encoding tRNAs that do not entirely conform to the canonical tRNA structure (secondary or tertiary) or are likely tRNA pseudogenes.

### Prediction of Helitron transposons

For the prediction of Helitron transposable elements, we have used the genomic sequence fragment corresponding to the tRNA-Cys cluster extended with 500 bp flanking regions on each site. The annotation procedures were also conducted on the whole chromosome 5. In each case, both strands were searched for the presence of the Helitrons. The predictions were generated with HelSearch [11], HelitronScanner [12], and EAHelitron [13] using in each case the default parameters. The results of the annotation were compared with a gff3 file containing complete annotation (genes and pseudogenes) of the tRNA-Cys cluster.

### Sequence analysis

For the comparative analysis of the genomic regions and the generation of the dot plots, we used the FlexiDot software [21]. Clustering of the tRNA gene sequences based on the identity was performed with USEARCH *cluster_fast* command [22].

The sequence alignments were created using ClustalW [23]. For all phylogenetic analyses of the genomic sequences and generation of phylogenetic trees, MEGA X software was used [24]. The entire chromosome 5 tRNA cluster regions, including 150 bp flanking regions, were used for alignments and construction of the tree, with the neighbor-joining method, presented in Fig. 3A. The pairwise identity values between sequences were calculated from the global alignments using the *needle* program from the EMBOSS package [25, 26].

### Assessing the tRNA expression levels from the high-throughput sequencing data

The expression data were downloaded from the NCBI SRA database [27] project number PRJNA505412. The download files included two replicates of control and deacylated RNAs isolated from 2-week-old Arabidopsis seedlings.

After isolating RNA using Zymo-Spin™ IIC Columns (Zymo Research) and a solution mixture of D/acidic phenol/chloroform (Ambion, AM9720) (5:1), the sample was loaded onto a column, washed three times, and then treated in-column with 50 μl of 0.5 M Tris-HCl pH 9.5 at 42°C for 1 hour. A non-deacylated control sample was eluted from the column directly after washes. Following deacylation, the RNA sample was separated in a 12% PAA gel with 8 M urea to extract the tRNA fraction (65–100 nucleotides in length). tRNA species were dephosphorylated using 1U of Fast AP alkaline phosphatase (Thermo Fisher Scientific) at 37°C for 10 minutes. The tRNAs were then ligated to a 5’-end pre-phosphorylated 21 nt RNA adapter with a modified 3’ end (UGG AAU UCU CGG GUG CCA AGG–S-C3). The ligation products (86–121 nt) were purified by PAGE and then subjected to reverse transcription in the presence of 15 pmol of SMART-like RNA oligo (modified for compatibility with the Illumina TruSeq sequencing system: m1GCCUACACGACGCUCUUCCGAUCUAUGGG) and 120 U of SmartScribe Reverse Transcriptase (Clontech). The cDNA sample was PCR-enriched using Phusion HF DNA Polymerase (NEB) with primers complementary to Illumina-compatible sequencing adapters present at both ends of tRNAs. Finally, the indexed samples were pooled equimolarly for single-read (125 nt) sequencing on the Illumina HiSeq 2500 system. The tRNA libraries were sequenced with a custom-made extended sequencing primer by Fasteris SA (Switzerland).

The data set was reduced to unique sequences and reads’ counts were normalized using the “Trimmed Mean of M-values” (TMM) method [28]. The fraction of sequences representing transcripts of 55–110 nt was used for the filtration procedure that included two major steps: (i) contamination and (ii) non-specific fragments cleaning. The first stage consists of a BLAST-based similarity search *(-word_size 6 -perc_identity 65 -dust no -soft_masking false -outfmt 6*).

of all sequences versus the NCBI non-redundant nucleotide database (as of December 2020). At this step, all fragments that did not match any sequences from species classified in the mustard family (*Brassicaceae*) were removed. Subsequently, the remaining sequences were searched with BLAST (with the same parameters) using the evaluated set of tRNAs sequences as reference. At each step of data analysis only Linux text processing tools, in-house generated Perl scripts and AWK language commands were used (https://github.com/wmkone/trna-cys). Exclusively only the best matching (based on cumulative BLAST score), uniquely mapped fragments were assigned to corresponding tRNA genes.

### Confirmation of cluster sizes by genomic PCR

PCR reactions were conducted using Thermo Scientific Phire Plant Direct PCR Master Mix (F160S) and the dilution protocol described in the manual. The following primer pairs were used to amplify the clusters located on chromosomes 1 and 5, respectively: 5’CACTGACACAATGTAGTTGCC3’ / 5’CCTTGAGCCCTTAGGAATGAG3’ and 5’CTGATCGTTTGACTTGACACG3’ / 5’ATGGTACCTAGGTGTTTGACC3’. Both amplification reactions included the following steps: 1 min at 98 °C, 30 repetitions of the following incubation sequence: 5 s at 98 °C, 5 s at 62.3 °C, 20 s at 72 °C and a final incubation for1 minute at 72 °C.

### Electronic supplementary material

Below is the link to the electronic supplementary material.

**Additional file 1: Supplementary Table T1**. Sources of genomic sequences used in the analyses. The accession numbers refer to data available from the NCBI Assembly Database. **Supplementary Table T2**. Locations of the tRNACys clusters on chromosome 5 in the genomic sequences. **Supplementary Table T3**. Normalized read counts for tRNA-Cys-GCA gene variants in Arabidopsis thaliana Col-0, for both deacylated and non-deacylated samples. The averages represent the normalized read count values from two libraries

**Additional file 2: Supplementary Figure S1**. Phylogenetic tree of the tRNACys chromosome 5 cluster repeats from the analyzed ecotypes. The tree was inferred using the Neighbor-Joining method. The branches are labeled with the percentages of trees in which the taxa clustered together in the bootstrap test with 1000 replicates. The evolutionary distances were computed using the Maximum Composite Likelihood method using MEGA X software. The multiple sequence alignment supporting the tree is provided in Supplementary Figure S9. **Supplementary Figure S2**. Phylogenetic tree of the tRNA sequences from the chromosome 5 cluster repeats. The consensus tree was inferred using the Neighbor-Joining method. The labels indicate repeat numbers containing particular tRNA sequence. Branches with identical sequences are shown with gray triangles. Colors of the groups correspond to colors of the boxes designating tRNA genes in Figure 3B of the main text. The multiple sequence alignment supporting the tree is provided in Supplementary Figure S10. **Supplementary Figure S3**. Multiple sequence alignment of the chromosome 5 tRNA-Cys gene clusters. Location of the tRNA genes and pseudogenes are shown in the tRNA genes line as green and red boxes, respectively. **Supplementary Figure S4**. Multiple sequence alignment of the tRNA-Cys gene mini-clusters on chromosome 2. Location of the tRNA genes are shown in the tRNA genes line as green boxes. **Supplementary Figure S5**. Multiple sequence alignment of the Arabidopsis thaliana tRNA-Cys gene mini-clusters on chromosome 1. Location of the tRNA genes are shown in the tRNA genes line as green boxes, Note that the orientation of of the genes is reversed relative to the orientation shown in Fig. 4B. **Supplementary Figure S6**. Multiple sequence alignment of the tRNA-Cys gene mini-clusters on chromosome 1 from various Arabidopsis species. Location of the tRNA genes and pseudogenes are shown in the tRNA genes line as green and red boxes, respectively. Note that the orientation of of the genes is reversed relative to the orientation shown in Fig. 5. **Supplementary Figure S7**. Picture of the full-length gel presented in Figure 3C: PCR-based analysis of the size heterogeneity of the chromosome 5 tRNA-Cys clusters (see main text for details). **Supplementary Figure S8**. Picture of the full-length gel presented in Figure 4D: experimental evaluation of the chromosome 1 tRNA-Cys cluster region from various A. thaliana accessions (see main text for details). **Supplementary Figure S9**. Multiple sequence alignment of chromosome 5 cluster repeats used to construct the tree in Supplementary Figure S1. **Supplementary Figure S10**. Multiple sequence alignment of chromosome 5 cluster tRNA sequences used to construct the tree in Supplementary Figure S2

## Data Availability

All the analyses were performed on the genomic sequences of *A. thaliana* available from public databases: the NCBI Assembly Database (https://www.ncbi.nlm.nih.gov/assembly/). The sources of particular sequences and the NCBI Assembly database accession numbers are given in the Supplementary material Table T1. The data used to analyze tRNA expression were downloaded from the NCBI SRA database (https://www.ncbi.nlm.nih.gov/sra) project number PRJNA505412.
