# Supplementary material for: tRNA-Cys gene clusters exhibit high variability in Arabidopsis thaliana
Source: BMC Plant Biol. 2023 Dec 7;23:623. doi: 10.1186/s12870-023-04632-x (PMC10701932; doi:10.1186/s12870-023-04632-x)
Supplement: Supplementary file 1 — Additional file 1: Supplementary Table T1. Sources of genomic sequences used in the analyses. The accession numbers refer to data available from the NCBI Assembly Database. Supplementary Table T2. Locations of the tRNACys clusters on chromosome 5 in the genomic sequences. Supplementary Table T3. Normalized read counts for tRNA-Cys-GCA gene variants in Arabidopsis thaliana Col-0, for both deacylated and non-deacylated samples. The averages represent the normalized read count values from two libraries [file 12870_2023_4632_MOESM1_ESM.docx]

**Supplementary Table T1**. Sources of genomic sequences used in the analyses. The accession numbers refer to data available from the NCBI Assembly Database.

| Accession | Ecotype name | BioProject | Reference |
| --- | --- | --- | --- |
| GCF_000001735.4 | Col-0 (TAIR10) | PRJNA116 |  |
| GCA_902460305.1 | Kyo | PRJEB31147 | [1] |
| GCA_902460315.1 | Eri-1 | PRJEB31147 | [1] |
| GCA_902460285.1 | Ler | PRJEB31147 | [1] |
| GCA_902460265.3 | An-1 | PRJEB31147 | [1] |
| GCA_902705455.1 | C24 | PRJEB31147 | [1] |
| GCA_903064295.1 | Ty-1 | PRJEB37258 |  |
| GCA_903064285.1 | KBS-Mac-74 | PRJEB37257 |  |
| GCA_902460275.1 | Cvi-0 | PRJEB31147 | [1] |
| GCA_903064325.1 | Kn-0 | PRJEB37260 |  |
| GCA_904420315.1 | Cdm-0 | PRJEB40125 | [2] |
| GCA_024498435.1 | 9470 (Tur-4?) | PRJNA779205 | [3] |
| GCA_024498455.1 | 9412 (Kor 3) | PRJNA779205 | [3] |
| GCA_024498555.1 | 1254 (Tos-82-387) | PRJNA779205 | [3] |
| GCA_024498475.1 | 6024 (Fly2-2) | PRJNA779205 | [3] |
| GCA_024498495.1 | 6021 (Fjä2-4) | PRJNA779205 | [3] |

[1] Jiao WB, Schneeberger K (2020) Chromosome-level assemblies of multiple *Arabidopsis* genomes reveal hotspots of rearrangements with altered evolutionary dynamics. *Nat Commun* 11, 989.

[2] Barragan AC et al (2021) A Truncated Singleton NLR Causes Hybrid Necrosis in Arabidopsis thaliana**.** *Mol Biol Evol* 38, 557.

[3] Benjamin Jaegle B et al (2021) Extensive gene duplication in Arabidopsis revealed by pseudo-heterozygosity. bioRxiv 2021.11.15.468652; doi: https://doi.org/10.1101/2021.11.15.468652.

**Supplementary Table T2**. Locations of the tRNACys clusters on chromosome 5 in the genomic sequences.

| **Ecotype** | **Contig Acc. No.** | **Cluster length** | **Begin** | **End** | **strand** |
| --- | --- | --- | --- | --- | --- |
| TAIR10 | NC_003076.8 | 1728 | 7075257 | 7073530 | minus |
| C24 | CACSHJ010000096.1 | 2544 | 7062133 | 7059590 | minus |
| Cdm-0 | LR881470.1 | 2130 | 7075626 | 7073497 | minus |
| Cvi | LR699764.1 | 1994 | 7060886 | 7058893 | minus |
| Eri-1 | LR699769.1 | 1696 | 7076479 | 7074784 | minus |
| Fja-2-4 | CP086748.1 | 2131 | 7030735 | 7028605 | minus |
| Fly-2-2 | CP086743.1 | 1727 | 7067256 | 7065530 | minus |
| KBS-Mac-74 | LR797806.1 | 2543 | 7088423 | 7085881 | minus |
| Kn-0 | LR797811.1 | 2132 | 7049266 | 7047135 | minus |
| Kor3 | CP086738.1 | 1718 | 7069219 | 7067502 | minus |
| Kyo | LR699759.1 | 1718 | 7007685 | 7005968 | minus |
| Ler | LR699774.1 | 2130 | 7042151 | 7040022 | minus |
| Tos82-387 | CP086758.1 | 2540 | 7043780 | 7041241 | minus |
| Tur4 | CP086733.1 | 1727 | 7059953 | 7058227 | minus |
| Ty-1 | LR797801.1 | 1728 | 7060428 | 7058701 | minus |
| Ler | LR699774.1 | 2130 | 7042151 | 7040022 | minus |

**Supplementary Table T3.** Normalized read counts for tRNA-Cys-GCA gene variants in Arabidopsis thaliana Col-0, for both deacylated and non-deacylated samples. The averages represent the normalized read count values from two libraries.

| tRNA-Cys(GCA) variant | GtRNAdb gene symbol* | TAIR gene symbol | tRNA gene cluster | Deacylated libraries | | Non-deacylated libraries | |
| --- | --- | --- | --- | --- | --- | --- | --- |
|  |  |  |  | average | % tRNA-Cys | average | % tRNA-Cys |
| tRNA-Cys-GCA-1 | tRNA-Cys-GCA-1-1 tRNA-Cys-GCA-1-2 | AT1G53410  ‒ | chr1  ‒ | 21519.8 | 13.36 | 10108.7 | 26.91 |
| tRNA-Cys-GCA-6 | tRNA-Cys-GCA-6-1 | ‒ | ‒ | 26932.0 | 16.73 | 6835.3 | 18.19 |
| tRNA-Cys-GCA-2 | tRNA-Cys-GCA-2-1 tRNA-Cys-GCA-2-2 | AT2G39600  AT2G39610 | chr2  chr2 | 21524.7 | 13.37 | 4820.1 | 12.83 |
| tRNA-Cys-GCA-13 | tRNA-Cys-GCA-13-1 | ‒ | ‒ | 68481.1 | 42.54 | 11337.9 | 30.18 |
| tRNA-Cys-GCA-4 | tRNA-Cys-GCA-4-1 | ‒ | ‒ | 2514.3 | 1.56 | 495.9 | 1.32 |
| tRNA-Cys-GCA-5 | tRNA-Cys-GCA-5-1 | ‒ | ‒ | 2981.4 | 1.85 | 466.9 | 1.24 |
| tRNA-Cys-GCA-9 | tRNA-Cys-GCA-9-1 tRNA-Cys-GCA-9-2 | ‒  AT5G20854 | ‒ chr5 | 266.9 | 0.16 | 76.3 | 0.20 |
| tRNA-Cys-GCA-8 | tRNA-Cys-GCA-8-1 | ‒ | ‒ | 8196.1 | 5.09 | 2200.7 | 5.85 |
| tRNA-Cys-GCA-3 | tRNA-Cys-GCA-3-1 | ‒ | ‒ | 7379.1 | 4.58 | 1174.2 | 3.13 |
| tRNA-Cys-GCA-7 | tRNA-Cys-GCA-7-1 | AT5G20852 | chr5 | 1157 | 0.71 | 54.8 | 0.15 |
| tRNA-Cys-GCA-12 | tRNA-Cys-GCA-12-1 | AT5G20856 | chr5 | 0.9 | 0 | 0.0 | 0 |
| tRNA-Cys-GCA-11 | tRNA-Cys-GCA-11-1 | AT5G20858 | chr5 | 2.6 | 0 | 0.7 | 0 |
| tRNA-Cys-GCA-14 | tRNA-Cys-GCA-14-1 | ‒ | ‒ | 0.0 | 0 | 0.0 | 0 |
| CysGCA.pseudo** | ‒ | ‒ | chr5 | 0.0 | 0 | 0.0 | 0 |

*) The multiple sequence IDs from GtRNAdb database represent the same mature tRNA transcript sequence encoded by distinct genes.

**) The pseudogene from chromosome 5 tRNA-Cys genes cluster is not predicted by tRNAScan-SE in TAIR10 genomic sequence but was identified by sequence similarity with other tRNA-Cys genes.
