# Supplementary material for: tRNA-Cys gene clusters exhibit high variability in Arabidopsis thaliana
Source: BMC Plant Biol. 2023 Dec 7;23:623. doi: 10.1186/s12870-023-04632-x (PMC10701932; doi:10.1186/s12870-023-04632-x)
Supplement: Supplementary file 2 — Additional file 2: Supplementary Figure S1. Phylogenetic tree of the tRNACys chromosome 5 cluster repeats from the analyzed ecotypes. The tree was inferred using the Neighbor-Joining method. The branches are labeled with the percentages of trees in which the taxa clustered together in the bootstrap test with 1000 replicates. The evolutionary distances were computed using the Maximum Composite Likelihood method using MEGA X software. The multiple sequence alignment supporting the tree is provided in Supplementary Figure S9. Supplementary Figure S2. Phylogenetic tree of the tRNA sequences from the chromosome 5 cluster repeats. The consensus tree was inferred using the Neighbor-Joining method. The labels indicate repeat numbers containing particular tRNA sequence. Branches with identical sequences are shown with gray triangles. Colors of the groups correspond to colors of the boxes designating tRNA genes in Figure 3B of the main text. The multiple sequence alignment supporting the tree is provided in Supplementary Figure S10. Supplementary Figure S3. Multiple sequence alignment of the chromosome 5 tRNA-Cys gene clusters. Location of the tRNA genes and pseudogenes are shown in the tRNA genes line as green and red boxes, respectively. Supplementary Figure S4. Multiple sequence alignment of the tRNA-Cys gene mini-clusters on chromosome 2. Location of the tRNA genes are shown in the tRNA genes line as green boxes. Supplementary Figure S5. Multiple sequence alignment of the Arabidopsis thaliana tRNA-Cys gene mini-clusters on chromosome 1. Location of the tRNA genes are shown in the tRNA genes line as green boxes, Note that the orientation of of the genes is reversed relative to the orientation shown in Fig. 4B. Supplementary Figure S6. Multiple sequence alignment of the tRNA-Cys gene mini-clusters on chromosome 1 from various Arabidopsis species. Location of the tRNA genes and pseudogenes are shown in the tRNA genes line as green and red boxes, respectively. Note that the o [file 12870_2023_4632_MOESM2_ESM.docx]

**Supplementary Figure S1**. Phylogenetic tree of the tRNACys chromosome 5 cluster repeats from the analyzed ecotypes. The tree was inferred using the Neighbor-Joining method. The branches are labeled with the percentages of trees in which the taxa clustered together in the bootstrap test with 1000 replicates. The evolutionary distances were computed using the Maximum Composite Likelihood method using MEGA X software. The multiple sequence alignment supporting the tree is provided in Supplementary Figure S9.


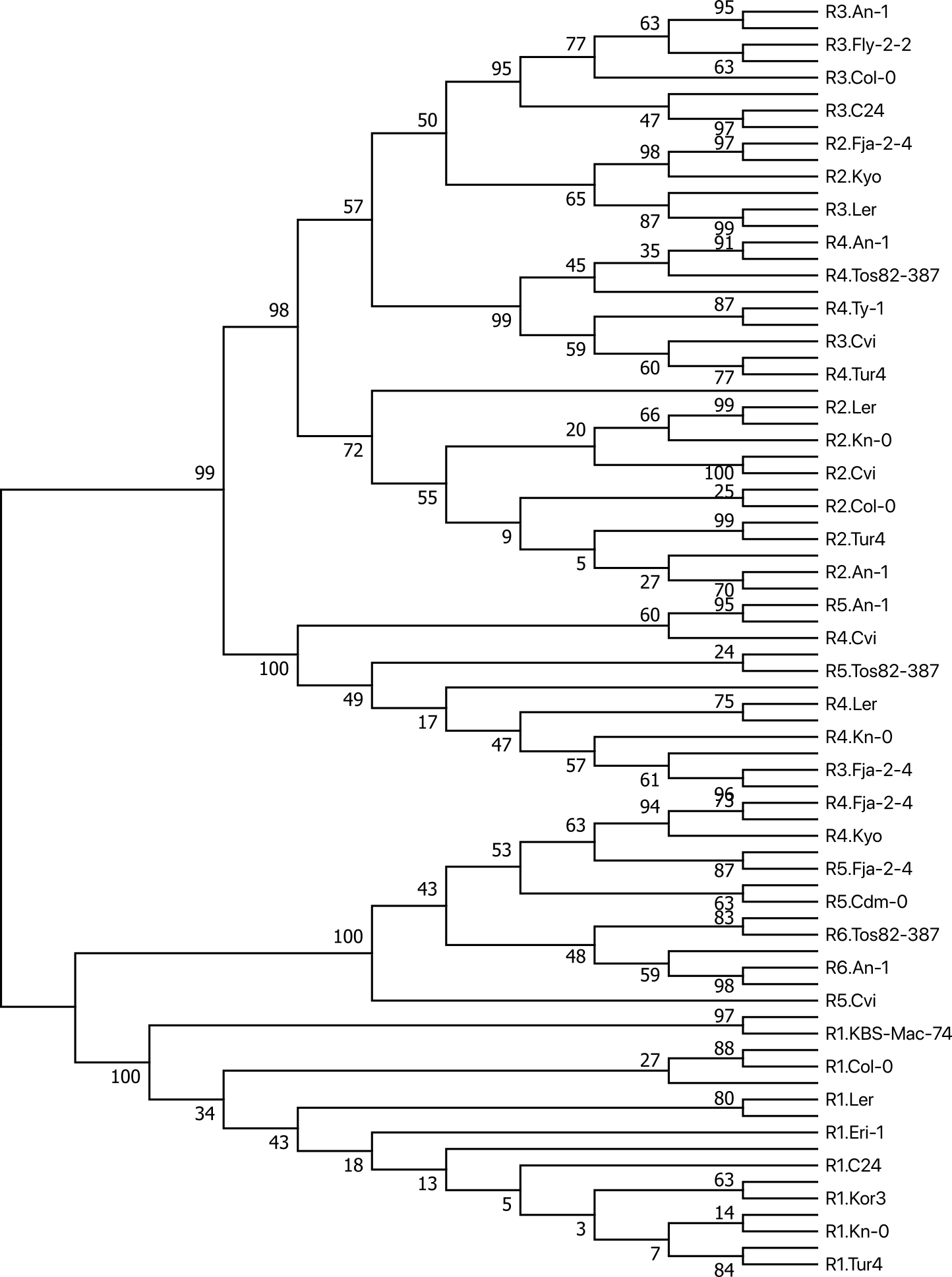


**Supplementary figure S2**. Phylogenetic tree of the tRNA sequences from the chromosome 5 cluster repeats. The consensus tree was inferred using the Neighbor-Joining method. The labels indicate repeat numbers containing particular tRNA sequence. Branches with identical sequences are shown with gray triangles. Colors of the groups correspond to colors of the boxes designating tRNA genes in Figure 3B of the main text. The multiple sequence alignment supporting the tree is provided in Supplementary Figure S10.


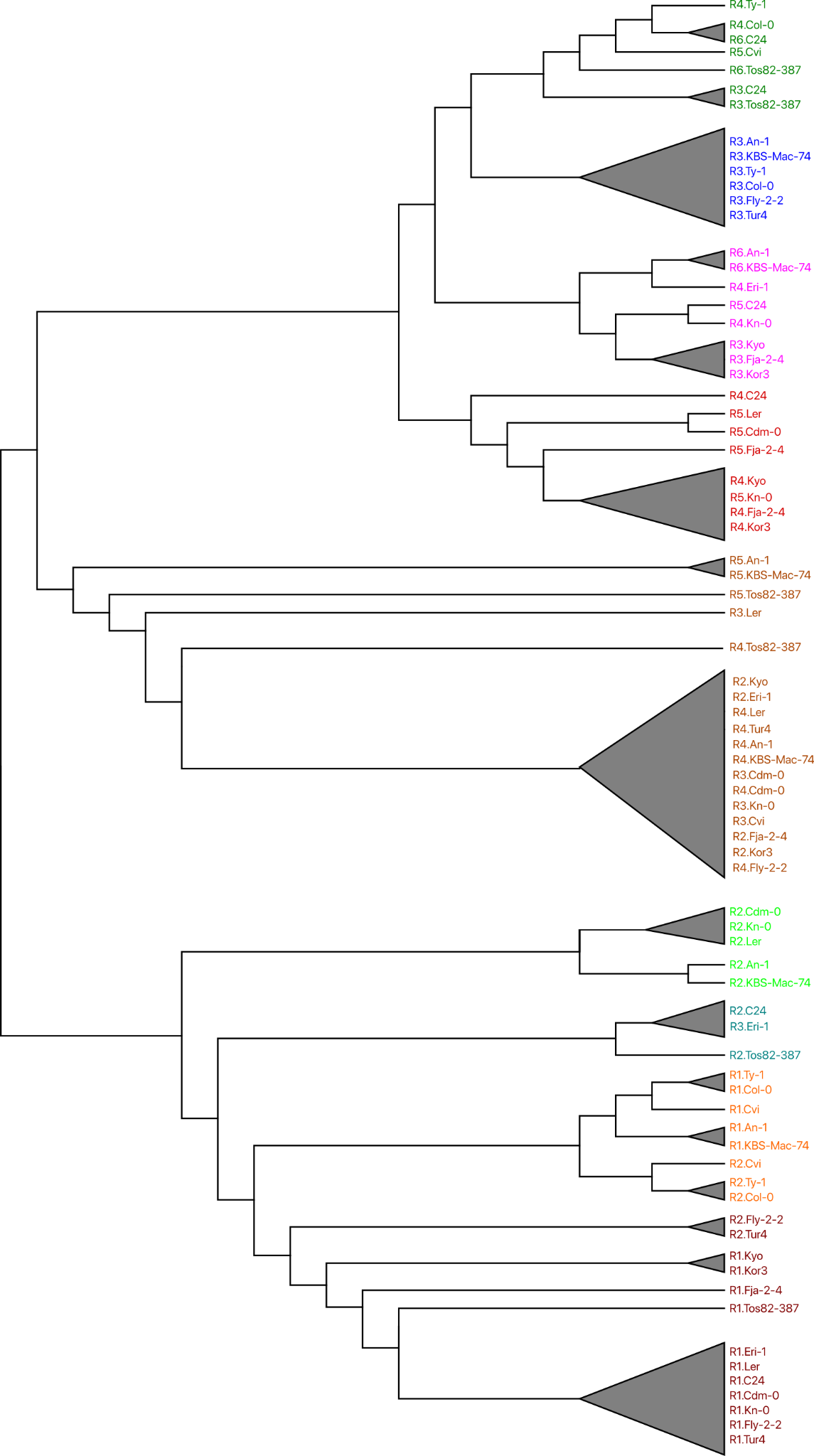


**Supplementary Figure S3**. Multiple sequence alignment of the chromosome 5 tRNA-Cys gene clusters. Location of the tRNA genes and pseudogenes are shown in the tRNA genes line as green and red boxes, respectively.

10 20 30 40 50 60 70 80 90 100

....|....|....|....|....|....|....|....|....|....|....|....|....|....|....|....|....|....|....|....|

**tRNA genes -------------------------------------------------->>>>>>>>>>>>>>>>>>>>>>>>>>>>>>>>>>>>>>>>>>>>>>>>>>**

**Col-0**  **CGTAAGGACTAAAAATAATAATTTAAAACTTTATAATTAATAAAGCAATAAGGTTTATATTTCGGTGGTAAAGCAATTGATTGCAGATCAATAAGTCACC**

**An-1**  **CGTAAGGACTAAAAATACTAATTTAAAACTTTATAATTAATAAAGCAATAAGGTTTATATTTCGGTGGTAAAGCAATTGATTGCAGATCAATAAGTCACC**

**C24**  **CGTAAGGACTAAAAATAATAATTTAAAACTTTATAATTAATAAAGCAATAAGGTTTATATTTCGGTGGTAAAGCAATTGATTGCAGATCAATAAGTCACC**

**KBS-Mac-74** **CGTAAGGACTAAAAATACTAATTTAAAACTTTATAATTAATAAAGCAATAAGATTTATATTTCGGTGGTAAAACAATTGATTGCAGATCAATAAGTCACC**

**Tos82-387**  **CGTAAAGACTAAAAATAATAATTTAAAACTTTATAATTAATAAAGCAATAAGGTTTATATTTTGGTGGTAAAGCAATTGATTGCAGATCAATAAGTCACC**

**Cvi**  **CGTAAGGACTAAAAATAATAATTTAAAACTTTATAATTAATAAAGCAATAAGGTTTATATTTCGGTGGTAAAGCAATTGATTGCAGATCAATAAATCCCC**

**Kn-0**  **CGTAAGGACTAAAAATAATAATTTAAAACTTTATAATTAATAAAGCAATAAGGTGTATATTTCGGTGGTAAAGCAATTGATTGCAGATCAATAAGTCACC**

**Cdm-0**  **CGTAAGGACTAAAAATAATAATTTAAAACTTTATAATTAATAAAGCAATAAGGTGTATATTTCGGTGGTAAAGCAATTGATTGCAGATCAATAAGTCACC**

**Ler**  **CGTAAGGACTAAAAATAATAATTTAAAACTTTATAATTAATAAAGCAATAAGGTGTATATTTCGGTGGTAAAGCAATTGATTGCAGATCAATAAGTCACC**

**Fja-2-4**  **CGTAAGGACTAAAAATAATAATTTAAAACTTTATAATTAATAAAGCAATAAGGTGTATATTTCGGTGGTAAAGCAATTGATTGCAGATCAATAAGTCACC**

**Eri-1**  **CGTAAGGACTAAAAATAATAATTTAAAACTTTATAATTAATAAAGCAATAAGGTTTATATTTTGGTGGTAAAGCAATTGATTGCAGATCAATAAGTCACC**

**Kor3**  **CGTAAGGACTAAAAATAATAATTTAAAACTTTATAATTAATAAAGCAATAAGGTGTATATTTCGGTGGTAAAGCAATTGATTGCAGATCAATAAGTCACC**

**Kyo**  **CGTAAGGACTAAAAATAATAATTTAAAACTTTATAATTAATAAAGCAATAAGGTGTATATTTCGGTGGTAAAGCAATTGATTGCAGATCAATAAGTCACC**

**Tur4**  **CGTAAGGACTAAAAATAATAATTTAAAACTTTATAATTAATAAAGCAGTAAGGTTTATATTTCGGTGGTAAAGCAATTGATTGCAGTTCAATAAGTCACC**

**Ty-1**  **CGTAAGGACTAAAAATAATAATTTAAAACTTTATAATTAATAAAGCAATAAGGTTTATATTTCGGTGGTAAAGCAATTGATTGCAGATCAATAAGTCACC**

**Fly-2-2**  **CGTAAGGACTAAAAATAATAATTTAAAACTTTATAATTAATAAAGCAGTAAGGTTTATATTTCGGTGGTAAAGCAATTGATTGCAGTTCAATAAGTCACC**

110 120 130 140 150 160 170 180 190 200

....|....|....|....|....|....|....|....|....|....|....|....|....|....|....|....|....|....|....|....|

**tRNA genes >>>>>>>>>>>>>>>>>>>>>>------------------------------------------------------------------------------**

**Col-0**  **GTTTTAAATCCGATTAAATCTTATAATTTTTACAGTTTATCAACAATTTTTTATTAGAGCTTTATTTATACATTTGATTAGCTTTTAGCAACTAGTAGCT**

**An-1**  **GTTTTAAATCCGATTAAATCTTATCATTTTTACAGTTTATCAACAATTTTTTATTAGAGCTTTATTTATACATTTGATTAGCTTTTAGCATCTAGTAGCT**

**C24**  **GTTTTAAATCCGATTAAATCTTATAATTTTTACAGTTTATCAACAATTTTTTATTAGAGCTTTATTTATACATTTGATTAGCTTTTAGCATCTAGTAGCT**

**KBS-Mac-74** **GTTTTAAATCCGATTAAATCTTATCATTTTTACAGTTTATCAACAATTTTTTATTAGAGCTTTATTTATACATTTGATTAGCTTTTAGCATCTAGTAGCT**

**Tos82-387**  **GTTTTAAATCCGATTAAATCTTATAATTTTTACAGTTTATCAACAATTTTTTATTAGAGCTTTATTTATACATTTGATTAGCTTTTAGCATCTAGTAGCT**

**Cvi**  **GTTTTAAATCCGATTAAATCTTATAATTTTTACAGTTTATCAACAATTTTTTATTAGAGCTTTATTTATACATTTGATTAGCTTTTAGCAACTAGTAGCT**

**Kn-0**  **GTTTTAAATCCGATTAAATCTTATAATTTTTACAGTTTATCAACAATTTTTTATTAGAGCTTTATTTATACATTTGATTAGCTTTTAGCATCTAGTAGCT**

**Cdm-0**  **GTTTTAAATCCGATTAAATCTTATAATTTTTACAGTTTATCAACAATTTTTTATTAGAGCTTTATTTATACATTTGATTAGCTTTTAGCATCTAGTAGCT**

**Ler**  **GTTTTAAATCCGATTAAATCTTATAATTTGTACAGTTTATCAACAATTTTTTATTAGAGCTTTATTTATACATTTGATTAGCTTTTAGCATCTAGTAGCT**

**Fja-2-4**  **GTTTTAAATCCGATTAAATCTTATAATTTTTACAGTTTATCAACAATTTTTTATTAGAGCTTTATTTATACATTTGATTAGCTTTTAGCATCTAGTAGCT**

**Eri-1**  **GTTTTAAATCCGATTAAATCTTATAA----------------ACAATTTTTTATTAGAGCTTTATTTATACATTTGATTAGCTTTTAGCATCTAGTAGCT**

**Kor3**  **GTTTTAAATCCGATTAAATCTTATAATTTTTACAGTTTATCAACAATTTTTTATTAGAGCTTTATTTATACATTTGATTAGCTTTTAGCATCTAGTAGCT**

**Kyo**  **GTTTTAAATCCGATTAAATCTTATAATTTTTACAGTTTATCAACAATTTTTTATTAGAGCTTTATTTATACATTTGATTAGCTTTTAGCATCTAGTAGCT**

**Tur4**  **GTTTTAAATCCGATTAAATCTTATAATTTTTACAGTTTATCAACAATTCTTTATTAGAGCTTTATTTATACATTTGATTAGCTTTTAGCAACTAGTAGCT**

**Ty-1**  **GTTTTAAATCCGATTAAATCTTATAATTTTTACAGTTTATCAACAATTTTTTATTAGAGCTTTATTTATACAGTTGATTAGCTTTTAGCAACTAGTAGCT**

**Fly-2-2**  **GTTTTAAATCCGATTAAATCTTATAATTTTTACAGTTTATCAACAATTCTTTATTAGAGCTTTATTTATACATTTGATTAGCTTTTAGCAACTAGTAGCT**

210 220 230 240 250 260 270 280 290 300

....|....|....|....|....|....|....|....|....|....|....|....|....|....|....|....|....|....|....|....|

**tRNA genes ----------------------------------------------------------------------------------------------------**

**Col-0**  **AACTAATCTTATATTTCAAGGTTCTAGCTCGCAATATTTCATTTTTAATAAAACATCTTTGACAAGTTTTTGTATATAATTCTTTAGCAAGTGAATATGT**

**An-1**  **AACTAATCTTATATTTCAAGGTTGTAGCTCGCAATTTTTCATTTTTAATAAAACATCTTTGACAAGTTTTTGTATATAATTCTTTAGCAAGTGAATATGT**

**C24**  **AACTAATCTTATATTTCAAGGTTGTACCTCGCAATTTTTCATTTTTAATAAAACATCTTTGACAAGTTTTTGTATATAATTCTTTAGCAAGTGAATATGT**

**KBS-Mac-74** **AACTAATCTTATATTTCAAGGTTGTAGCTCGCAATTTTTCATTTTTAATAAAACATCTTTGACAAGTTTTTGTATATAATTCTTTAGCAAGTGAATATGT**

**Tos82-387**  **AACTAATCTTATATTTCAAGGTTGTAGCTCGCAATTTTTCATTTTTAATAAAACATC------AAGTTTTTGTATATAATTCTTTAGCAAGTGAATATGT**

**Cvi**  **AACTAATCTTATATTTCAAGGTTGTAGCTCGCAATTTTTCATTTTTAATAAAACATCTTTGACAAGTTTTTGTATATAATTCTTTAGCAAGTGAATATGT**

**Kn-0**  **AACTAATCTTATATTTCAAGGTTGTAGCTCGCAATTTTTCATTTTTAATAAAACATCTTTGACAAGTTTTTGTATATAATTCTTTAGCAAGTGAATATGT**

**Cdm-0**  **AACTAATCTTATATTTCAAGGTTGTAGCTCGCAATTTTTCATTTTTAATAAAACATCTTTGACAAGTTTTTGTATATAATTCTTTAGCAAGTGAATATGT**

**Ler**  **AACTAATCTTATATTTCAAGGTTGTAGCTCGCAATTTTTCATTTTTAATAAAACATCTTTGACAAGTTTTTGTATATAATTCTTTAGCAAGTGAATATGT**

**Fja-2-4**  **AACTAATCTTATATTTCAAGGTTGTAGCTCGCAATTTTTCATTTTTAATAAAACATCTTTGACAAGTTTTTGTATATAATTCTTTAGCAAGTGAATATGT**

**Eri-1**  **AACTAATCTTATATTTCAAGGTTGTAGCTCGCAATTTTTCATTTTTAATAAAACATC------AAGTTTTTGTATATAATTCTTTAGCAAGTGAATATGT**

**Kor3**  **AACTAATCTTATATTTCAAGGTTGTAGCTCGCAATTTTTCATTTTTAATAAAACATCTTTGACAAGTTTTTGTATATAATTCTTTAGCAAGTGAATATGT**

**Kyo**  **AACTAATCTTATATTTCAAGGTTGTAGCTCGCAATTTTTCATTTTTAATAAAACATCTTTGACAAGTTTTTGTATATAATTCTTTAGCAAGTGAATATGT**

**Tur4**  **AACTAATCTTATATTTCAAGGTTGTAGCTCGCAATTTTTCATTTTTAATAAAACATCTTTGACAAGTTTTTGTATATAATTCTTTAGCAAGTGAATATGT**

**Ty-1**  **AACTAATCTTATATTTCAAGGTTCTAGCTCGCAATTTTTCATTTTTAATAAAACATCTTTGACAAGTTTTTGTATATAATTCTTTAGCAAGTGAATATGT**

**Fly-2-2**  **AACTAATCTTATATTTCAAGGTTGTAGCTCGCAATTTTTCATTTTTAATAAAACATCTTTGACAAGTTTTTGTATATAATTCTTTAGCAAGTGAATATGT**

310 320 330 340 350 360 370 380 390 400

....|....|....|....|....|....|....|....|....|....|....|....|....|....|....|....|....|....|....|....|

**tRNA genes ----------------------------------------------------------------------------------------------------**

**Col-0**  **TTTTCTTTATAATTTCAAGGTTTAATTTGTTTGTGAAAATTGTTTTTGATAATTTATATGATTACTAAATAAGTAAACAATTGACTTGCTTATATTAGAT**

**An-1**  **TTTTCTTTATAATTTCAAGGTTTAATTTGTTTGTGAAAATTGTTTTTGATAATTTATATGATTACTAAATAAGTAAACAATTGACTTGCTTATATTAGAT**

**C24**  **TTTTCTTTATAATTTCAAGGTTTAATTTGTTTGTGAAAATTGTTTTTGATAATTTATATGATTACTAAATAAGTAAACAATTGACTTGCTTATATTAGAT**

**KBS-Mac-74** **TTTTCTTTATAATTTCAAGGTTTAATTTGTTTGTGAAAATTGTTTTTGATAATTTATATGATTACTAAATAAGTAAACAATTGACTTGCTTATATTAGAT**

**Tos82-387**  **TTTTCTTTATAATTTCAAGGTTTAATTTGTTTGTGAAAATTGTTTTTGATAATTTATATGATTACTAAATAAGTAAACAATTGACTTGCTTATATTAGAT**

**Cvi**  **TTTTCTTTACAATTTCAAGGTTTAATTTGTTTGTGAAAATTGTTTTTGATAATTTATATGATTAGTAAATAAGTAAACAATTGACTTGCTTATATTAGAT**

**Kn-0**  **TTTTCTTTATAATTTCAAGGTTTAATTTGTTTGTGAAAATTGTTTTTGATAATTTATATGATTACTAAATAAGTAAACAATTGACTTGCTTATATTAGAT**

**Cdm-0**  **TTTTCTTTATAATTTGAAGGTTTAATTTGTTTGTGAAAATTGTTTTTGATAATTTATATGATTACTAAATAAGTAAACAATTGACTTGCTTATATTAGAT**

**Ler**  **TTTTCTTTATAATTTGAAGGTTTAATTTGTTTGTGAAAATTGTTTTTGATAATTTATATGATTACTAAATAAGTAAACAATTGACTTGCTTATATTAGAT**

**Fja-2-4**  **TTTTCTTTATAATTTCAAGGTTTAATTTGTTTGTGAAAATTGTTTTTGATAATTTATATGATTACTAAATAAGTAAACAATTGACTTGCTTATATTAGAT**

**Eri-1**  **TTTTCTTTATAATTTCAAGGTTTAATTTGTTTGTGAAAATTGTTTTTGATAATTTATATGATTACTAAATAAGTAAACAATTGACTTGCTTATATTAGAT**

**Kor3**  **TTTTCTTTATAATTTCAAGGTTTAATTTGTTTGTGAAAATTGTTTTTGATAATTTATATGATTACTAAATAAGTAAACAATTGACTTGCTTATATTAGAT**

**Kyo**  **TTTTCTTTATAATTTCAAGGTTTAATTTGTTTGTGAAAATTGTTTTTGATAATTTATATGATTACTAAATAAGTAAACAATTGACTTGCTTATATTAGAT**

**Tur4**  **TTTTCTTTATAATTTCAAGGTTTAATTTGTTTGTGAAAATTGTTTTTGATAATTTATATGATTACTAAATAAGTAAACAATTGACTTGCTTATATTAGAT**

**Ty-1**  **TTTTCTTTATAATTTCAAGGTTTAATTTGTTTGTGAAAATTGTTTTTGATAATTTATATGATTACTAAATAAGTAAACAATTGACTTGCTTATATTAGAT**

**Fly-2-2**  **TTTTCTTTATAATTTCAAGGTTTAATTTGTTTGTGAAAATTGTTTTTGATAATTTATATGATTACTAAATAAGTAAACAATTGACTTGCTTATATTAGAT**

410 420 430 440 450 460 470 480 490 500

....|....|....|....|....|....|....|....|....|....|....|....|....|....|....|....|....|....|....|....|

**tRNA genes ----------------------------------------------------------------->>>>>>>>>>>>>>>>>>>>>>>>>>>>>>>>>>>**

**Col-0**  **TTCTTAGC-AAAAAAACAATTAATGAAATAAACAATTTATGATTTTGAACTTATTAAAGCAATAAAGGTCCATAGCTCAGTGGTAGAGCAATTGACTGCA**

**An-1**  **TTCTTAGC-AAAAAAACAATTAATGAAATAAACAATTTATGATTTTGAAATTATTAAAGCAATAAAGGTCCATAGCTCAGTGGTAGAGCAATTGACTGCA**

**C24**  **TTCTTAGCAAAAAAAACAATTAATGAAATAAACAATTTATGATTTTAAACTTATTAAAGCAATAAAGGTCCATAGCTCAGTGGTAGAGCAATTGACTGCA**

**KBS-Mac-74** **TTCTTAGC-AAAAAAACAATTAATGAAATAAACAATTTATGATTTTGAAATTATTAAAGCAATAAAGGTCCATAGCTCAGTGGTAGAGCAATTGACTGCA**

**Tos82-387**  **TTCTTAGC-AAAAAAACAATTAATGAAATAAACAATTTATGATTTTGAACTTATTAAAGCAATAAAGGTCCATAGCTCAGTGGTAGAGCAATTGATTGCA**

**Cvi**  **TTCTTAGC-AAAAAAACAATTAATGAAATAAACAATTTATGATTTTGAACTTATTAAAGCAATAAAGGTCCATATCTCAGTGGTAGAGCAATTGACTGCA**

**Kn-0**  **TTCTTAGC-AAAAAAACAATTAATGAAATAAACAATTTATGATTTTGAACTTATTAAAGCAATAAAGGTCCATAGCTCAGTGGTAGAGCAATTGACTGCA**

**Cdm-0**  **TTCTTAGC-AAAAAAACAATTAATGAAATAAACAATTTATTATTTTGAACTTATTAAAGCAATAAAGGTCCATAGCTCAGTGGTAGAGCAATTGACTGCA**

**Ler**  **TTCTTAGC-AAAAAAACAATTAATGAAATAAACAATTTATTATTTTGAACTTATTAAAGCAATAAAGGTCCATAGCTCAGTGGTAGAGCAATTGACTGCA**

**Fja-2-4**  **TTCTTAGC-AAAAAAACAATTAATGAAATAAACAATTTATGATTTTGAACTTATTAAAGCAATAAAGGTCCATAGCTCAGTGGTAGAGCAATTGACTGCA**

**Eri-1**  **TTCTTAGC-AAAAAAACAATTAATGAAATAAACAATTTATGATTTTGAACTTATTAAAGCAATAAAGGTCCATAGCTCAGTGGTAGAGCAATTGACTGCA**

**Kor3**  **TTCTTAGC-AAAAAAACAATTAATGAAATAAACAATTTATGATTTTGAACTTATTAAAGCAATAAAGGTTCATAGCTCAGTGGTAGAGCAATTGACTGCA**

**Kyo**  **TTCTTAGC-AAAAAAACAATTAATGAAATAAACAATTTATGATTTTGAACTTATTAAAGCAATAAAGGTTCATAGCTCAGTGGTAGAGCAATTGACTGCA**

**Tur4**  **TTCTTAGC-AAAAAAACAATTAATGAAATAAACAATTTATGATTTTGAACTTATTAAAGCAATAAAGGTCCATAGCTCAGTGGTAGAGCAATTGACTGCA**

**Ty-1**  **TTCTTAGC-AAAAAAACAATTAATGAAATAAACAATTTATGATTTTGAACTTATTAAAGCAATAAAGGTCCATAGCTCAGTGGTAGAGCAATTGACTGCA**

**Fly-2-2**  **TTCTTAGC-AAAAAAACAATTAATGAAATAAACAATTTATGATTTTGAACTTATTAAAGCAATAAAGGTCCATAGCTCAGTGGTAGAGCAATTGACTGCA**

510 520 530 540 550 560 570 580 590 600

....|....|....|....|....|....|....|....|....|....|....|....|....|....|....|....|....|....|....|....|

**tRNA genes >>>>>>>>>>>>>>>>>>>>>>>>>>>>>>>>>>>>>---------------------------------------------------------------**

**Col-0**  **GATCAATAGGTCACCGGTTCGAACCCGATTGGGCCCTATATTTTTTCAGTTT-ACCAATATTTTTTTATAAGAGCTTTAGTTATATATTTGATTAGCATT**

**An-1**  **GATCAATAGGTCACCGGTTTGAACCCGATTGGGCCCTATATTTTTTCAGTTT-ACCAATATTTTTTTATAAGAGCTTTAGTTATATATTTGATTAGCATT**

**C24**  **GATCAATAGGTCACCGGTTCAAACCCGATTGGGCCCTATATTTTTTCAGTTT-ACCAATATTTTTTTATAAGAGCTTTAGTTATATATTTGATTAGCATT**

**KBS-Mac-74** **GATCAATAGGTCACCGGTTTGAACCCGATTGGGCCCTATATTTTTTCAGTTT-ACCAATATTTTTTTATAAGAGCTTTAGTTATATATTTGATTAGCATT**

**Tos82-387**  **GATCAATAGGTCACCGGTTCAAACCTGATTGGGCCCTATATTTTTTCAGTTT-ACCAATATTTTTTTATAAAAGCTTTAGTTATATATTTGATTAGCATT**

**Cvi**  **AATCAATAGGTCACCGGTTCGAACCCGATTGGGCCCTATATTTTTTCAGTTT-ACCAATATTTTTTTATAAGAGCTTTAGTTATATATTTGATTAGCATT**

**Kn-0**  **GATCAATAGGTCACCGGTTCAAACCCGATTGGGCCCTACATTTTTTCAGTTT-ACCAATATTTTTTTATAAGAGCTTTAGTTATATATTTGATTAGCATT**

**Cdm-0**  **GATCAATAGGTCACCGGTTCAAACCCGATTGGGCCCTACATTTTTTCAGTTT-ACCAATATTTTTTTATAAGAGCTTTAGTTATATATTTGATTAGCATT**

**Ler**  **GATCAATAGGTCACCGGTTCAAACCCGATTGGGCCCTACATTTTTTCAGTTT-ACCAATATTTTTTTATAAGAGCTTTAGTTATATATTTGATTAGCATT**

**Fja-2-4**  **GATCAATAGGTCACCGGTTCAAATCCGATTGGGCCCT---------------------------------------------------------------**

**Eri-1**  **GATCAATAGGTCACCGGTTCAAACCCGATTGGGCCCTATATTTTTTCAGTTT-ACCAATATTTTTTTATAAGAGCTTTAGTTATATATTTGATTAGCATT**

**Kor3**  **GATCAATAGGTCACCGGTTCAAACCCGATTGGGCCCT---------------------------------------------------------------**

**Kyo**  **GATCAATAGGTCACCGGTTCAAACCCGATTGGGCCCT---------------------------------------------------------------**

**Tur4**  **GATCAATAGGTCACCGGTTCAAACCCGATTGGGCCCTATATTTTTTCAGTTTTACCAATATTTTTTTATAAGAGCTTTAGTTATATATTTGATTAGCATT**

**Ty-1**  **GATCAATAGGTCACCGGTTCGAACCCGATTGGGCCCTATATTTTTTCAGTTT-ACCAATATTTTTTTATAAGAGCTTTAGTTATATATTTGATTAGCATT**

**Fly-2-2**  **GATCAATAGGTCACCGGTTCAAACCCGATTGGGCCCTATATTTTTTCAGTTTTACCAATATTTTTTTATAAGAGCTTTAGTTATATATTTGATTAGCATT**

610 620 630 640 650 660 670 680 690 700

....|....|....|....|....|....|....|....|....|....|....|....|....|....|....|....|....|....|....|....|

**tRNA genes ----------------------------------------------------------------------------------------------------**

**Col-0**  **TAGCGTCAAGTAGTTGGCTAATCTTATATTTCAAGGTTTTAGCTAGCAATTTTTCATCCTGAATAAAACAAATTTTGACCAGTTTTTATATATAATTCTT**

**An-1**  **TAGCGTCAAGTAGTTGACTAATCTTATATTTCAAGGTTTTAGCTAGCAATTTTTTATCCTGAATAAAACAAATTTTGACCAGTTTTTATATATAATTCTT**

**C24**  **TAGCGTCAAGTAGTTGACTAATCTTATATTTCAAGGTTTTAGCTAGCAATTTTTCATCCTGAATAAAACAAATTTTGACCAGTTTTTATATATAATTCTT**

**KBS-Mac-74** **TAGCGTCAAGTAGTTGACTAATCTTATATTTCAAGGTTTTAGCTAGCAATTTTTTATCCTGAATAAAACAAATTTTGACCAGTTTTTATATATAATTCTT**

**Tos82-387**  **TAGCGTCAAGTAGTTGACTAATCTTATATTTCAAGGTTTTAGCTAGCAATTTTTCATCCTGAATAAAACAAATTTTGACCAGTTTTTATATATAATTCTT**

**Cvi**  **TAGCGTCAAGTAGTTGACTAATCTTATATTTCAAGGTTTTAGCTAGCAATTTTTCATCCTGAATAAAACATATTTTGACCAGTTTTTATATATAATTCTT**

**Kn-0**  **TAGCGTCAAGTAGTTGACTAATCTTATATTTCAAGGTTTTAGCTAGCAATTTTTCATCCTGAATAAAACAAATTTTGACCAGTTTTTATATATAATTCTT**

**Cdm-0**  **TAGCGTCAAGTAGTTGACTAATCTTATATTTCAAGGTTTTAGCTAGCGATTTTTCATCCTGAATAAAACAAATTTTGACCAGTTTTTATATATAATTCTT**

**Ler**  **TAGCGTCAAGTAGTTGACTAATCTTATATTTCAAGGTTTTAGCTAGCGATTTTTCATCCTGAATAAAACAAATTTTGACCAGTTTTTATATCTAATTCTT**

**Fja-2-4**  **----------------------------------------------------------------------------------------------------**

**Eri-1**  **TAGCGTCAAGTAGTTGACTAATCTTATATTTCAAGGTTTTAGCTAGCAAATTTTCATCCTGAATAAAACAAATTTTGACCAGTTTTTATATATAATTCTT**

**Kor3**  **----------------------------------------------------------------------------------------------------**

**Kyo**  **----------------------------------------------------------------------------------------------------**

**Tur4**  **TAGCGTCAAGTAGTTGACTAATCTTATATTTCAAGGTTTTAGCTAGCAATTTTTCATCCTGAATAAAACAAATTTTGACCAGTTTTTATATATAATTCTT**

**Ty-1**  **TAGCGTCAAGTAGTTGGCTAATCTTATATTTCAAGGTTTTAGCTAGCAATTTTTCATCCTGAATAAAACAAATTTTGACCAGTTTTTATATATAATTCTT**

**Fly-2-2**  **TAGCGTCAAGTAGTTGACTAATCTTATATTTCAAGGTTTTAGCTAGCAATTTTTCATCCTGAATAAAACAAATTTTGACCAGTTTTTATATATAATTCTT**

710 720 730 740 750 760 770 780 790 800

....|....|....|....|....|....|....|....|....|....|....|....|....|....|....|....|....|....|....|....|

**tRNA genes ----------------------------------------------------------------------------------------------------**

**Col-0**  **TAGCAACTGAATTTTTTTTTTAAAAAAAAATTCATGGTTTAATTTGTTTGTGAATATTTTTTTTAAAATAATTTATGTGATTTCTAAATAAGTAAACAAT**

**An-1**  **TAACAACTGAATTTTTTT-AAAAAAAAAAATTCATGGTTTAATTTGTTTGTGAATATTTTTTTTAAAATAATTTATGTGATTTCTAAATAAGTAAACAAT**

**C24**  **TAGCAACTGAATTTTTTT-AAAAAAAAAAATTCATGGTTTAATTTGTTTGTGAATATTTTTT-AAAAATAATTTATGTGATTTCTAAATAAGTAAACAAT**

**KBS-Mac-74** **TAACAACTGAATTTTTTTT-AAAAAAAAAATTCATGGTTTAATTTGTTTGTGAATATTTTTTTTAAAATAATTTATGTGATTTCTAAATAAGTAAACAAT**

**Tos82-387**  **TAGCAACTGAATTTTTTTTTT-AAAAAAAATTCATGGTTTAATTTGTTTGTGAATATTTTTT-AAAAATAATTTATGTGATTTCTAAATAAGTAAACAAT**

**Cvi**  **TAGCAACTGATTTTTTTTTTTTT-AAAAAATTCATGGTTTAATTTGTTTGTGAATATTTTTTAAAAAATAATTTATGTGATTTCTAAATAAGTAAACAAT**

**Kn-0**  **TAGCAACTGAATTTTTTTTTT--AAAAAAATTCATGGTTTAATTTGTTTGTGAATATTTTTT-AAAAATAATTTATGTGATTTCTAAATAAGTAAACAAT**

**Cdm-0**  **TAGCAACTGAATTTTTTT--AAAAACAAAATTCATGGTTTAATTTGTTTGTGAATATTTTTT-AAAAATAATTTATGTGATTTCTAAATAAGTAAACAAT**

**Ler**  **TAGCAACTGAATTTTTTT--AAAAACAAAATTCATGGTTTAATTTGTTTGTGAATATTTTTT-AAAAATAATTTATGTGATTTCTAAATAAGTAAACAAT**

**Fja-2-4**  **----------------------------------------------------------------------------------------------------**

**Eri-1**  **TAGCAACTGAATTTTTTTT-AAAAAAAAAATTCATGGTTTAATTTGTTTGTGAATATTTTTT-AAAAATAATTTATGTGATTTCTAAATAAGTAAACAAT**

**Kor3**  **----------------------------------------------------------------------------------------------------**

**Kyo**  **----------------------------------------------------------------------------------------------------**

**Tur4**  **TAGCAACTGAATTTTTTTT-AAAAAAAAAATTCATGGTTTAATTTGTTTGTGAATATTTTTTTTTAAATAATTTATGTGATTTCTAAATAAGTAAACAAT**

**Ty-1**  **TAGCAACTGAATTTTTTTTTTAAAAAAAAATTCATGGTTTAATTTGTTTGTGAATATTTTTTTTAAAATAATTTATGTGATTTCTAAATAAGTAAACAAT**

**Fly-2-2**  **TAGCAACTGAATTTTTTTT-AAAAAAAAAATTCATGGTTTAATTTGTTTGTGAATATTTTTTTTTAAATAATTTATGTGATTTCTAAATAAGTAAACAAT**

810 820 830 840 850 860 870 880 890 900

....|....|....|....|....|....|....|....|....|....|....|....|....|....|....|....|....|....|....|....|

**tRNA genes ---------------------------------------------------------------------------------->>>>>>>>>>>>>>>>>>**

**Col-0**  **TGACTTAAATATATTAGATTTCTTAGCAAGAAAAAATTGATGAAATAAATAATTTATAATTTTGAACTTATTAAATCAATAAAGGTCCATAGCTCAGTGG**

**An-1**  **TGACTTAACTATATTAGATTTCTTAG-AAAAAAAAATTGATGAAATAAATAATTTATAATTTTGAACTTATTAAATAAATAAAGGTCCATAGCTCAGTGG**

**C24**  **TGACTTAACTATATTAGATTTCTTAGCAAAAAAAAATTGATGAAATAAATAATTTATAATTTTGAACTTATTAAATCAATAAAGGTCCATAGCTCAGTGG**

**KBS-Mac-74** **TGACTTAACTATATTAGATTTCTTAG-AAAAAAAAATTGATGAAATAAATAATTTATAATTTTGAACTTATTAAATAAATAAAGGTCCATAGCTCAGTGG**

**Tos82-387**  **TGGCTTAACTATATTAGATTTCTTAGCAAAAAAAAATTGATGAAATAAATAATTTATAATTTTCAACTTATTAAATCAATAAAGGTCCATAGCTCAGTGG**

**Cvi**  **TGACTTAACTATATTAGATTTCTTAGCAAAAAAAAATTGATGAAATAAATAATTTATAATTTTGAACTTATTAAACCAATAAAGGTCCATAGCACAGTGG**

**Kn-0**  **TGACTTAACTATATTAGATTTCTTAGCAAAAAAAAATTGATGAAATAAATAATTTATAATTTTAAACTTATTAAATCAATAAAGGTCCATAGCTCAGTGG**

**Cdm-0**  **TGACTTAACTATATTAGATTTCTTAGCAAAAAAAAATTGATGAAATAAATAATTTATAATTTTAAACTTATTAAATCAATAAAGGTCCATAGCTCAGTGG**

**Ler**  **TGACTTAACTATATTAGATTTCTTAGCAAAAAAAAATTGATGAAATAAATAATTTATAATTTTAAACTTATTAAATCAATAAAGGTCCATAGCTCAGTGG**

**Fja-2-4**  **----------------------------------------------------------------------------------------------------**

**Eri-1**  **TGACTTAACTATATTAGATTTCTTAGCAAAAAACAATTTATGAAATAAATAGTTTATAATTTTGAACTTATTAAATCAATAAGGGTCCATAGCTCAGTGG**

**Kor3**  **----------------------------------------------------------------------------------------------------**

**Kyo**  **----------------------------------------------------------------------------------------------------**

**Tur4**  **TGACTTAACTATATTAGATTTCTTAGCAAAAAAAAATTGATGAAATAAATAATTTATAATTTTAAACTTATTAAATCAATAAAGGTCCATAGCTCAGTGG**

**Ty-1**  **TGACTTAAATATATTAGATTTCTTAGCAAGAAAAAATTGATGAAATAAATAATTTATAATTTTGAACTTATTAAATCAATAAAGGTCCATAGCTCAGTGG**

**Fly-2-2**  **TGACTTAACTATATTAGATTTCTTAGCAAAAAAAAATTGATGAAATAAATAATTTATAATTTTAAACTTATTAAATCAATAAAGGTCCATAGCTCAGTGG**

910 920 930 940 950 960 970 980 990 1000

....|....|....|....|....|....|....|....|....|....|....|....|....|....|....|....|....|....|....|....|

**tRNA genes >>>>>>>>>>>>>>>>>>>>>>>>>>>>>>>>>>>>>>>>>>>>>>>>>>>>>>----------------------------------------------**

**Col-0**  **TAGAGCAATTGACTGCAGATCAATAGGTCACCGGTTTGAACCTGGTTGGGCCCTATATTTTTTCAATTTACCAATAATTTTTTAGAAGAGCTTTAGTTAT**

**An-1**  **TAGAGCAATTGACTGCAGATCAATAGGTTACCGGTTCGAACCCGGTTGGGCCCTATATTTTTTCAATTTACCAATAATTTTTTATAAGAGCTTTAGTTAT**

**C24**  **TAGAGCAATTGACTGCAGATCAATAGGTCACCGGTTCGAACCCGGTTGGGCCCTATATTTTTTCAATTTACCAATAATTTTTTATAAGAGCTTTAGTTAT**

**KBS-Mac-74** **TAGAGCAATTGACTGCAGACCAATAGGTTACCGGTTCGAACCCGGTTGGGCCCTATATTTTTTCAATTTACCAATAATTTTTTATAAGAGCTTTAGTTAT**

**Tos82-387**  **TAGAGCAATTGACTGCAGATCAATAGGTCACCGGTTCGAACCCGGTTAGGCCCTATATTTTTTCAATTTACCAATAATTTTTTATAAGAGCTTTAGTTAT**

**Cvi**  **TAGAGCAATTGATTGCAGATCAATAGGTCACCGGTTTGAACCCGGTTGGGCCCT----------------------------------------------**

**Kn-0**  **TAGAGCAATTGACTGCAGAACAATAGGTCACAGGTTCGAACCCGGTTGGGCCCTATATTTTT-CAATTTACCAATAATTTTTTATAAGAGCTTTAGTTAT**

**Cdm-0**  **TAGAGCAATTGACTGCAGAACAATAGGTCACAGGTTCGAACCCGGTTGGGCCCTATATTTTT-CAATTTACCAATAATTTTTTATAAGAGCTTTAGTTAT**

**Ler**  **TAGAGCAATTGACTGCAGAACAATAGGTCACAGGTTCGAACCCGGTTGGGCCCTATATTTTT-CAATTTACCAATAATTTTTTATAAGAGCTTTAGTTAT**

**Fja-2-4**  **------------------------------------------------------ACATTTTTTCAGTTTACCAATATTTTTTTATAAGAGCTTTAGTTAT**

**Eri-1**  **TAGAGCAATTGACTGCAGATCAATAGGTCACCGGTTCGAACCCGGTTGGGCCCT----------------------------------------------**

**Kor3**  **------------------------------------------------------ACATTTTTTCAGTTTACCAATATTTTTTTATAAGAGCTTTAGTTAT**

**Kyo**  **------------------------------------------------------ACATTTTTTCAGTTTACCAATATTTTTTTATAAGAGCTTTAGTTAT**

**Tur4**  **TAGAGCAATTGACTGCAGATCAATAGGTCACCGGTTCAAACCCGGTTGGGCCCTATATTTTTTCAATTTACCAATAATTTTTTATAAGAGCTTTAGTTAT**

**Ty-1**  **TAGAGCAATTGACTGCAGATCAATAGGTCACCGGTTTGAACCTGGTTGGGCCCTATATTTGTTCAATTTACCAATAGTTTTTTATAAGAGCTTTAGTTAT**

**Fly-2-2**  **TAGAGCAATTGACTGCAGATCAATAGGTCACCGGTTCAAACCCGGTTGGGCCCTATATTTTTTCAATTTACCAATAATTTTTTATAAGAGCTTTAGTTAT**

1010 1020 1030 1040 1050 1060 1070 1080 1090 1100

....|....|....|....|....|....|....|....|....|....|....|....|....|....|....|....|....|....|....|....|

**tRNA genes ----------------------------------------------------------------------------------------------------**

**Col-0**  **ATATTTGATTAGCATTTAGCATCAAGTAGTTGACTAATCTTATATTTCAACGTTTTAGCTAGCAATTTTTCATCTTGAATAAAACAAATTTTGATCAGTT**

**An-1**  **ATATTTGATTAGCATTTAGCATCAAGTAGTTGACTAATCTTATATTTCAACGTTTTAGCTACCAATTTTTCATCTTGAATAAAACAAATTTTGACCAGTT**

**C24**  **ATATTTGATTAGCATTTAGCATCAAGTAGTTGACTAATCTTATATTTCAACGTTTTAGGTAGCAATTTTTCATCTTGAATAAAACAAATTTTGACCAGTT**

**KBS-Mac-74** **ATATTTGATTAGCATTTAGCATCAAGTAGTTGACTAATCTTATATTTCAACGTTTTAGCTAGCAATTTTTCATCTTGAATAAAACAAATTTTGACCAGTT**

**Tos82-387**  **ATATTTGATTAGCATTTAGCATCAAGTAGTTGACTAATCTTATATTTCAACGTTTTAGCTAGCAATTTTTCATCTTGAATAAAACAAATTTTGACTAGTT**

**Cvi**  **----------------------------------------------------------------------------------------------------**

**Kn-0**  **ATATTTGATTAGCATTTAGCATCAAGTAGTTGACTAATCTTATATTTCAACGTTTTAGCTAGCAATTTTTCATCTTGAATAAAACAAATTTTGACCAGTT**

**Cdm-0**  **ATATTTGATTAACATTTAGCATCAAGTAGTTGACTAATCTTATATTTCAACGTTTTAGCTAGCAATTTTTCATCTTGAATAAAACAAATTTTGACCAGTT**

**Ler**  **ATATTTGATTAACATTTAGCATCAAGTAGTTGACTAATCTTATATTTCAACGTTTTAGCTAGCAATTTTTCATCTTGAATAAAACAAATTTTGACCAGTT**

**Fja-2-4**  **ATATTTGATTAGCATTTAGCGTCAAGTAGTTGACTAATCTTATATTTCAAGGTTTTAGCTAGCAATTTTTCATCCTGAATAAAACAAATTTTGACCAGTT**

**Eri-1**  **----------------------------------------------------------------------------------------------------**

**Kor3**  **ATATTTGATTAGCATTTAGCGTCAAGTAGTTGACTAATCTTATATTTCAACGTTTTAGCTAGCAATTTTTCATCTTGAATAAAACAAATTTTGACCAGTT**

**Kyo**  **ATATTTGATTAGCATTTAGCGTCAAGTAGTTGACTAATCTTATATTTCAACGTTTTAGCTAGCAATTTTTCATCTTGAATAAAACAAATTTTGACCAGTT**

**Tur4**  **ATATTTGATTAGCATTTAGCATCAAGTAGTTGACTAATCTTATATTTCAACGTTTTAGCTAGCAATTTTTCATCTTGAATAAAACAAATTTTGACCAGTT**

**Ty-1**  **ATATTTGATTAGCATTTAGCATCAAGTAGTTGAATAATCTTATATTTCAACGTTTTAGCTAGCAATTTTTCATCTTGAATAAAACAAATTTTGACCAGTT**

**Fly-2-2**  **ATATTTGATTAGCATTTAGCATCAAGTAGTTGACTAATCTTATATTTCAACGTTTTAGCTAGCAATTTTTCATCTTGAATAAAACAAATTTTGACCAGTT**

1110 1120 1130 1140 1150 1160 1170 1180 1190 1200

....|....|....|....|....|....|....|....|....|....|....|....|....|....|....|....|....|....|....|....|

**tRNA genes ----------------------------------------------------------------------------------------------------**

**Col-0**  **TTTGTATATAATTCTTTAGTAACTGAATTTTTTTTAAAAAAAA-TTCATGGTTTAATTTGTTTGTGAATTTTGTTTTTTAAATAATTTATGTGATTTCTA**

**An-1**  **TTTGTATATAATTCTTTAGTAACTGAATTTTTTTTAAAAACAA-TTCATGGTTTAATTTGTTTGTGAATTTTGTTTTTTAAATAATTTATATGATTTCTA**

**C24**  **TTTGTATATAATTCTTTAGTAACTGA-TTTTTAAAAAAAAAAA-TTCATGGTTTAATTTGTTTGTGAATTTTGTTTTTTAAATCATTTATGTGATTTTTA**

**KBS-Mac-74** **TTTGTATATAATTCTTTAGTAACTGAATTTTTTTTAAAAACAA-TTCATGGTTTAATTTGTTTGTGAATTTTGTTTTTTAAATAATTTATATGATTTCTA**

**Tos82-387**  **TTTGTATATAATTCTTTAGTAACTGATTTTTTTTAAAAAAAAAATTCATGGTTTAATTTGTTTGTGAATTTTGTTTTTTAAATAATTTATGTGATTTTTA**

**Cvi**  **----------------------------------------------------------------------------------------------------**

**Kn-0**  **TTTGTATATAATTCTTTAGTAACTGATTTTTTTTAAAAAAAAA-TTCATGGTTTAATTTGTTTGTGAATTTTTTTTTATAAATAATTTATGTGATTTCTA**

**Cdm-0**  **TTTGTATATAATTCTTTAGTAACTGATTTTTTTTTAAAAAAAA-TTCATGGTTTAATTTGTTTATGAATTTTTTTTTATAAATAATTTATGTGATTTCTT**

**Ler**  **TTTGTATATAATTCTTTAGTAACTGATTTTTTTTAAAAAAAAA-TTCATGGTTTAATTTGTTTATGAATTTTTTTTTATAAATAATTTATGTGATTTCTT**

**Fja-2-4**  **TTTATATATAATTCTTTAGCAACTGAATTTTTTTTTTAAAAAAATTCATGGTTTAATTTGTTTGTGAATATTTTTTAA-AAATAATTTATGTGATTTCTA**

**Eri-1**  **----------------------------------------------------------------------------------------------------**

**Kor3**  **TTTGTATATAATTCTTTAGTAACTGATTTTTTTTAAAAAAAAA-TTCATGGTTTAATTTGTTTGTGAATTTTTTTTTATAAATAATTTATGTGATTTCTA**

**Kyo**  **TTTGTATATAATTCTTTAGTAACTGATTTTTTTTAAAAAAAAA-TTCATGGTTTAATTTGTTTGTGAATTTTTTTTTATAAATAATTTATGTGATTTCTA**

**Tur4**  **TTTGTATATAATTCTTTAGTAACTGAATTTTTTTTAAAAAAAA-TTCATGGTTTAATTTGTTTGTGAATTTTGTTTTTTAAATAATTTATGTGATTTCTA**

**Ty-1**  **TTTGTATATAATTCTTTAGTAACTGAATTTTTTTAAAAAAAAA-ATCATGGTTTAATTTGTTTGTGAATTTTGTTTTTTAAATAATTTATGTGATTTCTA**

**Fly-2-2**  **TTTGTATATAATTCTTTAGTAACTGAATTTTTTTTAAAAAAAA-TTCATGGTTTAATTTGTTTGTGAATTTTGTTTTTTAAATAATTTATGTGATTTCTA**

1210 1220 1230 1240 1250 1260 1270 1280 1290 1300

....|....|....|....|....|....|....|....|....|....|....|....|....|....|....|....|....|....|....|....|

**tRNA genes ------------------------------------------------------------------------------------------------->>>**

**Col-0**  **AATAAGTAAACAATTGACTTAACTATATTAGATTTCTTAGCAAAAAAA-AATTGATGAAATAAATAATTTACAATTTTGAACTTATTAAATCAATAAGGG**

**An-1**  **AATAAGTAAACAATTGACTTAACTATATTAGATTTCTTAGCAAAAAAAAAATTGATGAAATAAATAATTTACAATTTTGAACTTATTAAATCAATAAGGG**

**C24**  **AATAAGTAAACAATTGACTTAACTATATTAGATTTCTTAGCAAAAAAA-AATTGATGAAATAAATAATTTACAATTTTGAACTTATTAAATCAATAAGGG**

**KBS-Mac-74** **AATAAGTAAACAATTGACTTAACTATATTAGATTTCTTAGAAAAAAAA-AATTGATGAAATAAATAATTTACAATTTTGAACTTATTAAATCAATAAGGG**

**Tos82-387**  **AATAAGTAAACAATTGACTTAACTATATTAGATTTCTTAGCAAAAAAA-AATTGATGAAATAAATAATTTACAATTTTGAACTTATTAAATCAATAAGGG**

**Cvi**  **----------------------------------------------------------------------------------------------------**

**Kn-0**  **AATAAGTAAACAATTGACTTAACTATATTAGATTTCTTAGCAAAAAA-CAATTTATGAAATAAATAGTTTATAATTTTGAACTTATTAAATCAATAAGGG**

**Cdm-0**  **AATAAGTAAACAATTGACTTAACTATATTAGATTTCTTAGCAAAAAA-CAATTTATGAAATAAATAGTTTATAATTTTGAACTTATTAAATCAATAAGGG**

**Ler**  **AATAAGTAAACAATTGACTTAACTATATTAGATTTCTTAGCAAAAAA-CAATTTATGAAATAAATAGTTTATAATTTTGAACTTATTAAATCAATAAGGG**

**Fja-2-4**  **AATAAGTAAACAATTGACTTAACTATATTAGATTTCTTAGCAAAAAA-AAATTGATGAAATAAATAATTTATAATTTTAAACTTATTAAATCAATAAAGG**

**Eri-1**  **----------------------------------------------------------------------------------------------------**

**Kor3**  **AATAAGTAAACAATTGACTTAACTATATTAGATTTCTTAGCAAAAAA-CAATTTATGAAATAAATAGTTTATAATTTTGAACTTATTAAATCAATAAGGG**

**Kyo**  **AATAAGTAAACAATTGACTTAACTATATTAGATTTCTTAGCAAAAAA-CAATTTATGAAATAAATAGTTTATAATTTTGAACTTATTAAATCAATAAGGG**

**Tur4**  **AATAAGTAAACAATTGACTTAACTATATTAGATTTCTTAGCAAAAAA--AATTGATGAAATAAATAATTTACAATTTTGAACTTATTAAATCAATAAGGG**

**Ty-1**  **AATAAGTAAACAATTGACTTAACTATATTAGATTTCTTAGCAAAAAAA-AATTGATGAAATAAATAATTTACAATTTTGAACTTATTAAATCAATAAGGG**

**Fly-2-2**  **AATAAGTAAACAATTGACTTAACTATATTAGATTTCTTAGCAAAAAA--AATTGATGAAATAAATAATTTACAATTTTGAACTTATTAAATCAATAAGGG**

1310 1320 1330 1340 1350 1360 1370 1380 1390 1400

....|....|....|....|....|....|....|....|....|....|....|....|....|....|....|....|....|....|....|....|

**tRNA genes >>>>>>>>>>>>>>>>>>>>>>>>>>>>>>>>>>>>>>>>>>>>>>>>>>>>>>>>>>>>>>>>>>>>>-------------------------------**

**Col-0**  **TCCATAGCTCAGTGATAGAGCAATTGACTGCAGATCAATAGGTCACCGGTTCGAACCCGGTTGGGCCCTATATGTTTTCAGTTTACCAATAATTTTTTAT**

**An-1**  **TCCATAGCTCAGTGATAGAGCAATTGACTGCAGATCAATAGGTCACCGGTTCGAACCCGGTTGGGCCCTATATGTTTTCAGTTTACCAATAATTTTTTAT**

**C24**  **TTCATAGCTCAGTGATAGAGCAATTGACTGCAGATCAATAGGTCACCGGTTCGAACCCGGTTGGGCCCTATATGTTTTCAGTTTACCAATAATTTTTTTT**

**KBS-Mac-74** **TCCATAGCTCAGTGATAGAGCAATTGACTGCAGATCAATAGGTCACCGGTTCGAACCCGGTTGGGCCCTATATGTTTTCAGTTTACCAATAATTTTTTAT**

**Tos82-387**  **TTCATAGCTCAGTGATAGAGCAATTGACTGCAGATCAATAGGTCACCGGTTCGAACCCGGTTGGGCCCTATATGTTTTCAGTTTACCAATAATTTTTTAT**

**Cvi**  **---------------------------------------------------------------------ATATGTTTTCAGTTTACCAATAATTTTTTGT**

**Kn-0**  **TCCATAGCTCAGTGGTAGAGCAATTGACTGCAGATCAATAGGTCACCGGTTCGAACCCGGTTGGGCCCT-------------------------------**

**Cdm-0**  **TCCATAGCTCAGTGGTAGAGCAATTGACTGCAGATCAATAGGTCACCGGTTCGAACCCGGTTGGGCCCT-------------------------------**

**Ler**  **TCTATAGCTCAGTGGTAGAGCAATTGACTGCAGATCAATAGGTCACCGGTTCGAACCCGGTTGGGCCCT-------------------------------**

**Fja-2-4**  **TCCATAGCTCAGTGGTAGAGCAATTGACTGCAGAACAATAGGTCACAGGTTCGAACCCGGTTGGGCCCTATATTTTTCAATTTACCAATAATTTTTTATA**

**Eri-1**  **----------------------------------------------------------------------------------------------------**

**Kor3**  **TCCATAGCTCAGTGGTAGAGCAATTGACTGCAGATCAATAGGTCACCGGTTCGAACCCGGTTGGGCCCT-------------------------------**

**Kyo**  **TCCATAGCTCAGTGGTAGAGCAATTGACTGCAGATCAATAGGTCACCGGTTCGAACCCGGTTGGGCCCT-------------------------------**

**Tur4**  **TCCATAGCTCAGTGATAGAGCAATTGACTGCAGATCAATAGGTCACCGGTTCGAACCCGGTTGGGCCCTATATGTTTTCAGTTTACCAATAATTTTTTAT**

**Ty-1**  **TCCATAGCTCAGTGATAGAGCAATTGACTGCAGATCAATAGGTCACCGGTTCGAACCCGGTTGGGCCCTATATGTTTTCAGTTTATCAATAATTTTTTAT**

**Fly-2-2**  **TCCATAGCTCAGTGATAGAGCAATTGACTGCAGATCAATAGGTCACCGGTTCGAACCCGGTTGGGCCCTATATGTTTTCAGTTTACCAATAATTTTTTAT**

1410 1420 1430 1440 1450 1460 1470 1480 1490 1500

....|....|....|....|....|....|....|....|....|....|....|....|....|....|....|....|....|....|....|....|

**tRNA genes ----------------------------------------------------------------------------------------------------**

**Col-0**  **AAGAGTTTTAGTTATATATTTGATTAGCATTTAGCGTCAAGTAGTTCACTAATCTTATATTTCAAGGTTTTAGCTAGCAATTTTTCATCTTGAATAAAAC**

**An-1**  **AAGAGTTTTAGTTATATATTTGATTAGCATTTAGGGTCAAGTAGTTCACTAATCTTATACTTCAAGGTTTTAGCTAGCAATTTT-CATCTTGAATAAAAC**

**C24**  **AAGAGTTTTAGTTATATATTTGATTAGCATTTAGCGTCAAGTAGTTCACTAATCTTATATTTCAAGGTTTTAGCTAGCAATTTTTCATCTTGAATAAAAC**

**KBS-Mac-74** **AAGAGTTTTAGTTATATATTTGATTAGCATTTAGGGTCAAGTAGTTCACTAATCTTATACTTCAAGGTTTTAGCTAGCAATTTT-CATCTTGAATAAAAC**

**Tos82-387**  **AAGAGTTTTAGTTATATATTTGATTAGCATTTAGCGTCAAGTAGTTCACTAATCTTATATTTCAAGGTTTTAGCTAGCAATTTTTCATCTTGAATAAAAC**

**Cvi**  **AAGAGTTTTAGTTATATATTTGATTAGCATTTAGCGTCAAGTAGTTCACTAATCTTATATTTCAAGGTTTTAGCTAGCAATTTTTCATCTTGAATAAAAC**

**Kn-0**  **----------------------------------------------------------------------------------------------------**

**Cdm-0**  **----------------------------------------------------------------------------------------------------**

**Ler**  **----------------------------------------------------------------------------------------------------**

**Fja-2-4**  **AGAGCTTTAGTTATATATTTGATTAGCATTTAGCATCAAGTAGTTGACTAATCTTATATTT-CAACGTTTTAGCTAGCAATTTTTCATCTTGAATAAAAC**

**Eri-1**  **----------------------------------------------------------------------------------------------------**

**Kor3**  **----------------------------------------------------------------------------------------------------**

**Kyo**  **----------------------------------------------------------------------------------------------------**

**Tur4**  **AAGAGTTTTAGTTATATATTTGATTAGCATTTAGCGTCAAGTAGTTCACTAATCTTATATTTCAAGGTTTTAGCTAGCAATTTTTCATTTTGAATAAAAT**

**Ty-1**  **AAGAATTTTAGTTATATATTTGATTAGCATTTAGCGTCAAGTAGTTCACTAATCTTATATTTCAAGGTTTTAGCTAGCAATTTTTCATCTTGAATAAAAC**

**Fly-2-2**  **AAGAGTTTTAGTTATATATTTGATTAGCATTTAGCGTCAAGTAGTTCACTAATCTTATATTTCAAGGTTTTAGCTAGCAATTTTTCATTTTGAATAAAAT**

1510 1520 1530 1540 1550 1560 1570 1580 1590 1600

....|....|....|....|....|....|....|....|....|....|....|....|....|....|....|....|....|....|....|....|

**tRNA genes ----------------------------------------------------------------------------------------------------**

**Col-0**  **AAATTTTGACCTGTTTTTGCATATAATTCTTTAACAACTGAAATTTTTTTAAAAAAC-TTTCATGGTTTAATTTGTTTGTGAATTTTTTTTTATAAATAA**

**An-1**  **AAATTTTGACCTGTTTTTGCATATAATTCTTTAACAACTGAATTTTTTTTTAAAAAC-TTTCATGGTTTAATTTGTTTGTGAATTTTTTTTTATAAATAA**

**C24**  **ACATTTTGACCTGTTTTTGCATATAATTCTTTAACAACTGAATTTTTTTAAAAAAAC-TTTCATGGTTTAATTTGTTTGTGAATTTTTTTTTATAAATAA**

**KBS-Mac-74** **AAATTTTGACCTGTTTTTGCATATAATTCTTTAACAACTGAATTTTTTTTTAAAAAC-TTTCATGGTTTAATTTGTTTGTGAATTTTTTTTTATAAATAA**

**Tos82-387**  **AAATTTTGACCTGTTTTTGCATATAATTCTTTAACAACTGATTTTTTTTTAAAAAAC-TTTCATGGTTTAATTTGTTTGTGAATTTTTTTTTATAAATAA**

**Cvi**  **AAATTTTGACCTGTTTTTGCATATAATTCTTTAACAACTGAATTTTTTTTTAAAAAC-TTTCATGGTTTAATTTGTTTGTGAATTTTTTTTTATAAATAA**

**Kn-0**  **----------------------------------------------------------------------------------------------------**

**Cdm-0**  **----------------------------------------------------------------------------------------------------**

**Ler**  **----------------------------------------------------------------------------------------------------**

**Fja-2-4**  **AAATTTTGACCAGTTTTTGTATATAATTCTTTAGTAACTGATTTTTTTTAAAAAAAAA-TTCATGGTTTAATTTGTTTGTGAATTTTTTTTTATAAATAA**

**Eri-1**  **----------------------------------------------------------------------------------------------------**

**Kor3**  **----------------------------------------------------------------------------------------------------**

**Kyo**  **----------------------------------------------------------------------------------------------------**

**Tur4**  **AAATTTTGACCTGTTTTTGCATATAATTCTTTAACAACTGAATTTTTTTTTAAAAAC-TTTCATGGTTTAATTTGTTTGTGAATTTTTTTTTATAAATAA**

**Ty-1**  **AAATTTTGACCTGTTTTTGCATATAGTTCTTTAATAACTGAAATTTTTTTAAAAAAC-TTTCATGGTTTAATTTGTTTGTGAATTTTATTTTATAAATAA**

**Fly-2-2**  **AAATTTTGACCTGTTTTTGCATATAATTCTTTAACAACTGAATTTTTTTTAAAAAAC-TTTCATGGTTTAATTTGTTTGTGAATTTTTTTTTATAAATAA**

1610 1620 1630 1640 1650 1660 1670 1680 1690 1700

....|....|....|....|....|....|....|....|....|....|....|....|....|....|....|....|....|....|....|....|

**tRNA genes ----------------------------------------------------------------------------------------------------**

**Col-0**  **TTTATGTGATTTCTAAATAAGTAAACAATTGACTTAACTATATTAGATTTCTTAGCAAAAAATAATTCATGAAATACATAGTTTATAATTTTGAACTTAT**

**An-1**  **TTTATGTGATTTCTAAATAAGTAAACAATTGACTTAACTATATTAGATTTCTTAGCAAAAAACAATTTATGAAATAAATAGTTTATAATTTTGAACTTAT**

**C24**  **TTTATGTGATTTCTAAATAAGTAAACAATTGACTTAACTATATTAGATTTCTTAGCAAAAAACAATTTATGAAATAAATAGTTTATAATTTTGAACTTAT**

**KBS-Mac-74** **TTTATGTGATTTCTAAATAAGTAAACAATTGACTTAACTATATTAGATTTCTTAGCAAAAAACAATTTATGAAATAAATAGTTTATAATTTTGAACTTAT**

**Tos82-387**  **TTTATGTGATTTCTAAATAAGTAAACAATTGACTTAACTATATTAGATTTCTTAGCAAAAAACAATTTATGAAATAAATAGTTTATAATTTTGAACTTAT**

**Cvi**  **TTTATGTGATTTCTAAATAAGTAAACAATTGACTTAACTATATTAAATTTCTTAGCAAAAAATAATTCATGAAATAAATAGTTTATAATTTTGAACTTAT**

**Kn-0**  **----------------------------------------------------------------------------------------------------**

**Cdm-0**  **----------------------------------------------------------------------------------------------------**

**Ler**  **----------------------------------------------------------------------------------------------------**

**Fja-2-4**  **TTTATGTGATTTCTAAATAAGTAAACAATTGACTTAACTATATTAGATTTCTTAGCAAAAAACAATTTATGAAATAAATAGTTTATAATTTTGAACTTAT**

**Eri-1**  **----------------------------------------------------------------------------------------------------**

**Kor3**  **----------------------------------------------------------------------------------------------------**

**Kyo**  **----------------------------------------------------------------------------------------------------**

**Tur4**  **TTTATGTGATTTCTAAATAAGTAAACAATTGACTTAACTATATTAGATTTCTTAGCAAAAAATAATTCATGAAATAAATAGTTTATAATTTTGAACTTAT**

**Ty-1**  **TTTATGTGATTTCTAAATAAGTAAACAATTGACTTAACTATATTAGATTTCTTAGCAAAAAATAATTCATGAAATAAATAGTTTATAATTTTGAACTTAT**

**Fly-2-2**  **TTTATGTGATTTCTAAATAAGTAAACAATTGACTTAACTATATTAGATTTCTTAGCAAAAAATAATTCATGAAATAAATAGTTTATAATTTTGAACTTAT**

1710 1720 1730 1740 1750 1760 1770 1780 1790 1800

....|....|....|....|....|....|....|....|....|....|....|....|....|....|....|....|....|....|....|....|

**tRNA genes ----------->>>>>>>>>>>>>>>>>>>>>>>>>>>>>>>>>>>>>>>>>>>>>>>>>>>>>>>>>>>>>>>>>>>>>>>>-----------------**

**Col-0**  **TAAATCAATAAGGGTTCATAGCTCAGTGGTAGAGCAATTGACTGCAGATCAATAGGTCACCGGTTCGAACCCGGTTGGGCCCT-----------------**

**An-1**  **TAAATCAATAAGGGTCCATAGCTCAGTGGTAGAGCAATTGACTGCAGATCAATAGGTCACCGGTTCGAACCCGGTTGGGCCCTATATTTTTTCAGTTTAC**

**C24**  **TAAATCAATAAGGGTCCATAGCTCAGTGGTAGAGCAATTGACTGCAGATCAATAGGTCACCGGTTTGAACCCGGTTGGGCCCTATATTTTTTCAGTTTAC**

**KBS-Mac-74** **TAAATCAATAAGGGTCCATAGCTCAGTGGTAGAGCAATTGACTGCAGATCAATAGGTCACCGGTTCGAACCCGGTTGGGCCCTATATTTTTTCAGTTTAC**

**Tos82-387**  **TAAATCAATAAGGGTCCATAGCTCAGTGGTAGAGCAATTAACTGCAGATCAATAGGTCACCGGTTCGAACCCGGTTGGGCCCTATATTTTTTCAGTTTAC**

**Cvi**  **TAAATCAATAAGGGTCCATAGCTCAGTGGTAGAGCAATTGACTGCAGATCAATAGGTCACCGGTTCGAACCCGGTTGGGCCCTATATTTTTTCAGTTTAC**

**Kn-0**  **-----------------------------------------------------------------------------------ATATTTTTTCAGTTTAC**

**Cdm-0**  **-----------------------------------------------------------------------------------ATATTTTTTCAGTTTAC**

**Ler**  **-----------------------------------------------------------------------------------ATATTTTTTCAGTTTAC**

**Fja-2-4**  **TAAATCAATAAGGGTCCATAGCTCAGTGGTAGAGCAATTGACTGCAGATCAATAGGTCACCGGTTCGAACCCGGTTGGGCCCTATATTTTTTCAGTTTAC**

**Eri-1**  **-----------------------------------------------------------------------------------ATATTTTTTCAGTTTAC**

**Kor3**  **-----------------------------------------------------------------------------------ATATTTTTTCAGTTTAC**

**Kyo**  **-----------------------------------------------------------------------------------ATATTTTTTCAGTTTAC**

**Tur4**  **TAAATCAATAAGGGTCCATAGCTCAGTGGTAGAGCAATTGACTGCAGATCAATAGGTCACCGGTTCGAACCCGGTTGGGCCCT-----------------**

**Ty-1**  **TAAATCAATAAGGGTTCATAGCTCAGTAGTAGAGCAATTGACTGCAGATCAATAGGTCACCGGTTCGAACCCGGTTGGGCCCT-----------------**

**Fly-2-2**  **TAAATCAATAAGGGTCCATAGCTCAGTGGTAGAGCAATTGACTGCAGATCAATAGGTCACCGGTTCGAACCCGGTTGGGCCCT-----------------**

1810 1820 1830 1840 1850 1860 1870 1880 1890 1900

....|....|....|....|....|....|....|....|....|....|....|....|....|....|....|....|....|....|....|....|

**tRNA genes ----------------------------------------------------------------------------------------------------**

**Col-0**  **----------------------------------------------------------------------------------------------------**

**An-1**  **CAATAATTTTTAATAAGAGCTTTAGTTATATGTTTGATTAGCATTTAGCGTCAAGTAGTTGACTAATCTTATATTTCAAGGTTTTAGCTAGTAATTTTTC**

**C24**  **CAATAATTTTTAATAAGAGCTTTAGTTATATATTTGATTAGCATTTAGCGTCAAGTAGTTGACTAATCTTATATTTCAAGGTTTTAGTTAGTAATTTTTC**

**KBS-Mac-74** **CAATAATTTTTAATAAGAGCTTTAGTTATATGTTTGATTAGCATTTAGCGTCAAGTAGTTGACTAATCTTATATTTCAAGGTTTTAGCTAGTAATTTTTC**

**Tos82-387**  **CAATAATTTTTAATAAGAGCTTTAGTTATATATTTGATTAGCATTTAGCGTCAAGTAGTTGACTAATCTTATATTTCAAGGTTTTAGTTAGTAATTTTTC**

**Cvi**  **CAATAATTTTTAATAAGAGCTTTAGTTATATATTTGATTAGCATTTAGCGTCAAGTAGTTGACTAATCTTATATTTCAAGGTTTTAGCTAGTAATTTTTC**

**Kn-0**  **CAATAATTTTTAATAAGAGCTTTAGTTATATATTTGATTAGCATTTAGCGTCAAGTAGTTGACTAATCTTATATTTCAAGGTTTTAGTTAGTAATTTTTC**

**Cdm-0**  **CAATAATTTTTAATAAGAGCTTTAGTTATATATTTGATTAGCATTTAGCGTCAAGTAGTTGACTAATCTTATATTTCAAGGTTTTAGTTAGTAATTTTTC**

**Ler**  **CAATAATTTTTAATAAGAGCTTTAGTTATATATTTGATTAGCATTTAGCGTCAAGTAGTTGACTAATCTTATATTTCAAGGTTTTAGTTAGTAATTTTTC**

**Fja-2-4**  **CAATAATTTTTAATAAGAGCTTTAGTTATATATTTGATTAGCATTTAGCGTCAAGTAGTTGACTAATCTTATATTTCAAGGTTTTAGTTAGTAATTTTTC**

**Eri-1**  **CAATAATTTTTAATAAGAGCTTTAGTTATATATTTGATTAGCATTTAGCGTCAAGTAGTTGACTAATCTTATATTTCAAGGTTTTAGTTAGTAATTTTTC**

**Kor3**  **CAATAATTTTTAATAAGAGCTTTAGTTATATATTTGATTAGCATTTAGCGTCAAGTAGTTGACTAATCTTATATTTCAAGGTTTTAGTTAGTAATTTTTC**

**Kyo**  **CAATAATTTTTAATAAGAGCTTTAGTTATATATTTGATTAGCATTTAGCGTCAAGTAGTTGACTAATCTTATATTTCAAGGTTTTAGTTAGTAATTTTTC**

**Tur4**  **----------------------------------------------------------------------------------------------------**

**Ty-1**  **----------------------------------------------------------------------------------------------------**

**Fly-2-2**  **----------------------------------------------------------------------------------------------------**

1910 1920 1930 1940 1950 1960 1970 1980 1990 2000

....|....|....|....|....|....|....|....|....|....|....|....|....|....|....|....|....|....|....|....|

**tRNA genes ----------------------------------------------------------------------------------------------------**

**Col-0**  **----------------------------------------------------------------------------------------------------**

**An-1**  **ATCTTGAATAAAATAAATTTTGACAAGTTTTT-GTATATAATTCTTGAGCAACTGAAAATATTTTTTT-AAAATTTCATGGTTTAATTTGTTTGTTATTT**

**C24**  **ATCTTGAATAAAATAAATTTTGACAAGTTTTT-GTATATAATTCTTGAGCAACTGAAAATATTTTTT--AAAATTTCATGGTTTAATTTGTTTGTTATTT**

**KBS-Mac-74** **ATCTTGAATAAAATAAATTTTGACAAGTTTTT-GTATATAATTCTTGAGCAACTGAAAATATTTTTTT-AAAATTTCATGGTTTAATTTGTTTGTTATTT**

**Tos82-387**  **ATCTTGAATAAAATAAATTTTGACAAGTTTTT-GTATATAATTCTTGAGCAACTGAAAATATTTTTTT-AAAATTTCATGGTTTAATTTGTTTGTTATTT**

**Cvi**  **AT---GAATAAAATAAATTTTGACAAGTTTTTTGTATATAATTCTTGAGCAACTGAAAATATTTTTTT-AAAATTTCATGGTTTAATTTGTTTGTTATTT**

**Kn-0**  **ATCTTGAATAAAATAAATTTTGACAAGTTTTT-GTATATAATTCTTGAGCAACTGAAAATATTTTTTTTAAAATTTCATGGTTTAGTTTGTTTGTTATTT**

**Cdm-0**  **ATCTTGAATAAAATAAATTTTGACAAGTTTTT-GTATATAATTCTTGAGCAACTGAAAATATTTTTTT-AAAATTTCATGGTTTAGTTTGTTTGTTATTT**

**Ler**  **ATCTTGAATAAAATAAATTTTGACAAGTTTTT-GTATATAATTCTTGAGCAACTGAAAATATTTTTTT-AAAATTTCATGGTTTAGTTTGTTTGTTATTT**

**Fja-2-4**  **ATCTTGAATAAAATAAATTTTGACAAGTTTTT-GTATATAATTCTTGAGCAACTGAAAATATTTTTTTTAAAATTTCATGGTTTAGTTTGTTTGTTATTT**

**Eri-1**  **ATCTTGAATAAAATAAATTTTGACAAGTTTTT-GTATATAATTCTTGAGCAACTGAAAATATTTTTTT-AAAATTTCATGGTTTAATTTGTTTGTTATTT**

**Kor3**  **ATCTTGAATAAAATAAATTTTGACAAGTTTTT-GTATATAATTCTTGAGCAACTGAAAATATTTTTTT-AAAATTTCATGGTTTAGTTTGTTTGTTATTT**

**Kyo**  **ATCTTGAATAAAATAAATTTTGACAAGTTTTT-GTATATAATTCTTGAGCAACTGAAAATATTTTTTT-AAAATTTCATGGTTTAGTTTGTTTGTTATTT**

**Tur4**  **----------------------------------------------------------------------------------------------------**

**Ty-1**  **----------------------------------------------------------------------------------------------------**

**Fly-2-2**  **----------------------------------------------------------------------------------------------------**

2010 2020 2030 2040 2050 2060 2070 2080 2090 2100

....|....|....|....|....|....|....|....|....|....|....|....|....|....|....|....|....|....|....|....|

**tRNA genes ----------------------------------------------------------------------------------------------------**

**Col-0**  **----------------------------------------------------------------------------------------------------**

**An-1**  **GTTTTATTAAATAATTATGTGATTTCTAAATAAGTAAATAATTGACTATATTAGATTTCTTAGCAAAAAAACAATTGACGAAATAAATAATTTACAATTT**

**C24**  **GTTTTATGAAATAATTATGTGATTTCTAAATAAGTAAATAATTGACTATATTAGATTTCTTAGCAAAAAAACAATTGACGAAATAAATAATTTACAATTT**

**KBS-Mac-74** **GTTTTATTAAATAATTATGTGATTTCTAAATAAGTAAATAATTGACTATATTAGATTTCTTAGCAAAAAAACAATTGACGAAATAAATAATTTACAATTT**

**Tos82-387**  **GTTTTATGAAATAATTATGTGATTTCTAAATAAGTAAATAATTGACTATATTAGATTTCTTAGCAAAAAAACAATTGACGAAATAAATAATTTACAATTT**

**Cvi**  **GTTTTATTAAATAATTATGTGATTTCTAAATAAGTAAATAATTGACTATATTAGAT--------------------------------------------**

**Kn-0**  **GTTTTATGAAATAATTATGTGATTTCTAAATAAGTAAATAATTGACTATATTAGATTTCTTAGCAAAAAAACAATTGACGAAATAAATAATTTACAATTT**

**Cdm-0**  **GTTTTATGAAATAATTATGTGATTTCTAAATAAGTAAATAATTGACTATATTAGATTTTTTAGCAAAAAAACAATTGACGAAATAAATAATTTACAATTT**

**Ler**  **GTTTTATGAAATAATTATGTGATTTCTAAATAAGTAAATAATTGACTATATTAGATTTTTTAGCAAAAAAACAATTGACGAAATAAATAATTTACAATTT**

**Fja-2-4**  **GTTTTATGAAATAATTATGTGATTTCTAAATAAGTAAATAATTGACTATATTAGATTTCTTAGCAAAAAAACAGTTGACGAAATAAATAATTTACAATTT**

**Eri-1**  **GTTTTATGAAATAATTATGTGATTTCTAAATAAGTAAATAATTGACTATATTAGATTTCTTAGCAAAAAAACAATTGACGAAATAAATAATTTACAATTT**

**Kor3**  **GTTTTATGAAATAATTATGTGATTTCTAAATAAGTAAATAATTGACTATATTAGATTTCTTAGCAAAAAAACAATTGACGAAATAAATAATTTACAATTT**

**Kyo**  **GTTTTATGAAATAATTATGTGATTTCTAAATAAGTAAATAATTGACTATATTAGATTTCTTAGCAAAAAAACAATTGACGAAATAAATAATTTACAATTT**

**Tur4**  **----------------------------------------------------------------------------------------------------**

**Ty-1**  **----------------------------------------------------------------------------------------------------**

**Fly-2-2**  **----------------------------------------------------------------------------------------------------**

2110 2120 2130 2140 2150 2160 2170 2180 2190 2200

....|....|....|....|....|....|....|....|....|....|....|....|....|....|....|....|....|....|....|....|

**tRNA genes**  **------------------->>>>>>>>>>>>>>>>>>>>>>>>>>>>>>>>>>>>>>>>>>>>>>>>>>>>>>>>>>>>>>>>>>>>>>>>---------**

**Col-0. ----------------------------------------------------------------------------------------------------**

**An-1**  **GAACTTATTAAAGCAATAAGGGTCCATAGCTCAGTGGTAGAGCAATTGGCTGCAGATCAATAGGTCACTGGTTCGAACCCGGTTGGGCCCTATAT-TTTT**

**C24**  **GAACTTATTAAAGCAATAAGGGTCCATAGTTCAGTGGTAGAGCAATTGACTGCAGATCAATAGGTCACCGGTTCGAATCCGGTTGGGCCTTATAT-TTTT**

**KBS-Mac-74** **GAACTTATTAAAGCAATAAGGGTCCATAGCTCAGTGGTAGAGCAATTGGCTGCAGATCAATAGGTCACTGGTTCGAACCCGGTTGGGCCCTATAT-TTTT**

**Tos82-387**  **GAACTTATTAAAGCAATAAGGGTCCATACCTCAGTGGTAGAGCAATTGACTGCAGATCAATAGGTCACCGGTTCGAACCCGGTTGGGCCCTATATTTTTT**

**Cvi**  **---------------------------------------------------------------------------------------------AT-TTTT**

**Kn-0**  **GAACTTATTAAAGCAATAAGGGTCCATAGCTCAGTGGTAGAGCAATTGACTGCAGATCAATAGGTCACCGGTTCGAATCCGGTTGGGCCCTATAT-TTTT**

**Cdm-0**  **GAACTTATTAAAGCAATAAGGGTCCATAGCTCAGTGGTAGAGCAATTGACTGCAGATCAATAGGTCACCGGTTCGAACCCGGTTGGGCCCTATAT-TTTT**

**Ler**  **GAACTTATTAAAGCAATAAGGGTCCATAGCTCAGTGGTAGAGCAATTGACTGCAGATCAATAGGTCACCGGTTCGAACCCGGTTGGGCCCTATAT-TTTT**

**Fja-2-4**  **GAACTTATTAAAGCAATAAGGGTCCATAGCTCAGTGGTAGAGCAATTGACTGCAGATCAATAGGTCACCGGTTCGAATCCGGTTGGGCCCTATAT-TTTT**

**Eri-1**  **GAACTTATTAAAGCAATA-AGGTCCATAGCTCAGTGGTAGAGCAATTGACTGCAGATCAATAGGTCACCGGTTCGAACCCGGTTGGGCCCTATAT-TTTT**

**Kor3**  **GAACTTATTAAAGCAATAAGGGTCCATAGCTCAGTGGTAGAGCAATTGACTGCAGATCAATAGGTCACCGGTTCGAATCCGATTGGGCCCTATAT-CTTT**

**Kyo**  **GAACTTATTAAAGCAATAAGGGTCCATAGCTCAGTGGTAGAGCAATTGACTGCAGATCAATAGGTCACCGGTTCGAATCCGATTGGGCCCTATAT-CTTT**

**Tur4**  **----------------------------------------------------------------------------------------------------**

**Ty-1**  **----------------------------------------------------------------------------------------------------**

**Fly-2-2**  **----------------------------------------------------------------------------------------------------**

2210 2220 2230 2240 2250 2260 2270 2280 2290 2300

....|....|....|....|....|....|....|....|....|....|....|....|....|....|....|....|....|....|....|....|

**tRNA genes ----------------------------------------------------------------------------------------------------**

**Col-0**  **----------------------------------------------------------------------------------------------------**

**An-1**  **CAGTTTACCAATATTTTTTATAAGAGTTTTATTAATATATTTGATTAACATTTAGCGTCTACTAGTTGACTAATTTTATATTATAAGGTTTTTTCTCGCA**

**C24**  **CAGTTTACCAATATTTTTTATAAGAGTTTTATTAATATATTTGATTAACATTTAGCGTCTACTAGTTGACTAATTTTATATTATAAGGTTTTTTCTCGCA**

**KBS-Mac-74** **CAGTTTACCAATATTTTTTATAAGAGTTTTATTAATATATTTGATTAACATTTAGCGTCTACTAGTTGACTAATTTTATATTATAAGGTTTTTTCTCGCA**

**Tos82-387**  **A-GTTTACCAATATTTTTTATAAGAGTTTTATTAATATATTTGATTAACATTTAGCGTCTACTAGTTGACTAATTTTATATTATAAGGTTTTTTCTCGCA**

**Cvi**  **CAGTTTACCAATATTTTTTATAAGAGTTTTATTAATATATTTGATTAACATTTAGCGTCTATTAGTTGACTAATTTTATATTATAAGGTTTTTTGTCGCA**

**Kn-0**  **CAGTTTACCAATATTTTTTATAAGAGCTTTATTAATATATTTGATTAACATTTAGCGTCTACTAGTTGACTAATTTTATATTATAAAGTTTTTTCTCGCA**

**Cdm-0**  **CAGTTTACCAATATTTTTTATAAGAGTTTTATTAATATATTTGATTAACATTTAGCGTCTACTAGTTGACTAATTTTATATTATAAGGTTTTTTCTCGCA**

**Ler**  **CAGTTTACCAATATTTTTTATAAGAGCTTTATTAATATATTTGATTAACATTTAGCGTCTACTAGTTGACTAATTTTATATTATAAGGTTTTTTCTCGCA**

**Fja-2-4**  **CAGTTTACCAATATTTTTTATAAGAGCTTTATTAATATATTTGATTAACATTTAGCGTCTACTAGTTGACTAATTTTATATTATAAAGTTTTTTCTCGCA**

**Eri-1**  **CAGTTTACCAATATTTTTTATAAGAGTTTTATTAATATATTTGATTAACATTTAGCGTCTACTAGTTGACTAATTTTATATTATAAGGTTTTTTCTCGCA**

**Kor3**  **CAGTTTACCAATATTTTTTATAAGAGCTTTATTAATATATTTGATTAACATTTAGCGTCTACTAGTTGACTAATTTTATATTATAAAGTTTTTTTTCGCA**

**Kyo**  **CAGTTTACCAATATTTTTTATAAGAGCTTTATTAATATATTTGATTAACATTTAGCGTCTACTAGTTGACTAATTTTATATTATAAAGTTTTTTTTCGCA**

**Tur4**  **----------------------------------------------------------------------------------------------------**

**Ty-1**  **----------------------------------------------------------------------------------------------------**

**Fly-2-2**  **----------------------------------------------------------------------------------------------------**

2310 2320 2330 2340 2350 2360 2370 2380 2390 2400

....|....|....|....|....|....|....|....|....|....|....|....|....|....|....|....|....|....|....|....|

**tRNA genes ----------------------------------------------------------------------------------------------------**

**Col-0**  **----------------------------------------------------------------------------------------------------**

**An-1**  **ATTTTTCATCTTGAATAAAAACATTTTGACAAGTTTTTTAATATAATTTATTAGCAATTGAAAATGTTTTTTTTTT-CATTTCATGGTTTAATTTGTTTG**

**C24**  **ATTTTTCATCTTGAATAAAAACATTTTGACAAGTTTTTTAATATAATTCATTAGCAACTGAAAATGTTTTTTTTTT-CATTTCATGGTTTAATTTGTTTG**

**KBS-Mac-74** **ATTTTTCATCTTGAATAAAAACATTTTGACAAGTTTTTTAATATAATTTATTAGCAATTGAAAATGTTTTTTTTTT-CATTTCATGGTTTAATTTGTTTG**

**Tos82-387**  **ATTTTTCATCTTGAATAAAAACATTTTGACAAGTTTTTTAATATAATTCATTAGCAACTGAAAATGTTTTTTTTTT-CATTTCATGGTTTAATTTGTTTG**

**Cvi**  **ATTTTTCATCTTGAATAAAAACATTTTGACAAGTTTTTTAATATAATTCATTAGCAACTGAAAATGTTTTTTTTTT-CGTTTTATGGTTTAATTTGTTTG**

**Kn-0**  **ATTTTTCATCTTGAATAAAAACATTTTGACAAGTTTTTTAATATAATTCATTAGCAACTGAAAATGTTTTTTTTTTTCATTTCATGGTTTAATTTGTTTG**

**Cdm-0**  **ATTTTTCATCTTGAATAAAAACATTTTGACAAGTTTTTTAATATAATTCATTAGCAACTGAAAATGTTTTTTTTTT-CATTTCATGGTTTAATTTGTTTG**

**Ler**  **ATTTTTCATCTTGAATAAAAACATTTTGACAAGTTTTTTAATATAATTCATTAGCAACTGAAAATGTTTTTTTTTT-CATTTCATGGTTTAATTTGTTTG**

**Fja-2-4**  **ATTTTTCATCTTGAATAAAAACATTTTGACAAGTTTTTTAATATAATTCATTAGCAACTGAAAATGTTTTTTTTTT-CATTTCATGGTTTAATTTGTTTG**

**Eri-1**  **ATTTTTCATCTTGAATAAAAACATTTTGACAAGTTTTTTAATATAATTCATTAGCAACTGAAAATGTTTTTTTTTT-CATTTCATGGTTTAATTTGTTTG**

**Kor3**  **ATTTTTCATCTTGAATAAAAACATTTTGACAAGTTTTTTAATATAATTCATTAGCAACTGAAAATGTTTTTTTTTT-CATTTCATGGTTTAATTTGTTTG**

**Kyo**  **ATTTTTCATCTTGAATAAAAACATTTTGACAAGTTTTTTAATATAATTCATTAGCAACTGAAAATGTTTTTTTTTT-CATTTCATGGTTTAATTTGTTTG**

**Tur4**  **----------------------------------------------------------------------------------------------------**

**Ty-1**  **----------------------------------------------------------------------------------------------------**

**Fly-2-2**  **----------------------------------------------------------------------------------------------------**

2410 2420 2430 2440 2450 2460 2470 2480 2490 2500

....|....|....|....|....|....|....|....|....|....|....|....|....|....|....|....|....|....|....|....|

**tRNA genes ----------------------------------------------------------------------------------------------------**

**Col-0**  **----------------------------------------------------------------------------------------------------**

**An-1**  **TGAAAATTGTTTTTGATAATTTATATGATTACTAAATAAGTAAATAACTGACTTGGATATATTAGATTTCTTAGCAAAAAATAAAATAATTAATAGATAA**

**C24**  **TGAAAATTGTTTTTGATAATTTATATGATTACTAAATAAGTAAATAACTGACTTGGATATATTAGATTTCTTAGCAAAAAATAAAATAATTAATAGATAA**

**KBS-Mac-74** **TGAAAATTGTTTTTGATAATTTATATGATTACTAAATAAGTAAATAACTGACTTGGATATATTAGATTTCTTAGCAAAAAATAAAATAATTAATAGATAA**

**Tos82-387**  **TGAAAATTGTTTTTGATAATTTATATGATTACTAAATAAGTAAATAACTGACTTGGATATATTAGATTTCTTAGCAAAAAATAAAATAATTAATAGATAA**

**Cvi**  **TGAAAATTGTTTTTGATAATTTATATGATTACTAAATAAATAAATAACTGACTTGGATATATTAGATTTCTTAGCAAAAAATAAAATAATTAATAGATAA**

**Kn-0**  **TGAAAATTGTTTTTGATAATTTATATGATTACTAAATAAGTAAATAACTGACTTGGATATATTAGATTTCTTAGCAAAAAATAAAATAATTAATAGATAA**

**Cdm-0**  **TGAAAATTGTTTTTGATAATTTATATGATTACTAAATAAGTAAATAACTGACTTGGATATATTAGATTTCTTAGCAAAAAATAAAATAATTAATAGATAA**

**Ler**  **TGAAAATTGTTTTTGATAATTTATATGATTACTAAATAAGTAAATAACTGACTTGGATATATTAGATTTCTTAGCAAAAAATAAAATAATTAATAGATAA**

**Fja-2-4**  **TGAAAATTGTTTTTGATAATTTATATGATTACTAAATAAGTAAATAACTGACTTGGATATATTAGATTTCTTAGCAAAAAATAAAATAATTAATAGATAA**

**Eri-1**  **TGAAAATTGTTTTTGATAATTTATATGATTACTAAATAAGTAAATAACTGACTTGGATATATTAGATTTTTTAGCAAAAAATAAAATAATTAATAGATAA**

**Kor3**  **TGAAAATTGTTTTTGATAATTTATATGATTACTAAATAAGTAAATAACTGACTTGGATATATTAGATTTTTTAGCAAAAAATAAAATAATTAATAGATAA**

**Kyo**  **TGAAAATTGTTTTTGATAATTTATATGATTACTAAATAAGTAAATAACTGACTTGGATATATTAGATTTTTTAGCAAAAAATAAAATAATTAATAGATAA**

**Tur4**  **----------------------------------------------------------------------------------------------------**

**Ty-1**  **----------------------------------------------------------------------------------------------------**

**Fly-2-2**  **----------------------------------------------------------------------------------------------------**

2510 2520 2530 2540 2550 2560 2570 2580 2590 2600

....|....|....|....|....|....|....|....|....|....|....|....|....|....|....|....|....|....|....|....|

**tRNA genes ----------------------------------------------------------------------------------------------------**

**Col-0**  **---------------------------------->>>>>>>>>>>>>>>>>>>>>>>>>>>>>>>>>>>>>>>>>>>>>>>>>>>>>>>>>>>>>>>>>>**

**An-1**  **ATAATTTATGATTT-GAACTTATTAAAGCAATAAGGGTTCATAGCTCAGTGGTAGAGCAATTGACTGCAGATCAATAGGTCACCGGTTCGAATCCGGTTG**

**C24**  **ATAATTTATGATTTTGAATTTATTAAAGCAATAAGGGTTCATAGCTCAGTGGTAGAGCAATTGACTGCAGATCAATAGGTCACCGGTTCGAACCCGGTTG**

**KBS-Mac-74** **ATAATTTATGATTT-GAACTTATTAAAGCAATAAGGGTTCATAGCTCAGTGGTAGAGCAATTGACTGCAGATCAATAGGTCACCGGTTCGAATCCGGTTG**

**Tos82-387**  **ATAATTTATGATTTTGAACTTATTAAAGCAATAAGGGTTCATAGCTCAGTGGTAGAGCAATTGACTGCAGATCAATAGATCACCGGTTCGAACCCGGTTG**

**Cvi**  **ATAATTTATGATTTTGAACTTATTAAAGCAATAAGGGTTCATAGCTCAGTGGTAGAGCAATTGACTGCAGATCAATAGGTCACCGGTTCGAACCCGGTTG**

**Kn-0**  **ATAATTTATGATTTTGAACTTATTAAAGCAATAAGGGTTCATAGCTCAGTGGTAGAGCAATTGACTGCAGATCAATAGGTCACCGGTTTGAACCCGGTTG**

**Cdm-0**  **ATAATTTATGATTTTGAACTTATTAAAGTAATAAGGGTTCATAACTCAGTGGTAGAGCAATTGACTGCAGATCAATAGGTCACCGGTTTGAACCCGGTTG**

**Ler**  **ATAATTTATGATTTTGAACTTATTAAAGTAATAAGGGTTCATAACTCAGTGGTAGAGCAATTGACTGCAGATCAATAGGTCACCGGTTTGAACCCGGTTA**

**Fja-2-4**  **ATAATTTATGATTTTGAACTTATTAAAGCAATAAGGGTTTATAGCTCAGTGGTAGAGCAATTGACTGCAGATCAATAGGTCACCGGTTTGAACCCGGTTG**

**Eri-1**  **ATAATTTATGATTTTAAACTTATTAAAGCAATAAGGATTCATAGCTCAGTGGTAGAGCAATTGACTGCAGATCAATAGGTCACCGGTTCGAATCCGGTTG**

**Kor3**  **ATAATTTATGATTTTGAACTTATTAAAGCAATAAGGGTTCATAGCTCAGTGGTAGAGCAATTGACTGCAGATCAATAGGTCACCGGTTTGAACCCGGTTG**

**Kyo**  **ATAATTTATGATTTTGAACTTATTAAAGCAATAAGGGTTCATAGCTCAGTGGTAGAGCAATTGACTGCAGATCAATAGGTCACCGGTTTGAACCCGGTTG**

**Tur4**  **----------------------------------------------------------------------------------------------------**

**Ty-1**  **----------------------------------------------------------------------------------------------------**

**Fly-2-2**  **----------------------------------------------------------------------------------------------------**

2610 2620 2630 2640 2650

....|....|....|....|....|....|....|....|....|....|....|.

**tRNA genes >>>>>>--------------------------------------------------**

**Col-0**  **------ATATGTTTT-AGTTTACCAAAAAAAATTAAATATCATCTTGAATAAAGAA**

**An-1**  **GGCCCTATATTTTTT-AGTTTACCAAAAAAATTTAAATATCATCTTGAATAAAGAA**

**C24**  **GGCCCTATATTTTTT-AGTTTATCAAAAAAATTTAAATATCATCTTGAATAAAGAA**

**KBS-Mac-74** **GGCCCTATATTTTTT-AGTTTACCAAAAAAATTTAAATATTATCTTGAATAAAGAA**

**Tos82-387**  **GATCCTATATTTTTT-AGTTTACCAAAAAAATTTAAATATCATCTTAAATAAAGAA**

**Cvi**  **GGCTCTATTTTTTTC-AGTTTACCAAAAAAAATTAAATATCATCTTGAATAAAGAA**

**Kn-0**  **GGCCCTATATTTTTT-AGTTTACCAAAAAAATTTAAATATCATCTTGAATAAAGAA**

**Cdm-0**  **GGCCCTATATTTTTT-AGTTTACCAAAAAAATTTAAATATCATCTTGAATAAAGAA**

**Ler**  **GGCCCTATATTTTTT-AGTTTACCAAAAAAATTTAAATATCATCTTGAATAAAGAA**

**Fja-2-4**  **GGCCCTATATTTTTT-AGTTTACCAAAAAAATTTAAATATCATCTTGAATAAAGAA**

**Eri-1**  **GGCCCTATATTTTTT-AGTTTACCAAAAAAATTTAAATATTATCTTGAATAAAGAA**

**Kor3**  **GGCCCTATATTTTTT-AGTTTACCAAAAAAATTTAAATATCATCTTGAATAAAGAA**

**Kyo**  **GGCCCTATATTTTTT-AGTTTACCAAAAAAATTTAAATATCATCTTGAATAAAGAA**

**Tur4**  **------ATATTTTTTCAGTTTGCCAAAAAAAATTAAATATCATCTTGAATAAAGAA**

**Ty-1**  **------ATATGTTTT-AGTTTACCAAAAAAAATTAAATATCATCTTGAATAAAGAA**

**Fly-2-2**  **------ATATTTTTTCAGTTTGCCAAAAAAAATTAAATATCATCTTGAATAAAGAA**

**Supplementary Figure S4**. Multiple sequence alignment of the tRNA-Cys gene mini-clusters on chromosome 2. Location of the tRNA genes are shown in the tRNA genes line as green boxes.

10 20 30 40 50 60 70 80 90 100

....|....|....|....|....|....|....|....|....|....|....|....|....|....|....|....|....|....|....|....|

**trna genes**  **---------------------------------------<<<<<<<<<<<<<<<<<<<<<<<<<<<<<<<<<<<<<<<<<<<<<<<<<<<<<<<<<<<<<**

**Col-0**  **GAAACCCAGTTTTTACTGGGCTTAAAAAAAAAAAATTAAGAGCCCAACCGGATTCGAACCGGTGACCTATTGATCTGCAGTCAATTGCTCTACCACTGAG**

**Ty-1**  **GAAACCCAGTTTTTACTGGGCTTAAAAAAAAAAAATTAAGAGCCCAACCGGATTCGAACCGGTGACCTATTGATCTGCAGTCAATTGCTCTACCACTGAG**

**An-1**  **GAAACCCAGTTTTTACTGGGCTTAAAAAAAAAAA-TTAAGAGCCCAACCGGATTCGAACCGGTGACCTATTGATCTGCAGTCAATTGCTCTACCACTGAG**

**C24**  **GAAACCCAGTTTTTTCTGGGCTTAAAAAAAAAAAATTAAGAGCCCAACCGGATTCGAACCGGTGACCTATTGATCTGCAGTCAATTGCTCTACCACTGAG**

**Cdm-0**  **GAAACCCAGTTTTTACTGGGCTTAAAAAAAAAAAATTAAGAGCCCAACCGGATTCGAACCGGTGACCTATTGATCTGCAGTCAATTGCTCTACCACTGAG**

**KBS-Mac-74**  **GAAACCCAGTTTTTTCTGGGCTTAAAACAAAAAAATTAAGAGCCCAACCGGATTCGAACCGGTGACCTATTGATCTGCAGTCAATTGCTCTACCACTGAG**

**Kn-0**  **GAAACCCAGTTTTTACTGGGCTTAAAAAAAAAAAATTAAGAGCCCAACCGGATTCGAACCGGTGACCTATTGATCTGCAGTCAATTGCTCTACCACTGAG**

**Cvi**  **GAAACCCAGTTTTTTCTGGGTTTAAAAAAAAACAATTAAGAGCCCAACC-----------GGTGACCTATTGATCTGCAGTCAATTGCTCTACCACTGAG**

**Eri**  **GAAACCCAGTTTTTTCTGGGCTTAAAAAAAAAAA-TAAAGAGCCCAACCGGATTCGAACCGGTGACCTATTGATCTGCAGTCAATTGCTCTACCACTGAG**

**Kyo**  **GAAACCCAGTTTTTTCTGGGCTTTAAAAAAAAAAATTAAGAGCCCAACCGGATTCGAACCGGTGACCTATTGATCTGCAGTCAATTGCTCTACCACTGAG**

**Ler**  **GAAACCCAGTTTTTTCTGGGCTTTAAAAAAAAAAATTAAGAGCCCAACCGGATTCGAACCGGTGACCTATTGATCTGCAGTCAATTGCTCTACCACTGAG**

**Fja-2-4**  **GAAACCCAGTTTTTACTGGGCTTAAAAAAAAAAAATTAAGAGCCCAACCGGATTCGAACCGGTGACCTATTGATCTGCAGTCAATTGCTCTACCACTGAG**

**Kor3**  **GAAACCCAGTTTTTACTGGGCTTAAAAAAAAAAAATTAAGAGCCCAACCGGATTCGAACCGGTGACCTATTGATCTGCAGTCAATTGCTCTACCACTGAG**

**Tos82-387**  **GAAACCCAGTTTTTTCTGGGCTTAAAACAAAAAAATTAAGAGCCCAACCGGATTCGAACCGGTGACCTATTGATCTGCAGTCAATTGCTCTACCACTGAG**

**Tur-4**  **GAAACCCAGTTTTTTCTGGGCTTAAAAAAAAAAAATTAAGAGCCCAACCGGATTCGAACCGGTGACCTATTGATCTGCAGTCAATTGCTCTACCACTGAG**

**Fly-2-2**  **GAAACCCAGTTTTTACTGGGCTTAAAAAAAAAAAATTAAGAGCCCAACCGGATTCGAACCGGTGACCTATTGATCTGCAGTCAATTGCTCTACCACTGAG**

110 120 130 140 150 160 170 180 190 200

....|....|....|....|....|....|....|....|....|....|....|....|....|....|....|....|....|....|....|....|

**tRNA genes**  **<<<<<<<<<<------------------------------------------------------------------------------------------**

**Col-0**  **CTATAGGCTCTTATTGACTTTAGTGTATACTACTTATATATAACTTAACAATTATGTTTCTCCAAAATTCTGTTTTAAGTAAAATAATGTTCTATCATGC**

**Ty-1**  **CTATAGGCTCTTGTTGACTTTAGTGTATACTACTTATATATAACTTAACAATTATGTTTCTCCAAAATTCTGTTTTAAGTAAAATAATGTTTTATCATGC**

**An-1**  **CTATAGGCTCTTGTTGACTTTAGTGTATACTACTTATATATAACTTAACAATTATGTTTCTCCAAAATTCTGTTTTAAGTAAAATAATGTTTTATCATGC**

**C24**  **CTATAGGCTCTTGTTGACTTTAGTGTATACTACTTATATATAACTTAACAATTATGTTTCTCCAAAATTCTGTTTTAAGTAAAATAATGTTTTATCATGC**

**Cdm-0**  **CTATAGGCTCTTGTTGACTTTAGTGTATACTACTTATATATAACTTAACAATTATGTTTCTCCAAAATTCTGTTTTAAGTAAAATAATGTTTTATCATGC**

**KBS-Mac-74**  **CTATAGGCTCTTGTTGACTTTAGTGTATACTACTTATATATAACTTAACAATTATGTTTCTCCAAAATTCTGTTTTAAGTAAAATAATGTTTTATCATGC**

**Kn-0**  **CTATAGGCTCTTGTTGACTTTAGTGTATACTACTTATATATAACTTAACAATTATGTTTCTCCAAAATTCTGTTTTAAGTAAAATAATGTTTTATCATGC**

**Cvi**  **CTATAGGCTCTTGTTGACTTTAGTGTATACTACTTATATATAACTTAACAATTATGTTTCTTCAAAATTCTGTTTTAAGTAAAATAATGTTTTATCATGC**

**Eri**  **CTATAGGCTCTTGTTGACTTTAGTGTATACTACTTATATATAACTTAACAATTATGTTTCTCCAAAATTCTGTTTTAAGTAAAATAATGTTTTATCATGC**

**Kyo**  **CTATAGGCTCTTGTTGACTTTAGTGTATACTACTTATATATAACTTAACAATTATGTTTCTCCAAAATTCTGTTTTAAGTAAAATAATGTTTTATCATGC**

**Ler**  **CTATAGGCTCTTGTTGACTTTAGTGTATACTACTTATATATAACTTAACAATTATGTTTCTCCAAAATTCTGTTTTAAGTAAAATAATGTTTTATCATGC**

**Fja-2-4**  **CTATAGGCTCTTGTTGACTTTAGTGTATACTACTTATATATAACTTAACAATTATGTTTCTCCAAAATTCTGTTTTAAGTAAAATAATGTTTTATCATGC**

**Kor3**  **CTATAGGCTCTTGTTGACTTTAGTGTATACTACTTATATATAACTTAACAATTATGTTTCTCCAAAATTCTGTTTTAAGTAAAATAATGTTTTATCATGC**

**Tos82-387**  **CTATAGGCTCTTGTTGACTTTAGTGTATACTACTTATATATAACTTAACAATTATGTTTCTCCAAAATTCTGTTTTAAGTAAAATAATGTTTTATCATGC**

**Tur-4**  **CTATAGGCTCTTGTTAACTTTAGTGTATACTACTTATATATAACTTAACAATTATGTTTCTCCAAAATTCTGTTTTAAGTAAAATAATGTTTTATCATGC**

**Fly-2-2**  **CTATAGGCTCTTGTTGACTTTAGTGTATACTACTTATATATAACTTAACAATTATGTTTCTCCAAAATTCTGTTTTAAGTAAAATAATGTTTTATCATGC**

210 220 230 240 250 260 270 280 290 300

....|....|....|....|....|....|....|....|....|....|....|....|....|....|....|....|....|....|....|....|

**tRNA genes**  **------------------------------------------->>>>>>>>>>>>>>>>>>>>>>>>>>>>>>>>>>>>>>>>>>>>>>>>>>>>>>>>>**

**Col-0**  **AAGTTTAGTATTTCTATTAGTTAGCATACACTAAAGTCAAGAAGAGCCTATAGCTCAGTGGTAGAGCAATTGACTGCAGATCAATAGGTCACCGGTTCGA**

**Ty-1**  **AAGTTTAGTATTTGTATTAGTTAGCATACACTAAAGTCAAGAAGAGCCTATAGCTCAGTGGTAGAGCAATTGACTGCAGATCAATAGGTCACCGGTTCGA**

**An-1**  **AAGTTTAGTATTTCTATTAGTTAGCATACACTAAAGTCAAGAAGAGCCTATAGCTCAGTGGTAGAGCAATTGACTGCAGATCAATAGGTCACCGGTTCGA**

**C24**  **AAGTTTAGTATTTCTATTAGTTAGCATACACTAAAGTCAAGAAGAGCCTATAGCTCAGTGGTAGAGCAATTGACTGCAGATCAATAGGTCACCGGTTCGA**

**Cdm-0**  **AAGTTTAGTATTTGTATTAGTTAGCATACACTAAAGTCAAGAAGAGCCTATAGCTCAGTGGTAGAGCAATTGACTGCAGATCAATAGGTCACCGGTTCGA**

**KBS-Mac-74**  **AAGTTTAGTATTTCTATTAGTTAGCATACACTAAAGTCAAGAAGAGCCTATAGCTCAGTGGTAGAGCAATTGACTGCAGATCAATAGGTCACCGGTTCGA**

**Kn-0**  **AAGTTTAGTATTTGTATTAGTTAGCATACACTAAAGTCAAGAAGAGCCTATAGCTCAGTGGTAGAGCAATTGACTGCAGATCAATAGGTCACCGGTTCGA**

**Cvi**  **AAGTTTAGTATTTCTATTAGTTAGCATACACTAAAGTCAAGAAGAGCCTATAGCTCAGTGGTAGAGCAATTGACTGCAGATCAATAGGTCACCGGTTCGA**

**Eri**  **AAGTTTAGTATTTGTATTAGTTAGCATACACTAAAGTCAAGGAGAGCCTATAGCTCAGTGGTAGAGCAATTGACTGCAGATCAATAGGTCACCGGTTCGA**

**Kyo**  **AAATTTAGTATTTCTATTAGTTAGCATACACTAAAGTCAAGAAGAGCCTATAGCTCAGTGGTAGAGCAATTGACTGCAGATCAATAGGTCACCGGTTCGA**

**Ler**  **AAATTTAGTATTTCTATTAGTTAGCATACACTAAAGTCAAGAAGAGCCTATAGCTCAGTGGTAGAGCAATTGACTGCAGATCAATAGGTCACCGGTTCGA**

**Fja-2-4**  **AAGTTTAGTATTTCTATTAGTTAGCATACACTAAAGTCAAGAAGAGCCTATAGCTCAGTGGTAGAGCAATTGACTGCAGATCAATAGGTCACCGGTTCGA**

**Kor3**  **AAGTTTAGTATTTCTATTAGTTAGCATACACTAAAGTCAAGAAGAGCCTATAGCTCAGTGGTAGAGCAATTGACTGCAGATCAATAGGTCACCGGTTCGA**

**Tos82-387**  **AAGTTTAGTATTTCTATTAGTTAGCATACACTAAAGTCAAGAAGAGCCTATAGCTCAGTGGTAGAGCAATTGACTGCAGATCAATAGGTCACCGGTTCGA**

**Tur-4**  **AAGTTTAGTATTTCTATTAGTTAGCATACACTAAAGTCAAGAAGAGCCTATAGCTCAGTGGTAGAGCAATTGACTGCAGATCAATAGGTCACCGGTTCGA**

**Fly-2-2**  **AAGTTTAGTATTTCTATTAGTTAGCATACACTAAAGTCAAGAAGAGCCTATAGCTCAGTGGTAGAGCAATTGACTGCAGATCAATAGGTCACCGGTTCGA**

310 320 330 340 350 360 370 380 390

....|....|....|....|....|....|....|....|....|....|....|....|....|....|....|....|....|....|....|....

**tRNA genes**  **>>>>>>>>>>>>>>-------------------------------------------------------------------------------------**

**Col-0**  **ATCCGGTTGGGCTCTTTATTTTTTGTCTCTTAACAGAGATTTCAACAGATTTTCCAATATTTCTCACAATTTCAATTATACTTAGAAAGATTTTTTGAT**

**Ty-1**  **ATCCGGTTGGGCTCTTTATTTTTTGTCTCTTAACAGAGATTTCAACAGATTTTCCGATATTTCTCACAATTTCAATTATACTTAGAAAGATTTTTTGAT**

**An-1**  **ATCCGGTTGGGCTCTTTATTTTTTGTCTCTTAACAGAGATTTCAACAGATTTTCCGATATTTCTCACAATTTCAATTATACTTAGAAAGATTTTTTGAT**

**C24**  **ATCCGGTTGGGCTCTTTAATTTT-GTCTCTTAACAGAGATTTCAACTGATTTTCCGATATTTC----------AATTATACTTAGAAAGATTTTTTGAT**

**Cdm-0**  **ATCCGGTTGGGCTCTTTATTTTTTGTCTCTTAACAGAGATTTCAACAGATTTTCCGATATTTCTCACAATTTCAATTATACTTAGAAAGATTTTTTGAT**

**KBS-Mac-74**  **ATCCGGTTGGGCTCTTTAATTTT-GTCTCTTAACAGAGATTTCAACTGATTTTCCGATATTTC----------AATTATACTTAGAAAGATTTTTTGAT**

**Kn-0**  **ATCCGGTTGGGCTCTTTATTTTTTGTCTCTTAACAGAGATTTCAACAGATTTTCCGATATTTCTCACAATTTCAATTATACTTAGAAAGATTTTTTGAT**

**Cvi**  **ATCCGGTTGGGCTCTTTATTTTTTGTCTCTTAACAGAGATTACGACAGATTTTCCGATATTTCTCACAATTTCAATTATACTTAGAAAGATTTTTTGAT**

**Eri**  **ATCCGGTTGGGCTCTTTATTTTTTGTCTGTTAACAGAGATTTCAACAGATTTTCCGATATTTCTCACAATTTCAATTATACTTAGAAAGATTTTTTGAT**

**Kyo**  **ATCCGGTTGGGCTCTTTATTTTTTGTCTCTTAACAGAGATTTCAACAGATTTTCCGATATTTCTCACAATTTCAATTATACTTAGAAAGATTTTTTGAT**

**Ler**  **ATCCGGTTGGGCTCTTTATTTTTTGTCTCTTAACAGAGATTTCAACAGATTTTCCGATATTTCTCACAATTTCAATTATACTTAGAAAGATTTTTTGAT**

**Fja-2-4**  **ATCCGGTTGGGCTCTTTATTTTTTGTCTCTTAACAGAGATTTCAACAGATTTTCCGATATTTCTCACAATTTCAATTATACTTAGAAAGATTTTTTGAT**

**Kor3**  **ATCCGGTTGGGCTCTTTATTTTTTGTCTCTTAACAGAGATTTCAACAGATTTTCCGATATTTCTCACAATTTCAATTATACTTAGAAAGATTTTCTGAT**

**Tos82-387**  **ATCCGGTTGGGCTCTTTAATTTT-GTCTCTTAACAGAGATTTCAACTGATTTTCCGATATTT----------CAATTATACTTAGAAAGATTTTTTGAT**

**Tur-4**  **ATCCGGTTGGGCTCTTTAATTTT-GTCTCTTAACAGAGATTTCAACTGATTTTCCGATATTT----------CAATTATACTTAGAAAGATTTTTTGAT**

**Fly-2-2**  **ATCCGGTTGGGCTCTTTATTTTTTGTCTCTTAACAGAGATTTCAACAGATTTTCCGATATTTCTCACAATTTCAATTATACTTAGAAAGATTTTTTGAT**

**Supplementary Figure S5**. Multiple sequence alignment of the *Arabidopsis thaliana* tRNA-Cys gene mini-clusters on chromosome 1. Location of the tRNA genes are shown in the tRNA genes line as green boxes, Note that the orientation of of the genes is reversed relative to the orientation shown in Fig. 4B.

10 20 30 40 50 60 70 80 90 100

....|....|....|....|....|....|....|....|....|....|....|....|....|....|....|....|....|....|....|....|

**tRNA genes** **-------------------------------------------------->>>>>>>>>>>>>>>>>>>>>>>>>>>>>>>>>>>>>>>>>>>>>>>>>>**

**Col-0 TCTCAACCTAATGAAATATTAATTGACAAGTGTTATGCAGAACATTAAAAGGGTCCTTAGCTCAGTGGTAGAGCAATTGACTGCAGATCAATAGGTCACC**

**Ty-1 TCTCAACCTAATGAAATATTAATTGACAAGTGTTATGCAGAACATTAAAAGGGTCCTTAGCTCAGTGGTAGAGCAATTGACTGCAGATCAATAGGTCACC**

**C24 TCTCAACCTAATGAAATATTAATTGACAAGTGTTATGCAGAACATTAAAAGGGTCCTTAGCTCAGTGGTAGAGCAATTGACTGCAGATCAATAGGTCACC**

**An-1 TCTCAACCTAATGAAATATTAATTGACAAGTGTTATGCAGAACATTAAAAGGGTCCTTAGCTCAGTGGTAGAGCAATTGACTGCAGATCAATAGGTCACC**

**Cdm-0 TCTCAACCTAATGAAATATTAATTGACAAGTGTTATGCAGAACATTAAAAGGGTCCTTAGCTCAGTGGTAGAGCAATTGACTGCAGATCAATAGGTCAAC**

**KBS-Mac-74 TCTCAACCTAATGAAATATTAATTGACAAGTGTTATGCAGAACATTAAAAGGGTCCTTAGCTCAGTGGTAGAGCAATTGACTGCAGATCAATAGGTCACC**

**Kn-0 TCTCAACCTAATGAAATATTAATTGACAAGTGTTATGCAGAACATTAAAAGGGTCCTTAGCTCAGTGGTAGAGCAATTGACTGCAGATCAATAGGTCACC**

**Cvi TCTCAACCTAATGAAATATTAATTGACAAGTGTTATGCAGAACATTAAAAGGGTCCTTAGCTCAGTGGTAGAGCAATTGACTGCAGATCAATAGGTCCCC**

**Eri TCTCAACCTAATGAAATATTAATTGACAAGTGTTATGCAGAACATTAAAAGGGTCCTTAGCTCAGTGGTAGAGCAATTGACTGCAGATCAATAGGTCACC**

**Ler-0 TCTCAACCTAATGAAATATTAATTGACAAGTGTTATGCAGAACATTAAAAGGGTCCTTAGCTCAGTGGTAGAGCAATTGACTGCAGATCAATAGGTCACC**

**Kyo TCTCAACCTAATGAAATATTAATTGACAAGTGTTATGCAGAACATTAAAAGGGTCCTTAGCTCAGTGGTAGAGCAATTGACTGCAGATCAATAGGTCACC**

**Fja-2-4 TCTCAACCTAATGAAATATTAATTGACAAGTGTTATGCAGAACATTAAAAGGGTCCTTAGCTCAGTGGTAGAGCAATTGACTGCAGATCAATAGGTCACC**

**Fly-2-2 TCTCAACCTAATGAAATATTAATTGACAAGTGTTATGCAGAACATTAAAAGGGTCCTTAGCTCAGTGGTAGAGCAATTGACTGCAGATCAATAGGTCACC**

**Tos-82-387 TCTCAACCTAATGAAATATTAATTGACAAGTGTTATGCAGAACATTAAAAGGGTCCTTAGCTCAGTGGTAGAGCAATTGACTGCAGATCAATAGGTCACC**

**Kor3 TCTCAACCTAATGAAATATTAATTGACAAGTGTTATGCAGAACATTAAAAGGGTCCTTAGCTCAGTGGTAGAGCAATTGACTGCAGATCAATAGGTCACC**

**Tur-4 TCTCAACCTAATGAAATATTAATTGACAAGTGTTATGCAGAACATTAAAAGGGTCCTTAGCTCAGTGGTAGAGCAATTGACTGCAGATCAATAGGTCACC**

110 120 130 140 150 160 170 180 190 200

....|....|....|....|....|....|....|....|....|....|....|....|....|....|....|....|....|....|....|....|

**tRNA**  **>>>>>>>>>>>>>>>>>>>>>-------------------------------------------------------------------------------**

**Col-0**  **GGTTCGAATCCGGTAGGGCCCTTGATTTTTTATTTTTTTCCTCCTTCC----------------------------------------------------**

**Ty-1 GATTCGAATCCGGTAGGGCCCTTGATTTTTTATTTTTTTCCTCCTTCCAAAGCCCTAATATCCCTATTTTACAGAAACCATTCCATGATAAATCAATGAC**

**C24 GGTTCGAATCCGGTAGGGCCCTTGATTTTTTATTTTTTTCCTCCTTCCAAAGCCCTAATATCCCTATTTTACAGAAACCATTCCATGATAAATCAATGAC**

**An-1 GGTTCGAATCCGGTAGGGCCCTT-----------------------------------------------------------------------------**

**Cdm-0 GGTTCGAATCCGGTAGGGCCCTTGATTTTTTATTTTTTTCCTCCTTCCAAAGCCCTAATATCCCTATTTTACAGAAACCATTCCATGATAAATCAATGAC**

**KBS-Mac-74 GGTTCGAATCCGGTAGAGCCCTTGATTTTTTATTTTTTTCCTCCTTCCAAAGCCCTAATATCCCTATTTTACAGAAACCATTCCATGATAAATCAATGAC**

**Kn-0 GGTTCGAATCCGGTAGGGCCCTTGATTTTTTAATTTTTTCCTCCTTCCAAAGCCCTAATATCCCTATTTTACAGAAACCATTCCATGATAAATCAATGAC**

**Cvi GGTTCGAATCCGGTAGGGCCCTTGATTTTTTATTTTTTTCCTCCTTCCAAAGCCCTAATATCCCTATTTTACAGAAACCATTCCATGATAAATCAATGAC**

**Eri**  **GGTTCGAATCCGGTAGGGCCCTT-----------------------------------------------------------------------------**

**Ler-0 GGTTCGAATCCGGTAGGGCCCTTGATTTTTT---------------------------------------------------------------------**

**Kyo GGTTCGAATCCGGTAGGGCCCTTGATTTTTTATTTTTTTCCTCCTTCC----------------------------------------------------**

**Fja-2-4 GGTTCGAATCCGGTAGGGCCCTTGATTTTTTATTTTTTTCCTCCTTCC----------------------------------------------------**

**Fly-2-2 GGTTCGAATCCGGTAGGGCCCTTGATTTTTTATTTTTTTCCTCCTTCC----------------------------------------------------**

**Tos-82-387 GGTTCGAATCCGGTAGGGCCCTTGATTTTTTATTTTTTTCCTCCTTCC----------------------------------------------------**

**Kor3 GGTTCGAATCCGGTAGAGCCCTTGATTTTTTATTTTTTTCCTCCTTCCAAAGCCCTAATATCCCTATTTTACAGAAACCATTCCATGATAAATCAATGAC**

**Tur-4 GGTTCGAATCCGGTAGAGCCCTTGATTTTTTATTTTTTTCCTCCTTCCAAAGCCCTAATATCCCTATTTTACAGAAACCATTCCATGATAAATCAATGAC**

210 220 230 240 250 260 270 280 290 300

....|....|....|....|....|....|....|....|....|....|....|....|....|....|....|....|....|....|....|....|

**tRNA**  **------------------------------------------>>>>>>>>>>>>>>>>>>>>>>>>>>>>>>>>>>>>>>>>>>>>>>>>>>>>>>>>>>**

**Col-0 ----------------------------------------------------------------------------------------------------**

**Ty-1 TCATATTCATCTCTCAGTGTAACTCATTGTCTAGCAACATGTGGGTCCTTAGCTCAGTGGTAGAGCAATTGACTGCAGATCAATAGGTCACCGGTTCGAA**

**C24 TCATATTCATCTCTCAGTGTAACTCATTGTCTAGCAACATGAGGGTCCTTAGCTCAGTGGTAGAGCAATTGACTGCAGATCAATAGGTCACCGGTTCGAA**

**An-1 ----------------------------------------------------------------------------------------------------**

**Cdm-0 TCATATTCATCTCTCAGTGTAACTCATTGTCTAGCAACATGAGGGTCCTTAGCTCAGTGGTAGAGCAATTGACTGCAGATCAATAGGTCACCGGTTCGAA**

**KBS-Mac TCATATTCATCTCTCAGTGTAACTCATTGTCTAGCAACATGAGGGTCCTTAGCTCAGTGGTAGAGCAATTGACTGCAGATCAATAGGTCACTGGTTCGAA**

**Kn-0 TCATATTCATCTCTCAGTGTAACTCATTGTCTAGCAACATGAGGGTCCTTAGCTCAGTGGTAGAGCAATTGACTGCAGATCAATAGGTCACCGGTTCGAA**

**Cvi TCATATTCATCTCTCAGTGTAACTCATTGTCTAGCAACATGAGGGTCCTTAGCTCAGTGGTAGAGCAATTGACTGCAGATCAATAGGTCACCGGTTCGAA**

**Eri ----------------------------------------------------------------------------------------------------**

**Ler-0 ----------------------------------------------------------------------------------------------------**

**Kyo ----------------------------------------------------------------------------------------------------**

**Fja-2-4 ----------------------------------------------------------------------------------------------------**

**Fly-2-2 ----------------------------------------------------------------------------------------------------**

**Tos-82-387 ----------------------------------------------------------------------------------------------------**

**Kor3 TCATATTCATCTCTCAGTGTAACTCATTGTCTAGCAACATGAGGGTCCTTAGCTCAGTGGTAGAGCAATTGACTGCAGATCAATAGGTCACCGGTTCGAA**

**Tur-4 TCATATTCATCTCTCAGTGTAACTCATTGTCTAGCAACATGAGGGTCCTTAGCTCAGTGGTAGAGCAATTGACTGCAGATCAATAGGTCACCGGTTCGAA**

310 320 330 340 350 360 370 380 390 400

....|....|....|....|....|....|....|....|....|....|....|....|....|....|....|....|....|....|....|....|

**tRNA >>>>>>>>>>>>>---------------------------------------------------------------------------------------**

**Col-0 -------------------------------------------------------ATCCATTTGTCACAAAATCCCTGCAGAAGAATGACTACCTATCTA**

**Ty-1 TCCGGTAGGGCCCTTAAAAGTC--TTTTTTTTAACCCTCCCTCCCAATAAGTAACATCCATTTGTCACAAAATCCCTGCAGAAGAATGACTACCTATCTA**

**C24 TCCGGTAGGGCCCTT-AAAGTC-TTTTTTTTTAACCCTCCCTCCCAATAAGTAACATCCATTTGTCACAAAATCCCTGCAGAAGAATGACTACCTATCTA**

**An-1 ---------------AAAAGTC-TTTTTTTTTAACCCTCCCTCCCAATAAGTAACATCCATTTGTCACAAAATCCCTGCAGAAGAATGACTACCTATCTA**

**Cdm-0 TCCGGTAGGGCCCTT-AAAGTC-TTTTTTTTTAACCCTCCCTCCCAATAAGTAACATCCATTTGTCACAAAATCCCTGCAGAAGAATGACTACCTATCTA**

**KBS-Mac TCCGGTAGGGCCCTTAAAAGTC-TTTTTTTTTAACCCTCCCTCCCAATAAGTAACATCCATTTGTCACAAAATCCCTGCAGAAGAATGACTACCTATCTA**

**Kn-0 TCCGGTAGGGCCCTTAAAAGTC-TTTTTTTTTAACCCTCCCTCCCAATAAGTAACATCCATTTGTCACAAAATCCCTGCAGAAGAATGACTACCTATCTA**

**Cvi TCCGGTAGGGCCCTTAAAAGTCTTTTTTTTTTAACCCTCCCTCCCAATAAGTAACATCCATTTGTCACAAAATCCCTGCAGAAGAATGACTACCTATCTA**

**Eri ---------------AAAAGTC-TTTTTTTTTAACCCTCCCTCCCAATAAGTAACATCCATTTGTCACAAAATCCCTGCAGAAGAATGACTACCTATCTA**

**Ler-0 ------------------------TTTTTTTTAACCCTCCTTCCCAATAAGTAACATCCATTTGTCACAAAATCCCTGCAGAAGAATGACTACCTATCTA**

**Kyo -------------------------------------------------------ATCCATTTGTCACAAAATCCCTGCAGAAGAATGACTACCTATCTA**

**Fja-2-4 -------------------------------------------------------ATCCATTTGTCACAAAATCCCTGCAGAAGAATGACTACCTATCTA**

**Fly-2-2 -------------------------------------------------------ATCCATTTGTCACAAAATCCCTGCAGAAGAATGACTACCTATCTA**

**Tos-82-387 -------------------------------------------------------ATCCATTTGTCACAAAATCCCTGCAGAAGAATGACTACCTATCTA**

**Kor3 TCCGGTAGGGCCCTTAAAAGTC-TTTTTTTTTAACCCTCCCTCCCAATAAGTAACATCCATTTGTCACAAAATCCCTGCAGAAGAATGACTACCTATCTA**

**Tur-4 TCCGGTAGGGCCCTTAAAAGTC-TTTTTTTTTAACCCTCCCTCCCAATAAGTAACATCCATTTGTCACAAAATCCCTGCAGAAGAATGACTACCTATCTA**

**Supplementary Figure S6**. Multiple sequence alignment of the tRNA-Cys gene mini-clusters on chromosome 1 from various Arabidopsis species. Location of the tRNA genes and pseudogenes are shown in the tRNA genes line as green and red boxes, respectively. Note that the orientation of of the genes is reversed relative to the orientation shown in Fig. 5.

**10 20 30 40 50 60 70 80 90 100**

**....|....|....|....|....|....|....|....|....|....|....|....|....|....|....|....|....|....|....|....|**

**tRNA genes ----------------------------------------------------------------------------------------------------**

**Col-0 CACTGACACAATGTAGTTGCCCTGAAGTTCAATAGTAAACATATGAAACTCTCAACCTAATGAAATATTAATTGACAAGTGTTATGCAGAACATT-AAAA**

**Cvi CACTGACACAATGTAGTTGCCCTGAAGTTCAATAGTAAACATATGAAACTCTCAACCTAATGAAATATTAATTGACAAGTGTTATGCAGAACATT-AAAA**

**A. suecica CACTGACACAATGTAGTTGCCCTGAAGTTCAATAGTAAACATATGAAACTCTCAACCTAATGAAATATTAATTGACAAGTGTTATGCAGAACATT-AAAA**

**A. arenosa AACTGAGACAATCAAATTGCCATGAAGTTCACTGGTAAGCATTTAAAATTCTCAACCTAATGAAATGTTATTTAACAAGTGCTATGAAGAACACCAAAAA**

**A. lyrata AACTGAGACAATCAAATTGCCATGAAGTTCACCgataaacatttaaaattctCAACCTAATGAAATGTTATTTAACAAGTGCTATGAAGAACACCAAAAA**

**A. halleri AACTGAGACAATCAAATTGCCATGAAGTTCACTGGTAAACATTTAAAATTCTCAACCTAATGAAATGTTATTTTACATGTGCTATGAAGAACACCAAAAA**

**110 120 130 140 150. 160 170 180 190 200**

**....|....|....|....|....|....|....|....|....|....|....|....|....|....|....|....|....|....|....|....|**

**tRNA genes >>>>>>>>>>>>>>>>>>>>>>>>>>>>>>>>>>>>>>>>>>>>>>>>>>>>>>>>>>>>>>>>>>>>>>>>----------------------------**

**Col-0. GGGTCCTTAGCTCAGTGGTAGAGCAATTGACTGCAGATCAATAGGTCACCGGTTCGAATCCGGTAGGGCCCTTGATTTTTTATTTTTTT---CCTCCTTC**

**Cvi. GGGTCCTTAGCTCAGTGGTAGAGCAATTGACTGCAGATCAATAGGTCCCCGGTTCGAATCCGGTAGGGCCCTTGATTTTTTATTTTTTT---CCTCCTTC**

**A. suecica GGGTCCTTAGCTCAGTGGTAGAGCAATTGACTGCAGATCAATAGGTCACCGGTTCGAATCCGGTAGGGCCCTTGATTTTTTATTTTTTT---CCTCCTTC**

**A. arenosa GGGTCCTTAGCTCAGTGGTAGAGCAATTGACTGCAGATCAATAGGTCACCGGTTCGAATCCGGTAGGGCCCTTACACTTCTCTTTTTTT---CCTCCTTC**

**A. lyrata GGGTCCTTAGCTCAGTGGTAGAGCAATTGACTGCAGATCAATAGGTCACCGGTTCGAATCCGGTAGGGCCCTTTAAATTTTTATTTTTAT--CCTCCTTC**

**A. halleri GGGTCCTTAGCTCAGTGGTAGAGCAATTGACTGCAGATCAATAGGTCACCGGTTCGAATCCGGTAGGGCCCTTAATTTTTTTTTTTTCTTTTCCTCCTTC**

**210 220 230 240 250 260 270 280 290 300**

**....|....|....|....|....|....|....|....|....|....|....|....|....|....|....|....|....|....|....|....|**

**tRNA genes ----------------------------------------------------------------------------------------------------**

**Col-0 C---------------------------------------------------------------------------------------------------**

**Cvi CAAAGCCCTAAT--ATCCCTATTTTACAGAAACCATTCCATGATAAATCAATGACTCAT-----ATTCATCTCTCAGTGTAACTCATTGTCTAGCAACAT**

**A. suecica CAAAGCCCTAAT--ATCCCTATTTTACAGAAACCATTCCATGATAAATCAATGACTCAT-----ATTCATCTCTCAGTGTAACTCATTGTCTAGCAACAT**

**A. arenosa CCAATCCCTAATATATCCCTATTGTACATATACTATTTCATGGTAAATCAATGACTC-------ATTCATCTCTATGTGTAACTCATTCCCTAACAACCA**

**A. lyrata CCAATCCC-----------TATTGTAGAAATAACATTTCATGGTAAATCAATGACTC-------ATTCATCTCTATGTGTAACTCATTCTCTAGCAACCA**

**A. helleri CCAATCCCTATT------GTAGAAATACAGTACTATTTCATCGTAAATCAATGACTCATTCATGATTCATCTCTATGTGTAACTCATTCTCTAACAACCA**

**310 320 330 340 350 360 370 380 390 400**

**....|....|....|....|....|....|....|....|....|....|....|....|....|....|....|....|....|....|....|....|**

**tRNA genes -->>>>>>>>>>>>>>>>>>>>>>>>>>>>>>>>>>>>>>>>>>>>>>>>>>>>>>>>>>>>>>>>>>>>>>>>--------------------------**

**Col-0 ----------------------------------------------------------------------------------------------------**

**Cvi GAGGGTCCTTAGCTCAGTGGTAGAGCAATTGACTGCAGATCAATAGGTCACCGGTTCGAATCCGGTAGGGCCCTTAAAAGTCTTTTTTTTTTAACCCTCC**

**A. suecica GAGGGTCCTTAGCTCAGTGGTAGAGCAATTGACTGCAGATCAATAGGTCACCGGTTCGAATCCGGTAGGGCCCTTA-AAGTCTTTTTTTTT-AACCCTCC**

**A. arenosa AAGGGTCCTTAGCTCAGTGGTAGAGCAATTGACTGCAGATCAATAGGCCACCGGTTCGAATCCGGTAGGGCCCTTA-TAACTTTTTTTTTT-ATCCC---**

**A. lyrata AAGGGTCCTTAGCTCAGTGGTAGAGCAATTGACTGCAGATCAATAGGTCACCGGTTCAAATCCGGTAGGGCCCTTA---AAACTCTTTTTT-ATCCCTCC**

**A. halleri AAGGGTCCTTAGCTCAGTGGTAGAGCAATTGA--------------------------------------------------------------------**

**410 420 430 440 450 460 470 480 490 500**

**....|....|....|....|....|....|....|....|....|....|....|....|....|....|....|....|....|....|....|....|**

**tRNA genes ----------------------------------------------------------------------------------------------------**

**Col-0 --------------------ATCCATTT-GTCACAAAATCCCTGCAGAAGAATGACTACCTATCTATCCTGTTTATGTATAACTCATTCCT---------**

**Cvi CTCCCAA-----TAAGTAACATCCATTT-GTCACAAAATCCCTGCAGAAGAATGACTACCTATCTATCCTGTTTATGTATAACTCATTCCT---------**

**A. suecica CTCCCAA-----TAAGTAACATCCATTT-GTCACAAAATCCCTGCAGAAGAATGACTACCTATCTATCCTGTTTATGTATAACTCATTCCT---------**

**A. arenosa ------aaACATTAATTG-TATCCGTTTTTTCACAAAATCCCTGCAGAACAATGACT----ATCTATCCTGTTTATGTATAACTAATTCCTAGAAAACCA**

**A. lyrata CTCC-----GAATCATTAATATCCATTTTTTCACAAAATCCCTGTAGAACAATGACT----ATCTATCCTGTTTATGTAAAACTCATTCCTACAAAACCA**

**A. halleri ----------------------------------------------------------------------------------------------------**

**510 520 530 540 550 560 570 580 590 600**

**....|....|....|....|....|....|....|....|....|....|....|....|....|....|....|....|....|....|....|....|**

**tRNA genes -->>>>>>>>>>>>>>>>>>>>>>>>>>>>>>>>>>>>>>>>>>>>>>>>>>>>>>>>>>>>>>>>>>>>>>>>>>------------------------**

**Col-0 ----------------------------------------------------------------------------------------------------**

**Cvi ----------------------------------------------------------------------------------------------------**

**A. suecica ----------------------------------------------------------------------------------------------------**

**A. arenosa AAGGGTCTGGTCCTTAGCTCAGAGGTAAAGCAAATTACTGCAGACCAATAAGTCACCGGGGACTCATTACAGCCCTTAAGACTCTCTTTTTTTTCCTCCC**

**A. lyrata AAGG-----TTCCTTAGCTCAGAGGTAAAGCAAATTACTGCAGATCAATAGGTCACCGCAGACTCATTACAGCCCTTAAGACTCTCTTTTTTTTCCACCC**

**A. halleri ---------------------------------------------------NNNNNNNNNNACTCGTTAAAGCCATTAAGACACTCTTTTTTTTCCTCCC**

**610 620 630 640 650 660 670 680 690 700**

**....|....|....|....|....|....|....|....|....|....|....|....|....|....|....|....|....|....|....|....|**

**tRNA genes ----------------------------------------------------------------------------------------------------**

**Col-0 ----------------------------------------------------------------------------------------------------**

**Cvi ----------------------------------------------------------------------------------------------------**

**A. suecica ----------------------------------------------------------------------------------------------------**

**A. arenosa tCCCAATCACTAATATGCCCATTTCACAAATCCCTGTGGAGTTTAGATCAATGACTGACTATTCTATAATCTTGTTTATGGGAAACCCATTAATAAACAA**

**A. lyrata TCCCAATCACTAATATCCCATTTTCACAAATTCCTGTGGAGTTTAGATCAATGACTGACTATTCTGCAATCCTGTTTATGGGAAACTCATTAATAAACAA**

**A. halleri TCCCAATCACTAATATCCCATTCTCACCAATCCCTGTGGAGTTTAGATCAATGACTGCCTATTCTACAATCCTGTTTATGGGTAACTCATTAATAAACAA**

**710 720 730 740 750 760 770 780 790 800**

**....|....|....|....|....|....|....|....|....|....|....|....|....|....|....|....|....|....|....|....|**

**tRNA genes ----->>>>>>>>>>>>>>>>>>>>>>>>>>>>>>>>>>>>>>>>>>>>>>>>>>>>>>>>>>>>>>>>>>>>>>>>-----------------------**

**Col-0 ----------------------------------------------------------------------------------------------------**

**Cvi ----------------------------------------------------------------------------------------------------**

**A. suecica ----------------------------------------------------------------------------------------------------**

**A. arenosa CCAAAGGGTCCTTAGCTCAGTGGTAGAGCAATTGACTGCAGATCAATAGGTCACTGGTTCGAATCCGGTAGGGCCCTTAATTTTTGTTAATTTTTTCCTT**

**A. lyrata CCAAAGGGTCCTTAGCTCAGTGGTAGAGCAATTGACTGCAGATCAATAGGCCACCGGTTCGAATCCGGTAGGGCCCTTAATTTTTGTTTATTTTTTCCTT**

**A. halleri CCAAAGGGTCCTTAGCTCAGTGGTAGAGCAATTGACTGCAGATCAATAGGCCACCGGTTCGAATCCGGTAGGGCCCTTAATTTTTGTTTATTTTTTCCTT**

**810 820 830 840 850 860 870 880 890 900**

**....|....|....|....|....|....|....|....|....|....|....|....|....|....|....|....|....|....|....|....|**

**tRNA genes ----------------------------------------------------------------------------------------------------**

**Col-0 -------------------------------------------------------------------------------------AAGG---GCTCAAGG**

**Cvi -------------------------------------------------------------------------------------AAGG---GCTCAAGG**

**A. suecica -------------------------------------------------------------------------------------AAGG---GCTCAAGG**

**A. arenosa ATTCCTAATCCTTTTTCTACAAATCCTTCAATTCCTTGATGTGT--------AAAAAGTGAAAACACCACTCAAAAATCAAAATAAAGGCAAGCTCAAAG**

**A. lyrata CTTCCTAATCCCTTTTCTACAAATCCTTTAATTCTTTGATATGTAAAAACTAAAAAAGTGAAAACACCACTCAAAAATCAAAATCAAGCCAAGCTCAAAG**

**A. halleri CTTCCTAATCCCTTTTCTACAAATCCTTCAATTCTTTGACGTGT-AAAAATTAAAAAGTGAAAACACCACTCAAAAATCAAAATCAAGGCAAGCTCAAAG**

**910 920 930 940 950 960 970 980 990 1000**

**....|....|....|....|....|....|....|....|....|....|....|....|....|....|....|....|....|....|....|....|**

**tRNA genes ----------------------------------------------------------------------------------------------------**

**Col-0 AATAAGT-AACAATTACCTTTTCTACCACCATAATGAGGAGCAGTGTCCCAAAACTCTTCTCTAAGTTTCATAAGCTCAGCTTTCGTGATCGGTTGGGTA**

**Cvi AATAAGT-AACAATTACCTTTTCTACCACCATAATGAGGAGCAGTGTCCCAAAACTCTTCTCTAAGTTTCATAAGCTCAGCTTTCGTGATCGGTTGGGTA**

**A. suecica AATAAGT-AACAATTACCTTTTCTACCACCATAATGAGGAGCAGTGTCCCAAAACTCTTCTCTAAGTTTCATAAGCTCAGCTTTCGTGATCGGTTGGGTA**

**A. arenosa AATAAGT-AACAATTACCTTTTCTACCGCCATAGTGAGGAGCAGTGTCCCAAAACTCTTCTCTAAGTTTCATAAGCTCAGCTTTCGTGATCGGTTGGGTA**

**A. lyrata AATAAGTAAAAAATTACCTTTTCTACCGCCATAGTGAGGAGCAGTGTCCCAAAACTCTTCTCTAAGTTTCATAAGCTCAGCTTTCGTGATCGGTTGGGTA**

**A. halleri AATAAGG-AACAATCACCTTTTCTACCGCCATAGTGAGGAGCAGTGTCCCAAAACTCTTCTCTAAGTTTCATAAGCTCAGCTTTCGTGATTGGTTGGGTA**

**Supplementary Figure S7.** Picture of the full-length gel presented in Figure 3C: PCR-based analysis of the size heterogeneity of the chromosome 5 tRNA-Cys clusters (see main text for details).


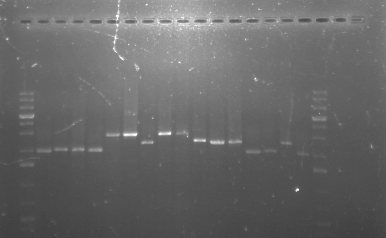


**Supplementary Figure S8**. Picture of the full-length gel presented in Figure 4D: experimental evaluation of the chromosome 1 tRNA-Cys cluster region from various A. thaliana accessions (see main text for details).


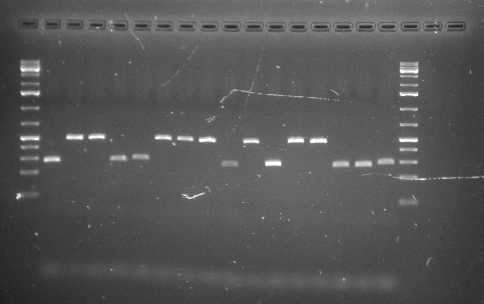


**Supplementary Figure S9.** Multiple sequence alignment of chromosome 5 cluster repeats used to construct the tree in Supplementary Figure S1.

R1.Kyo TATAATTTTTACAGTTT-ATCAACAATTTTTTATTAGAGCTTTATTTATACATTTGATTA

R2.Kyo --ACATTTTTTCAGTTT-ACCAATATTTTTTTATAAGAGCTTTAGTTATATATTTGATTA

R3.Kyo --ATATTTTTTCAGTTT-ACCAATAATTTTTAATAAGAGCTTTAGTTATATATTTGATTA

R4.Kyo --ATATCTTT-CAGTTT-ACCAATA-TTTTTTATAAGAGCTTTATTAATATATTTGATTA

R1.Eri-1 ----------------T-ATAAACAATTTTTTATTAGAGCTTTATTTATACATTTGATTA

R2.Eri-1 --ATATTTTTTCAGTTT-ACCAATATTTTTTTATAAGAGCTTTAGTTATATATTTGATTA

R3.Eri-1 --ATATTTTTTCAGTTT-ACCAATAATTTTTAATAAGAGCTTTAGTTATATATTTGATTA

R4.Eri-1 --ATATTTTT-CAGTTT-ACCAATA-TTTTTTATAAGAGTTTTATTAATATATTTGATTA

R1.Ler TATAATTTGTACAGTTT-ATCAACAATTTTTTATTAGAGCTTTATTTATACATTTGATTA

R2.Ler --ACATTTTTTCAGTTT-ACCAATATTTTTTTATAAGAGCTTTAGTTATATATTTGATTA

R3.Ler --ATATTTTT-CAATTT-ACCAATAATTTTTTATAAGAGCTTTAGTTATATATTTGATTA

R4.Ler --ATATTTTTTCAGTTT-ACCAATAATTTTTAATAAGAGCTTTAGTTATATATTTGATTA

R5.Ler --ATATTTTT-CAGTTT-ACCAATA-TTTTTTATAAGAGCTTTATTAATATATTTGATTA

R1.C24 TATAATTTTTACAGTTT-ATCAACAATTTTTTATTAGAGCTTTATTTATACATTTGATTA

R2.C24 --ATATTTTTTCAGTTT-ACCAATATTTTTTTATAAGAGCTTTAGTTATATATTTGATTA

R3.C24 --ATATTTTTTCAATTT-ACCAATAATTTTTTATAAGAGCTTTAGTTATATATTTGATTA

R4.C24 --ATATGTTTTCAGTTT-ACCAATAATTTTTTTTAAGAGTTTTAGTTATATATTTGATTA

R5.C24 --ATATTTTTTCAGTTT-ACCAATAATTTTTAATAAGAGCTTTAGTTATATATTTGATTA

R6.C24 --ATATTTTT-CAGTTT-ACCAATA-TTTTTTATAAGAGTTTTATTAATATATTTGATTA

R1.Tos82-387 TATAATTTTTACAGTTT-ATCAACAATTTTTTATTAGAGCTTTATTTATACATTTGATTA

R2.Tos82-387 --ATATTTTTTCAGTTT-ACCAATATTTTTTTATAAAAGCTTTAGTTATATATTTGATTA

R3.Tos82-387 --ATATTTTTTCAATTT-ACCAATAATTTTTTATAAGAGCTTTAGTTATATATTTGATTA

R4.Tos82-387 --ATATGTTTTCAGTTT-ACCAATAATTTTTTATAAGAGTTTTAGTTATATATTTGATTA

R5.Tos82-387 --ATATTTTTTCAGTTT-ACCAATAATTTTTAATAAGAGCTTTAGTTATATATTTGATTA

R6.Tos82-387 --ATATTTTT-TAGTTT-ACCAATA-TTTTTTATAAGAGTTTTATTAATATATTTGATTA

R1.An-1 TATCATTTTTACAGTTT-ATCAACAATTTTTTATTAGAGCTTTATTTATACATTTGATTA

R2.An-1 --ATATTTTTTCAGTTT-ACCAATATTTTTTTATAAGAGCTTTAGTTATATATTTGATTA

R3.An-1 --ATATTTTTTCAATTT-ACCAATAATTTTTTATAAGAGCTTTAGTTATATATTTGATTA

R4.An-1 --ATATGTTTTCAGTTT-ACCAATAATTTTTTATAAGAGTTTTAGTTATATATTTGATTA

R5.An-1 --ATATTTTTTCAGTTT-ACCAATAATTTTTAATAAGAGCTTTAGTTATATGTTTGATTA

R6.An-1 --ATATTTTT-CAGTTT-ACCAATA-TTTTTTATAAGAGTTTTATTAATATATTTGATTA

R1.KBS-Mac-74 TATCATTTTTACAGTTT-ATCAACAATTTTTTATTAGAGCTTTATTTATACATTTGATTA

R2.KBS-Mac-74 --ATATTTTTTCAGTTT-ACCAATATTTTTTTATAAGAGCTTTAGTTATATATTTGATTA

R3.KBS-Mac-74 --ATATTTTTTCAATTT-ACCAATAATTTTTTATAAGAGCTTTAGTTATATATTTGATTA

R4.KBS-Mac-74 --ATATGTTTTCAGTTT-ACCAATAATTTTTTATAAGAGTTTTAGTTATATATTTGATTA

R5.KBS-Mac-74 --ATATTTTTTCAGTTT-ACCAATAATTTTTAATAAGAGCTTTAGTTATATGTTTGATTA

R6.KBS-Mac-74 --ATATTTTT-CAGTTT-ACCAATA-TTTTTTATAAGAGTTTTATTAATATATTTGATTA

R1.Cdm-0 TATAATTTTTACAGTTT-ATCAACAATTTTTTATTAGAGCTTTATTTATACATTTGATTA

R2.Cdm-0 --ACATTTTTTCAGTTT-ACCAATATTTTTTTATAAGAGCTTTAGTTATATATTTGATTA

R3.Cdm-0 --ATATTTTT-CAATTT-ACCAATAATTTTTTATAAGAGCTTTAGTTATATATTTGATTA

R4.Cdm-0 --ATATTTTTTCAGTTT-ACCAATAATTTTTAATAAGAGCTTTAGTTATATATTTGATTA

R5.Cdm-0 --ATATTTTT-CAGTTT-ACCAATA-TTTTTTATAAGAGTTTTATTAATATATTTGATTA

R1.Kn-0 TATAATTTTTACAGTTT-ATCAACAATTTTTTATTAGAGCTTTATTTATACATTTGATTA

R2.Kn-0 --ACATTTTTTCAGTTT-ACCAATATTTTTTTATAAGAGCTTTAGTTATATATTTGATTA

R3.Kn-0 --ATATTTTT-CAATTT-ACCAATAATTTTTTATAAGAGCTTTAGTTATATATTTGATTA

R4.Kn-0 --ATATTTTTTCAGTTT-ACCAATAATTTTTAATAAGAGCTTTAGTTATATATTTGATTA

R5.Kn-0 --ATATTTTT-CAGTTT-ACCAATA-TTTTTTATAAGAGCTTTATTAATATATTTGATTA

R1.Cvi TATAATTTTTACAGTTT-ATCAACAATTTTTTATTAGAGCTTTATTTATACATTTGATTA

R2.Cvi --ATATTTTTTCAGTTT-ACCAATATTTTTTTATAAGAGCTTTAGTTATATATTTGATTA

R3.Cvi --ATATGTTTTCAGTTT-ACCAATAATTTTTTGTAAGAGTTTTAGTTATATATTTGATTA

R4.Cvi --ATATTTTTTCAGTTT-ACCAATAATTTTTAATAAGAGCTTTAGTTATATATTTGATTA

R5.Cvi ----ATTTTT-CAGTTT-ACCAATA-TTTTTTATAAGAGTTTTATTAATATATTTGATTA

R1.Fja-2-4 TATAATTTTTACAGTTT-ATCAACAATTTTTTATTAGAGCTTTATTTATACATTTGATTA

R2.Fja-2-4 --ACATTTTTTCAGTTT-ACCAATATTTTTTTATAAGAGCTTTAGTTATATATTTGATTA

R3.Fja-2-4 --ATATTTTTTCAGTTT-ACCAATAATTTTTAATAAGAGCTTTAGTTATATATTTGATTA

R4.Fja-2-4 --ATATCTTT-CAGTTT-ACCAATA-TTTTTTATAAGAGCTTTATTAATATATTTGATTA

R5.Fja-2-4 --ATATTTTT-CAGTTT-ACCAATA-TTTTTTATAAGAGCTTTATTAATATATTTGATTA

R1.Kor3 TATAATTTTTACAGTTT-ATCAACAATTTTTTATTAGAGCTTTATTTATACATTTGATTA

R2.Kor3 --ACATTTTTTCAGTTT-ACCAATATTTTTTTATAAGAGCTTTAGTTATATATTTGATTA

R3.Kor3 --ATATTTTTTCAGTTT-ACCAATAATTTTTAATAAGAGCTTTAGTTATATATTTGATTA

R4.Kor3 --ATATCTTT-CAGTTT-ACCAATA-TTTTTTATAAGAGCTTTATTAATATATTTGATTA

R1.Ty-1 TATAATTTTTACAGTTT-ATCAACAATTTTTTATTAGAGCTTTATTTATACAGTTGATTA

R2.Ty-1 --ATATTTTTTCAGTTT-ACCAATATTTTTTTATAAGAGCTTTAGTTATATATTTGATTA

R3.Ty-1 --ATATTTGTTCAATTT-ACCAATAGTTTTTTATAAGAGCTTTAGTTATATATTTGATTA

R4.Ty-1 --ATATGTTTTCAGTTT-ATCAATAATTTTTTATAAGAATTTTAGTTATATATTTGATTA

R1.Col-0 TATAATTTTTACAGTTT-ATCAACAATTTTTTATTAGAGCTTTATTTATACATTTGATTA

R2.Col-0 --ATATTTTTTCAGTTT-ACCAATATTTTTTTATAAGAGCTTTAGTTATATATTTGATTA

R3.Col-0 --ATATTTTTTCAATTT-ACCAATAATTTTTTAGAAGAGCTTTAGTTATATATTTGATTA

R4.Col-0 --ATATGTTTTCAGTTT-ACCAATAATTTTTTATAAGAGTTTTAGTTATATATTTGATTA

R1.Fly-2-2 TATAATTTTTACAGTTT-ATCAACAATTCTTTATTAGAGCTTTATTTATACATTTGATTA

R2.Fly-2-2 --ATATTTTTTCAGTTTTACCAATATTTTTTTATAAGAGCTTTAGTTATATATTTGATTA

R3.Fly-2-2 --ATATTTTTTCAATTT-ACCAATAATTTTTTATAAGAGCTTTAGTTATATATTTGATTA

R4.Fly-2-2 --ATATGTTTTCAGTTT-ACCAATAATTTTTTATAAGAGTTTTAGTTATATATTTGATTA

R1.Tur4 TATAATTTTTACAGTTT-ATCAACAATTCTTTATTAGAGCTTTATTTATACATTTGATTA

R2.Tur4 --ATATTTTTTCAGTTTTACCAATATTTTTTTATAAGAGCTTTAGTTATATATTTGATTA

R3.Tur4 --ATATTTTTTCAATTT-ACCAATAATTTTTTATAAGAGCTTTAGTTATATATTTGATTA

R4.Tur4 --ATATGTTTTCAGTTT-ACCAATAATTTTTTATAAGAGTTTTAGTTATATATTTGATTA

R1.Kyo GCTTTTAGCATCTAGTAGCTAACTAATCTTATATTTCAAGGTTGTAGCTCGCAATTTTTC

R2.Kyo GCATTTAGCGTCAAGTAGTTGACTAATCTTATATTTCAACGTTTTAGCTAGCAATTTTTC

R3.Kyo GCATTTAGCGTCAAGTAGTTGACTAATCTTATATTTCAAGGTTTTAGTTAGTAATTTTTC

R4.Kyo ACATTTAGCGTCTACTAGTTGACTAATTTTATATTATAAAGTTTTTTTTCGCAATTTTTC

R1.Eri-1 GCTTTTAGCATCTAGTAGCTAACTAATCTTATATTTCAAGGTTGTAGCTCGCAATTTTTC

R2.Eri-1 GCATTTAGCGTCAAGTAGTTGACTAATCTTATATTTCAAGGTTTTAGCTAGCAAATTTTC

R3.Eri-1 GCATTTAGCGTCAAGTAGTTGACTAATCTTATATTTCAAGGTTTTAGTTAGTAATTTTTC

R4.Eri-1 ACATTTAGCGTCTACTAGTTGACTAATTTTATATTATAAGGTTTTTTCTCGCAATTTTTC

R1.Ler GCTTTTAGCATCTAGTAGCTAACTAATCTTATATTTCAAGGTTGTAGCTCGCAATTTTTC

R2.Ler GCATTTAGCGTCAAGTAGTTGACTAATCTTATATTTCAAGGTTTTAGCTAGCGATTTTTC

R3.Ler ACATTTAGCATCAAGTAGTTGACTAATCTTATATTTCAACGTTTTAGCTAGCAATTTTTC

R4.Ler GCATTTAGCGTCAAGTAGTTGACTAATCTTATATTTCAAGGTTTTAGTTAGTAATTTTTC

R5.Ler ACATTTAGCGTCTACTAGTTGACTAATTTTATATTATAAGGTTTTTTCTCGCAATTTTTC

R1.C24 GCTTTTAGCATCTAGTAGCTAACTAATCTTATATTTCAAGGTTGTACCTCGCAATTTTTC

R2.C24 GCATTTAGCGTCAAGTAGTTGACTAATCTTATATTTCAAGGTTTTAGCTAGCAATTTTTC

R3.C24 GCATTTAGCATCAAGTAGTTGACTAATCTTATATTTCAACGTTTTAGGTAGCAATTTTTC

R4.C24 GCATTTAGCGTCAAGTAGTTCACTAATCTTATATTTCAAGGTTTTAGCTAGCAATTTTTC

R5.C24 GCATTTAGCGTCAAGTAGTTGACTAATCTTATATTTCAAGGTTTTAGTTAGTAATTTTTC

R6.C24 ACATTTAGCGTCTACTAGTTGACTAATTTTATATTATAAGGTTTTTTCTCGCAATTTTTC

R1.Tos82-387 GCTTTTAGCATCTAGTAGCTAACTAATCTTATATTTCAAGGTTGTAGCTCGCAATTTTTC

R2.Tos82-387 GCATTTAGCGTCAAGTAGTTGACTAATCTTATATTTCAAGGTTTTAGCTAGCAATTTTTC

R3.Tos82-387 GCATTTAGCATCAAGTAGTTGACTAATCTTATATTTCAACGTTTTAGCTAGCAATTTTTC

R4.Tos82-387 GCATTTAGCGTCAAGTAGTTCACTAATCTTATATTTCAAGGTTTTAGCTAGCAATTTTTC

R5.Tos82-387 GCATTTAGCGTCAAGTAGTTGACTAATCTTATATTTCAAGGTTTTAGTTAGTAATTTTTC

R6.Tos82-387 ACATTTAGCGTCTACTAGTTGACTAATTTTATATTATAAGGTTTTTTCTCGCAATTTTTC

R1.An-1 GCTTTTAGCATCTAGTAGCTAACTAATCTTATATTTCAAGGTTGTAGCTCGCAATTTTTC

R2.An-1 GCATTTAGCGTCAAGTAGTTGACTAATCTTATATTTCAAGGTTTTAGCTAGCAATTTTTT

R3.An-1 GCATTTAGCATCAAGTAGTTGACTAATCTTATATTTCAACGTTTTAGCTACCAATTTTTC

R4.An-1 GCATTTAGGGTCAAGTAGTTCACTAATCTTATACTTCAAGGTTTTAGCTAGCAATTTT-C

R5.An-1 GCATTTAGCGTCAAGTAGTTGACTAATCTTATATTTCAAGGTTTTAGCTAGTAATTTTTC

R6.An-1 ACATTTAGCGTCTACTAGTTGACTAATTTTATATTATAAGGTTTTTTCTCGCAATTTTTC

R1.KBS-Mac-74 GCTTTTAGCATCTAGTAGCTAACTAATCTTATATTTCAAGGTTGTAGCTCGCAATTTTTC

R2.KBS-Mac-74 GCATTTAGCGTCAAGTAGTTGACTAATCTTATATTTCAAGGTTTTAGCTAGCAATTTTTT

R3.KBS-Mac-74 GCATTTAGCATCAAGTAGTTGACTAATCTTATATTTCAACGTTTTAGCTAGCAATTTTTC

R4.KBS-Mac-74 GCATTTAGGGTCAAGTAGTTCACTAATCTTATACTTCAAGGTTTTAGCTAGCAATTTT-C

R5.KBS-Mac-74 GCATTTAGCGTCAAGTAGTTGACTAATCTTATATTTCAAGGTTTTAGCTAGTAATTTTTC

R6.KBS-Mac-74 ACATTTAGCGTCTACTAGTTGACTAATTTTATATTATAAGGTTTTTTCTCGCAATTTTTC

R1.Cdm-0 GCTTTTAGCATCTAGTAGCTAACTAATCTTATATTTCAAGGTTGTAGCTCGCAATTTTTC

R2.Cdm-0 GCATTTAGCGTCAAGTAGTTGACTAATCTTATATTTCAAGGTTTTAGCTAGCGATTTTTC

R3.Cdm-0 ACATTTAGCATCAAGTAGTTGACTAATCTTATATTTCAACGTTTTAGCTAGCAATTTTTC

R4.Cdm-0 GCATTTAGCGTCAAGTAGTTGACTAATCTTATATTTCAAGGTTTTAGTTAGTAATTTTTC

R5.Cdm-0 ACATTTAGCGTCTACTAGTTGACTAATTTTATATTATAAGGTTTTTTCTCGCAATTTTTC

R1.Kn-0 GCTTTTAGCATCTAGTAGCTAACTAATCTTATATTTCAAGGTTGTAGCTCGCAATTTTTC

R2.Kn-0 GCATTTAGCGTCAAGTAGTTGACTAATCTTATATTTCAAGGTTTTAGCTAGCAATTTTTC

R3.Kn-0 GCATTTAGCATCAAGTAGTTGACTAATCTTATATTTCAACGTTTTAGCTAGCAATTTTTC

R4.Kn-0 GCATTTAGCGTCAAGTAGTTGACTAATCTTATATTTCAAGGTTTTAGTTAGTAATTTTTC

R5.Kn-0 ACATTTAGCGTCTACTAGTTGACTAATTTTATATTATAAAGTTTTTTCTCGCAATTTTTC

R1.Cvi GCTTTTAGCAACTAGTAGCTAACTAATCTTATATTTCAAGGTTGTAGCTCGCAATTTTTC

R2.Cvi GCATTTAGCGTCAAGTAGTTGACTAATCTTATATTTCAAGGTTTTAGCTAGCAATTTTTC

R3.Cvi GCATTTAGCGTCAAGTAGTTCACTAATCTTATATTTCAAGGTTTTAGCTAGCAATTTTTC

R4.Cvi GCATTTAGCGTCAAGTAGTTGACTAATCTTATATTTCAAGGTTTTAGCTAGTAATTTTTC

R5.Cvi ACATTTAGCGTCTATTAGTTGACTAATTTTATATTATAAGGTTTTTTGTCGCAATTTTTC

R1.Fja-2-4 GCTTTTAGCATCTAGTAGCTAACTAATCTTATATTTCAAGGTTGTAGCTCGCAATTTTTC

R2.Fja-2-4 GCATTTAGCGTCAAGTAGTTGACTAATCTTATATTTCAACGTTTTAGCTAGCAATTTTTC

R3.Fja-2-4 GCATTTAGCGTCAAGTAGTTGACTAATCTTATATTTCAAGGTTTTAGTTAGTAATTTTTC

R4.Fja-2-4 ACATTTAGCGTCTACTAGTTGACTAATTTTATATTATAAAGTTTTTTTTCGCAATTTTTC

R5.Fja-2-4 ACATTTAGCGTCTACTAGTTGACTAATTTTATATTATAAAGTTTTTTCTCGCAATTTTTC

R1.Kor3 GCTTTTAGCATCTAGTAGCTAACTAATCTTATATTTCAAGGTTGTAGCTCGCAATTTTTC

R2.Kor3 GCATTTAGCGTCAAGTAGTTGACTAATCTTATATTTCAACGTTTTAGCTAGCAATTTTTC

R3.Kor3 GCATTTAGCGTCAAGTAGTTGACTAATCTTATATTTCAAGGTTTTAGTTAGTAATTTTTC

R4.Kor3 ACATTTAGCGTCTACTAGTTGACTAATTTTATATTATAAAGTTTTTTTTCGCAATTTTTC

R1.Ty-1 GCTTTTAGCAACTAGTAGCTAACTAATCTTATATTTCAAGGTTCTAGCTCGCAATTTTTC

R2.Ty-1 GCATTTAGCGTCAAGTAGTTGGCTAATCTTATATTTCAAGGTTTTAGCTAGCAATTTTTC

R3.Ty-1 GCATTTAGCATCAAGTAGTTGAATAATCTTATATTTCAACGTTTTAGCTAGCAATTTTTC

R4.Ty-1 GCATTTAGCGTCAAGTAGTTCACTAATCTTATATTTCAAGGTTTTAGCTAGCAATTTTTC

R1.Col-0 GCTTTTAGCAACTAGTAGCTAACTAATCTTATATTTCAAGGTTCTAGCTCGCAATATTTC

R2.Col-0 GCATTTAGCGTCAAGTAGTTGGCTAATCTTATATTTCAAGGTTTTAGCTAGCAATTTTTC

R3.Col-0 GCATTTAGCATCAAGTAGTTGACTAATCTTATATTTCAACGTTTTAGCTAGCAATTTTTC

R4.Col-0 GCATTTAGCGTCAAGTAGTTCACTAATCTTATATTTCAAGGTTTTAGCTAGCAATTTTTC

R1.Fly-2-2 GCTTTTAGCAACTAGTAGCTAACTAATCTTATATTTCAAGGTTGTAGCTCGCAATTTTTC

R2.Fly-2-2 GCATTTAGCGTCAAGTAGTTGACTAATCTTATATTTCAAGGTTTTAGCTAGCAATTTTTC

R3.Fly-2-2 GCATTTAGCATCAAGTAGTTGACTAATCTTATATTTCAACGTTTTAGCTAGCAATTTTTC

R4.Fly-2-2 GCATTTAGCGTCAAGTAGTTCACTAATCTTATATTTCAAGGTTTTAGCTAGCAATTTTTC

R1.Tur4 GCTTTTAGCAACTAGTAGCTAACTAATCTTATATTTCAAGGTTGTAGCTCGCAATTTTTC

R2.Tur4 GCATTTAGCGTCAAGTAGTTGACTAATCTTATATTTCAAGGTTTTAGCTAGCAATTTTTC

R3.Tur4 GCATTTAGCATCAAGTAGTTGACTAATCTTATATTTCAACGTTTTAGCTAGCAATTTTTC

R4.Tur4 GCATTTAGCGTCAAGTAGTTCACTAATCTTATATTTCAAGGTTTTAGCTAGCAATTTTTC

R1.Kyo ATTTTTAATAAAACAT-CTTTGACAAGTTTTT-GTATATAATTCTTTAGCAAGTGAATAT

R2.Kyo ATCTTGAATAAAACAAATTTTGACCAGTTTTT-GTATATAATTCTTTAGTAACTGATTTT

R3.Kyo ATCTTGAATAAAATAAATTTTGACAAGTTTTT-GTATATAATTCTTGAGCAACTGAAAAT

R4.Kyo ATCTTGAATAAAA-ACATTTTGACAAGTTTTT-TAATATAATTCATTAGCAACTGAAAAT

R1.Eri-1 ATTTTTAATAAAACAT-C------AAGTTTTT-GTATATAATTCTTTAGCAAGTGAATAT

R2.Eri-1 ATCCTGAATAAAACAAATTTTGACCAGTTTTT-ATATATAATTCTTTAGCAACTGAATTT

R3.Eri-1 ATCTTGAATAAAATAAATTTTGACAAGTTTTT-GTATATAATTCTTGAGCAACTGAAAAT

R4.Eri-1 ATCTTGAATAAAA-ACATTTTGACAAGTTTTT-TAATATAATTCATTAGCAACTGAAAAT

R1.Ler ATTTTTAATAAAACAT-CTTTGACAAGTTTTT-GTATATAATTCTTTAGCAAGTGAATAT

R2.Ler ATCCTGAATAAAACAAATTTTGACCAGTTTTT-ATATCTAATTCTTTAGCAACTGAATTT

R3.Ler ATCTTGAATAAAACAAATTTTGACCAGTTTTT-GTATATAATTCTTTAGTAACTGATTTT

R4.Ler ATCTTGAATAAAATAAATTTTGACAAGTTTTT-GTATATAATTCTTGAGCAACTGAAAAT

R5.Ler ATCTTGAATAAAA-ACATTTTGACAAGTTTTT-TAATATAATTCATTAGCAACTGAAAAT

R1.C24 ATTTTTAATAAAACAT-CTTTGACAAGTTTTT-GTATATAATTCTTTAGCAAGTGAATAT

R2.C24 ATCCTGAATAAAACAAATTTTGACCAGTTTTT-ATATATAATTCTTTAGCAACTGAATTT

R3.C24 ATCTTGAATAAAACAAATTTTGACCAGTTTTT-GTATATAATTCTTTAGTAACTGATTTT

R4.C24 ATCTTGAATAAAACACATTTTGACCTGTTTTT-GCATATAATTCTTTAACAACTGAATTT

R5.C24 ATCTTGAATAAAATAAATTTTGACAAGTTTTT-GTATATAATTCTTGAGCAACTGAAAAT

R6.C24 ATCTTGAATAAAA-ACATTTTGACAAGTTTTT-TAATATAATTCATTAGCAACTGAAAAT

R1.Tos82-387 ATTTTTAATAAAACAT-C------AAGTTTTT-GTATATAATTCTTTAGCAAGTGAATAT

R2.Tos82-387 ATCCTGAATAAAACAAATTTTGACCAGTTTTT-ATATATAATTCTTTAGCAACTGAATTT

R3.Tos82-387 ATCTTGAATAAAACAAATTTTGACTAGTTTTT-GTATATAATTCTTTAGTAACTGATTTT

R4.Tos82-387 ATCTTGAATAAAACAAATTTTGACCTGTTTTT-GCATATAATTCTTTAACAACTGATTTT

R5.Tos82-387 ATCTTGAATAAAATAAATTTTGACAAGTTTTT-GTATATAATTCTTGAGCAACTGAAAAT

R6.Tos82-387 ATCTTGAATAAAA-ACATTTTGACAAGTTTTT-TAATATAATTCATTAGCAACTGAAAAT

R1.An-1 ATTTTTAATAAAACAT-CTTTGACAAGTTTTT-GTATATAATTCTTTAGCAAGTGAATAT

R2.An-1 ATCCTGAATAAAACAAATTTTGACCAGTTTTT-ATATATAATTCTTTAACAACTGAATTT

R3.An-1 ATCTTGAATAAAACAAATTTTGACCAGTTTTT-GTATATAATTCTTTAGTAACTGAATTT

R4.An-1 ATCTTGAATAAAACAAATTTTGACCTGTTTTT-GCATATAATTCTTTAACAACTGAATTT

R5.An-1 ATCTTGAATAAAATAAATTTTGACAAGTTTTT-GTATATAATTCTTGAGCAACTGAAAAT

R6.An-1 ATCTTGAATAAAA-ACATTTTGACAAGTTTTT-TAATATAATTTATTAGCAATTGAAAAT

R1.KBS-Mac-74 ATTTTTAATAAAACAT-CTTTGACAAGTTTTT-GTATATAATTCTTTAGCAAGTGAATAT

R2.KBS-Mac-74 ATCCTGAATAAAACAAATTTTGACCAGTTTTT-ATATATAATTCTTTAACAACTGAATTT

R3.KBS-Mac-74 ATCTTGAATAAAACAAATTTTGACCAGTTTTT-GTATATAATTCTTTAGTAACTGAATTT

R4.KBS-Mac-74 ATCTTGAATAAAACAAATTTTGACCTGTTTTT-GCATATAATTCTTTAACAACTGAATTT

R5.KBS-Mac-74 ATCTTGAATAAAATAAATTTTGACAAGTTTTT-GTATATAATTCTTGAGCAACTGAAAAT

R6.KBS-Mac-74 ATCTTGAATAAAA-ACATTTTGACAAGTTTTT-TAATATAATTTATTAGCAATTGAAAAT

R1.Cdm-0 ATTTTTAATAAAACAT-CTTTGACAAGTTTTT-GTATATAATTCTTTAGCAAGTGAATAT

R2.Cdm-0 ATCCTGAATAAAACAAATTTTGACCAGTTTTT-ATATATAATTCTTTAGCAACTGAATTT

R3.Cdm-0 ATCTTGAATAAAACAAATTTTGACCAGTTTTT-GTATATAATTCTTTAGTAACTGATTTT

R4.Cdm-0 ATCTTGAATAAAATAAATTTTGACAAGTTTTT-GTATATAATTCTTGAGCAACTGAAAAT

R5.Cdm-0 ATCTTGAATAAAA-ACATTTTGACAAGTTTTT-TAATATAATTCATTAGCAACTGAAAAT

R1.Kn-0 ATTTTTAATAAAACAT-CTTTGACAAGTTTTT-GTATATAATTCTTTAGCAAGTGAATAT

R2.Kn-0 ATCCTGAATAAAACAAATTTTGACCAGTTTTT-ATATATAATTCTTTAGCAACTGAATTT

R3.Kn-0 ATCTTGAATAAAACAAATTTTGACCAGTTTTT-GTATATAATTCTTTAGTAACTGATTTT

R4.Kn-0 ATCTTGAATAAAATAAATTTTGACAAGTTTTT-GTATATAATTCTTGAGCAACTGAAAAT

R5.Kn-0 ATCTTGAATAAAA-ACATTTTGACAAGTTTTT-TAATATAATTCATTAGCAACTGAAAAT

R1.Cvi ATTTTTAATAAAACAT-CTTTGACAAGTTTTT-GTATATAATTCTTTAGCAAGTGAATAT

R2.Cvi ATCCTGAATAAAACATATTTTGACCAGTTTTT-ATATATAATTCTTTAGCAACTGATTTT

R3.Cvi ATCTTGAATAAAACAAATTTTGACCTGTTTTT-GCATATAATTCTTTAACAACTGAATTT

R4.Cvi AT---GAATAAAATAAATTTTGACAAGTTTTTTGTATATAATTCTTGAGCAACTGAAAAT

R5.Cvi ATCTTGAATAAAA-ACATTTTGACAAGTTTTT-TAATATAATTCATTAGCAACTGAAAAT

R1.Fja-2-4 ATTTTTAATAAAACAT-CTTTGACAAGTTTTT-GTATATAATTCTTTAGCAAGTGAATAT

R2.Fja-2-4 ATCTTGAATAAAACAAATTTTGACCAGTTTTT-GTATATAATTCTTTAGTAACTGATTTT

R3.Fja-2-4 ATCTTGAATAAAATAAATTTTGACAAGTTTTT-GTATATAATTCTTGAGCAACTGAAAAT

R4.Fja-2-4 ATCTTGAATAAAA-ACATTTTGACAAGTTTTT-TAATATAATTCATTAGCAACTGAAAAT

R5.Fja-2-4 ATCTTGAATAAAA-ACATTTTGACAAGTTTTT-TAATATAATTCATTAGCAACTGAAAAT

R1.Kor3 ATTTTTAATAAAACAT-CTTTGACAAGTTTTT-GTATATAATTCTTTAGCAAGTGAATAT

R2.Kor3 ATCTTGAATAAAACAAATTTTGACCAGTTTTT-GTATATAATTCTTTAGTAACTGATTTT

R3.Kor3 ATCTTGAATAAAATAAATTTTGACAAGTTTTT-GTATATAATTCTTGAGCAACTGAAAAT

R4.Kor3 ATCTTGAATAAAA-ACATTTTGACAAGTTTTT-TAATATAATTCATTAGCAACTGAAAAT

R1.Ty-1 ATTTTTAATAAAACAT-CTTTGACAAGTTTTT-GTATATAATTCTTTAGCAAGTGAATAT

R2.Ty-1 ATCCTGAATAAAACAAATTTTGACCAGTTTTT-ATATATAATTCTTTAGCAACTGAATTT

R3.Ty-1 ATCTTGAATAAAACAAATTTTGACCAGTTTTT-GTATATAATTCTTTAGTAACTGAATTT

R4.Ty-1 ATCTTGAATAAAACAAATTTTGACCTGTTTTT-GCATATAGTTCTTTAATAACTGAAATT

R1.Col-0 ATTTTTAATAAAACAT-CTTTGACAAGTTTTT-GTATATAATTCTTTAGCAAGTGAATAT

R2.Col-0 ATCCTGAATAAAACAAATTTTGACCAGTTTTT-ATATATAATTCTTTAGCAACTGAATTT

R3.Col-0 ATCTTGAATAAAACAAATTTTGATCAGTTTTT-GTATATAATTCTTTAGTAACTGAATTT

R4.Col-0 ATCTTGAATAAAACAAATTTTGACCTGTTTTT-GCATATAATTCTTTAACAACTGAAATT

R1.Fly-2-2 ATTTTTAATAAAACAT-CTTTGACAAGTTTTT-GTATATAATTCTTTAGCAAGTGAATAT

R2.Fly-2-2 ATCCTGAATAAAACAAATTTTGACCAGTTTTT-ATATATAATTCTTTAGCAACTGAATTT

R3.Fly-2-2 ATCTTGAATAAAACAAATTTTGACCAGTTTTT-GTATATAATTCTTTAGTAACTGAATTT

R4.Fly-2-2 ATTTTGAATAAAATAAATTTTGACCTGTTTTT-GCATATAATTCTTTAACAACTGAATTT

R1.Tur4 ATTTTTAATAAAACAT-CTTTGACAAGTTTTT-GTATATAATTCTTTAGCAAGTGAATAT

R2.Tur4 ATCCTGAATAAAACAAATTTTGACCAGTTTTT-ATATATAATTCTTTAGCAACTGAATTT

R3.Tur4 ATCTTGAATAAAACAAATTTTGACCAGTTTTT-GTATATAATTCTTTAGTAACTGAATTT

R4.Tur4 ATTTTGAATAAAATAAATTTTGACCTGTTTTT-GCATATAATTCTTTAACAACTGAATTT

R1.Kyo GTTTTT-CTTTATAATTTCAAGGTTTAATTTGTTTGTGAAAATTGTTTTT--GATAATTT

R2.Kyo TTTTAA--AAAAAAA-TTCATGGTTTAATTTGTTTGTGAATTTTTTTTTATAAATAATTT

R3.Kyo ATTTTT--TT-AAAATTTCATGGTTTAGTTTGTTTGTTA-TTTGTTTTATGAAATAATT-

R4.Kyo GTTTTT-TTTTT-CATTTCATGGTTTAATTTGTTTGTGAAAATTGTTTTT--GATAATTT

R1.Eri-1 GTTTTT-CTTTATAATTTCAAGGTTTAATTTGTTTGTGAAAATTGTTTTT--GATAATTT

R2.Eri-1 TTTTTA-AAAAAAAAATTCATGGTTTAATTTGTTTGTGAATATTTTTTA-AAAATAATTT

R3.Eri-1 ATTTTT--TT-AAAATTTCATGGTTTAATTTGTTTGTTA-TTTGTTTTATGAAATAATT-

R4.Eri-1 GTTTTT-TTTTT-CATTTCATGGTTTAATTTGTTTGTGAAAATTGTTTTT--GATAATTT

R1.Ler GTTTTT-CTTTATAATTTGAAGGTTTAATTTGTTTGTGAAAATTGTTTTT--GATAATTT

R2.Ler TTTTAA-A-AACAAAATTCATGGTTTAATTTGTTTGTGAATATTTTTT-AAAAATAATTT

R3.Ler TTTTAA--AAAAAAA-TTCATGGTTTAATTTGTTTATGAATTTTTTTTTATAAATAATTT

R4.Ler ATTTTT--TT-AAAATTTCATGGTTTAGTTTGTTTGTTA-TTTGTTTTATGAAATAATT-

R5.Ler GTTTTT-TTTTT-CATTTCATGGTTTAATTTGTTTGTGAAAATTGTTTTT--GATAATTT

R1.C24 GTTTTT-CTTTATAATTTCAAGGTTTAATTTGTTTGTGAAAATTGTTTTT--GATAATTT

R2.C24 TTTTAA-AAAAAAAAATTCATGGTTTAATTTGTTTGTGAATATTTTTTA-AAAATAATTT

R3.C24 TAA-AA--AAAAAAA-TTCATGGTTTAATTTGTTTGTGAATTTTGTTTTTTAAATCATTT

R4.C24 TTTTAA--AAAAACT-TTCATGGTTTAATTTGTTTGTGAATTTTTTTTTATAAATAATTT

R5.C24 ATTTTT--T--AAAATTTCATGGTTTAATTTGTTTGTTA-TTTGTTTTATGAAATAATT-

R6.C24 GTTTTT-TTTTT-CATTTCATGGTTTAATTTGTTTGTGAAAATTGTTTTT--GATAATTT

R1.Tos82-387 GTTTTT-CTTTATAATTTCAAGGTTTAATTTGTTTGTGAAAATTGTTTTT--GATAATTT

R2.Tos82-387 TTTTTT-TAAAAAAAATTCATGGTTTAATTTGTTTGTGAATATTTTTT-AAAAATAATTT

R3.Tos82-387 TTTTAA--AAAAAAAATTCATGGTTTAATTTGTTTGTGAATTTTGTTTTTTAAATAATTT

R4.Tos82-387 TTTTTA--AAAAACT-TTCATGGTTTAATTTGTTTGTGAATTTTTTTTTATAAATAATTT

R5.Tos82-387 ATTTTT--TT-AAAATTTCATGGTTTAATTTGTTTGTTA-TTTGTTTTATGAAATAATT-

R6.Tos82-387 GTTTTT-TTTTT-CATTTCATGGTTTAATTTGTTTGTGAAAATTGTTTTT--GATAATTT

R1.An-1 GTTTTT-CTTTATAATTTCAAGGTTTAATTTGTTTGTGAAAATTGTTTTT--GATAATTT

R2.An-1 TTTTAA-AAAAAAAAATTCATGGTTTAATTTGTTTGTGAATATTTTTTTTAAAATAATTT

R3.An-1 TTTTTA--AAAACAA-TTCATGGTTTAATTTGTTTGTGAATTTTGTTTTTTAAATAATTT

R4.An-1 TTTTTT--AAAAACT-TTCATGGTTTAATTTGTTTGTGAATTTTTTTTTATAAATAATTT

R5.An-1 ATTTTT--TT-AAAATTTCATGGTTTAATTTGTTTGTTA-TTTGTTTTATTAAATAATT-

R6.An-1 GTTTTT-TTTTT-CATTTCATGGTTTAATTTGTTTGTGAAAATTGTTTTT--GATAATTT

R1.KBS-Mac-74 GTTTTT-CTTTATAATTTCAAGGTTTAATTTGTTTGTGAAAATTGTTTTT--GATAATTT

R2.KBS-Mac-74 TTTTTA-AAAAAAAAATTCATGGTTTAATTTGTTTGTGAATATTTTTTTTAAAATAATTT

R3.KBS-Mac-74 TTTTTA--AAAACAA-TTCATGGTTTAATTTGTTTGTGAATTTTGTTTTTTAAATAATTT

R4.KBS-Mac-74 TTTTTT--AAAAACT-TTCATGGTTTAATTTGTTTGTGAATTTTTTTTTATAAATAATTT

R5.KBS-Mac-74 ATTTTT--TT-AAAATTTCATGGTTTAATTTGTTTGTTA-TTTGTTTTATTAAATAATT-

R6.KBS-Mac-74 GTTTTT-TTTTT-CATTTCATGGTTTAATTTGTTTGTGAAAATTGTTTTT--GATAATTT

R1.Cdm-0 GTTTTT-CTTTATAATTTGAAGGTTTAATTTGTTTGTGAAAATTGTTTTT--GATAATTT

R2.Cdm-0 TTTTAA-A-AACAAAATTCATGGTTTAATTTGTTTGTGAATATTTTTT-AAAAATAATTT

R3.Cdm-0 TTTTTA--AAAAAAA-TTCATGGTTTAATTTGTTTATGAATTTTTTTTTATAAATAATTT

R4.Cdm-0 ATTTTT--TT-AAAATTTCATGGTTTAGTTTGTTTGTTA-TTTGTTTTATGAAATAATT-

R5.Cdm-0 GTTTTT-TTTTT-CATTTCATGGTTTAATTTGTTTGTGAAAATTGTTTTT--GATAATTT

R1.Kn-0 GTTTTT-CTTTATAATTTCAAGGTTTAATTTGTTTGTGAAAATTGTTTTT--GATAATTT

R2.Kn-0 TTTTTT-T-AAAAAAATTCATGGTTTAATTTGTTTGTGAATATTTTTT-AAAAATAATTT

R3.Kn-0 TTTTAA--AAAAAAA-TTCATGGTTTAATTTGTTTGTGAATTTTTTTTTATAAATAATTT

R4.Kn-0 ATTTTT--TTTAAAATTTCATGGTTTAGTTTGTTTGTTA-TTTGTTTTATGAAATAATT-

R5.Kn-0 GTTTTT-TTTTTTCATTTCATGGTTTAATTTGTTTGTGAAAATTGTTTTT--GATAATTT

R1.Cvi GTTTTT-CTTTACAATTTCAAGGTTTAATTTGTTTGTGAAAATTGTTTTT--GATAATTT

R2.Cvi TTTTTT-TTTAAAAAATTCATGGTTTAATTTGTTTGTGAATATTTTTTAAAAAATAATTT

R3.Cvi TTTTTT--AAAAACT-TTCATGGTTTAATTTGTTTGTGAATTTTTTTTTATAAATAATTT

R4.Cvi ATTTTT--TT-AAAATTTCATGGTTTAATTTGTTTGTTA-TTTGTTTTATTAAATAATT-

R5.Cvi GTTTTT-TTTTT-CGTTTTATGGTTTAATTTGTTTGTGAAAATTGTTTTT--GATAATTT

R1.Fja-2-4 GTTTTT-CTTTATAATTTCAAGGTTTAATTTGTTTGTGAAAATTGTTTTT--GATAATTT

R2.Fja-2-4 TTTTAA--AAAAAAA-TTCATGGTTTAATTTGTTTGTGAATTTTTTTTTATAAATAATTT

R3.Fja-2-4 ATTTTT--TT-AAAATTTCATGGTTTAGTTTGTTTGTTA-TTTGTTTTATGAAATAATT-

R4.Fja-2-4 GTTTTT-TTTTT-CATTTCATGGTTTAATTTGTTTGTGAAAATTGTTTTT--GATAATTT

R5.Fja-2-4 GTTTTT-TTTTT-CATTTCATGGTTTAATTTGTTTGTGAAAATTGTTTTT--GATAATTT

R1.Kor3 GTTTTT-CTTTATAATTTCAAGGTTTAATTTGTTTGTGAAAATTGTTTTT--GATAATTT

R2.Kor3 TTTTAA--AAAAAAA-TTCATGGTTTAATTTGTTTGTGAATTTTTTTTTATAAATAATTT

R3.Kor3 ATTTTT--TT-AAAATTTCATGGTTTAGTTTGTTTGTTA-TTTGTTTTATGAAATAATT-

R4.Kor3 GTTTTT-TTTTT-CATTTCATGGTTTAATTTGTTTGTGAAAATTGTTTTT--GATAATTT

R1.Ty-1 GTTTTT-CTTTATAATTTCAAGGTTTAATTTGTTTGTGAAAATTGTTTTT--GATAATTT

R2.Ty-1 TTTTTTTAAAAAAAAATTCATGGTTTAATTTGTTTGTGAATATTTTTTTTAAAATAATTT

R3.Ty-1 TTTTAA--AAAAAAA-ATCATGGTTTAATTTGTTTGTGAATTTTGTTTTTTAAATAATTT

R4.Ty-1 TTTTTA--AAAAACT-TTCATGGTTTAATTTGTTTGTGAATTTTATTTTATAAATAATTT

R1.Col-0 GTTTTT-CTTTATAATTTCAAGGTTTAATTTGTTTGTGAAAATTGTTTTT--GATAATTT

R2.Col-0 TTTTTTTAAAAAAAAATTCATGGTTTAATTTGTTTGTGAATATTTTTTTTAAAATAATTT

R3.Col-0 TTTTTA--AAAAAAA-TTCATGGTTTAATTTGTTTGTGAATTTTGTTTTTTAAATAATTT

R4.Col-0 TTTTTA--AAAAACT-TTCATGGTTTAATTTGTTTGTGAATTTTTTTTTATAAATAATTT

R1.Fly-2-2 GTTTTT-CTTTATAATTTCAAGGTTTAATTTGTTTGTGAAAATTGTTTTT--GATAATTT

R2.Fly-2-2 TTTTTA-AAAAAAAAATTCATGGTTTAATTTGTTTGTGAATATTTTTTTTTAAATAATTT

R3.Fly-2-2 TTTTTA--AAAAAAA-TTCATGGTTTAATTTGTTTGTGAATTTTGTTTTTTAAATAATTT

R4.Fly-2-2 TTTTTA--AAAAACT-TTCATGGTTTAATTTGTTTGTGAATTTTTTTTTATAAATAATTT

R1.Tur4 GTTTTT-CTTTATAATTTCAAGGTTTAATTTGTTTGTGAAAATTGTTTTT--GATAATTT

R2.Tur4 TTTTTA-AAAAAAAAATTCATGGTTTAATTTGTTTGTGAATATTTTTTTTTAAATAATTT

R3.Tur4 TTTTTA--AAAAAAA-TTCATGGTTTAATTTGTTTGTGAATTTTGTTTTTTAAATAATTT

R4.Tur4 TTTTTT--AAAAACT-TTCATGGTTTAATTTGTTTGTGAATTTTTTTTTATAAATAATTT

R1.Kyo ATATGATTACTAAATAAGTAAACAATTGACTTGCTTATATTAGATTTCTTAGCAAAAAAA

R2.Kyo ATGTGATTTCTAAATAAGTAAACAATTGACTTAACTATATTAGATTTCTTAG-CAAAAAA

R3.Kyo ATGTGATTTCTAAATAAGTAAATAATTGA-----CTATATTAGATTTCTTAGCAAAAAAA

R4.Kyo ATATGATTACTAAATAAGTAAATAACTGACTTGGATATATTAGATTTTTTAG-CAAAAAA

R1.Eri-1 ATATGATTACTAAATAAGTAAACAATTGACTTGCTTATATTAGATTTCTTAGCAAAAAAA

R2.Eri-1 ATGTGATTTCTAAATAAGTAAACAATTGACTTAACTATATTAGATTTCTTAG-CAAAAAA

R3.Eri-1 ATGTGATTTCTAAATAAGTAAATAATTGA-----CTATATTAGATTTCTTAGCAAAAAAA

R4.Eri-1 ATATGATTACTAAATAAGTAAATAACTGACTTGGATATATTAGATTTTTTAG-CAAAAAA

R1.Ler ATATGATTACTAAATAAGTAAACAATTGACTTGCTTATATTAGATTTCTTAGCAAAAAAA

R2.Ler ATGTGATTTCTAAATAAGTAAACAATTGACTTAACTATATTAGATTTCTTAG-CAAAAAA

R3.Ler ATGTGATTTCTTAATAAGTAAACAATTGACTTAACTATATTAGATTTCTTAG-CAAAAAA

R4.Ler ATGTGATTTCTAAATAAGTAAATAATTGA-----CTATATTAGATTTTTTAGCAAAAAAA

R5.Ler ATATGATTACTAAATAAGTAAATAACTGACTTGGATATATTAGATTTCTTAG-CAAAAAA

R1.C24 ATATGATTACTAAATAAGTAAACAATTGACTTGCTTATATTAGATTTCTTAGCAAAAAAA

R2.C24 ATGTGATTTCTAAATAAGTAAACAATTGACTTAACTATATTAGATTTCTTAG-CAAAAAA

R3.C24 ATGTGATTTTTAAATAAGTAAACAATTGACTTAACTATATTAGATTTCTTAG-CAAAAAA

R4.C24 ATGTGATTTCTAAATAAGTAAACAATTGACTTAACTATATTAGATTTCTTAG-CAAAAAA

R5.C24 ATGTGATTTCTAAATAAGTAAATAATTGA-----CTATATTAGATTTCTTAGCAAAAAAA

R6.C24 ATATGATTACTAAATAAGTAAATAACTGACTTGGATATATTAGATTTCTTAG-CAAAAAA

R1.Tos82-387 ATATGATTACTAAATAAGTAAACAATTGACTTGCTTATATTAGATTTCTTAGCAAAAAAA

R2.Tos82-387 ATGTGATTTCTAAATAAGTAAACAATTGGCTTAACTATATTAGATTTCTTAG-CAAAAAA

R3.Tos82-387 ATGTGATTTTTAAATAAGTAAACAATTGACTTAACTATATTAGATTTCTTAG-CAAAAAA

R4.Tos82-387 ATGTGATTTCTAAATAAGTAAACAATTGACTTAACTATATTAGATTTCTTAG-CAAAAAA

R5.Tos82-387 ATGTGATTTCTAAATAAGTAAATAATTGA-----CTATATTAGATTTCTTAGCAAAAAAA

R6.Tos82-387 ATATGATTACTAAATAAGTAAATAACTGACTTGGATATATTAGATTTCTTAG-CAAAAAA

R1.An-1 ATATGATTACTAAATAAGTAAACAATTGACTTGCTTATATTAGATTTCTTAGCAAAAAAA

R2.An-1 ATGTGATTTCTAAATAAGTAAACAATTGACTTAACTATATTAGATTTCTTAG--AAAAAA

R3.An-1 ATATGATTTCTAAATAAGTAAACAATTGACTTAACTATATTAGATTTCTTAGCAAAAAAA

R4.An-1 ATGTGATTTCTAAATAAGTAAACAATTGACTTAACTATATTAGATTTCTTAG-CAAAAAA

R5.An-1 ATGTGATTTCTAAATAAGTAAATAATTGA-----CTATATTAGATTTCTTAGCAAAAAAA

R6.An-1 ATATGATTACTAAATAAGTAAATAACTGACTTGGATATATTAGATTTCTTAG-CAAAAAA

R1.KBS-Mac-74 ATATGATTACTAAATAAGTAAACAATTGACTTGCTTATATTAGATTTCTTAGCAAAAAAA

R2.KBS-Mac-74 ATGTGATTTCTAAATAAGTAAACAATTGACTTAACTATATTAGATTTCTTAG--AAAAAA

R3.KBS-Mac-74 ATATGATTTCTAAATAAGTAAACAATTGACTTAACTATATTAGATTTCTTAG-AAAAAAA

R4.KBS-Mac-74 ATGTGATTTCTAAATAAGTAAACAATTGACTTAACTATATTAGATTTCTTAG-CAAAAAA

R5.KBS-Mac-74 ATGTGATTTCTAAATAAGTAAATAATTGA-----CTATATTAGATTTCTTAGCAAAAAAA

R6.KBS-Mac-74 ATATGATTACTAAATAAGTAAATAACTGACTTGGATATATTAGATTTCTTAG-CAAAAAA

R1.Cdm-0 ATATGATTACTAAATAAGTAAACAATTGACTTGCTTATATTAGATTTCTTAGCAAAAAAA

R2.Cdm-0 ATGTGATTTCTAAATAAGTAAACAATTGACTTAACTATATTAGATTTCTTAG-CAAAAAA

R3.Cdm-0 ATGTGATTTCTTAATAAGTAAACAATTGACTTAACTATATTAGATTTCTTAG-CAAAAAA

R4.Cdm-0 ATGTGATTTCTAAATAAGTAAATAATTGA-----CTATATTAGATTTTTTAGCAAAAAAA

R5.Cdm-0 ATATGATTACTAAATAAGTAAATAACTGACTTGGATATATTAGATTTCTTAG-CAAAAAA

R1.Kn-0 ATATGATTACTAAATAAGTAAACAATTGACTTGCTTATATTAGATTTCTTAGCAAAAAAA

R2.Kn-0 ATGTGATTTCTAAATAAGTAAACAATTGACTTAACTATATTAGATTTCTTAG-CAAAAAA

R3.Kn-0 ATGTGATTTCTAAATAAGTAAACAATTGACTTAACTATATTAGATTTCTTAG-CAAAAAA

R4.Kn-0 ATGTGATTTCTAAATAAGTAAATAATTGA-----CTATATTAGATTTCTTAGCAAAAAAA

R5.Kn-0 ATATGATTACTAAATAAGTAAATAACTGACTTGGATATATTAGATTTCTTAG-CAAAAAA

R1.Cvi ATATGATTAGTAAATAAGTAAACAATTGACTTGCTTATATTAGATTTCTTAGCAAAAAAA

R2.Cvi ATGTGATTTCTAAATAAGTAAACAATTGACTTAACTATATTAGATTTCTTAG-CAAAAAA

R3.Cvi ATGTGATTTCTAAATAAGTAAACAATTGACTTAACTATATTAAATTTCTTAG-CAAAAAA

R4.Cvi ATGTGATTTCTAAATAAGTAAATAATTGA-----CTATATTAGAT---------------

R5.Cvi ATATGATTACTAAATAAATAAATAACTGACTTGGATATATTAGATTTCTTAG-CAAAAAA

R1.Fja-2-4 ATATGATTACTAAATAAGTAAACAATTGACTTGCTTATATTAGATTTCTTAGCAAAAAAA

R2.Fja-2-4 ATGTGATTTCTAAATAAGTAAACAATTGACTTAACTATATTAGATTTCTTAG-CAAAAAA

R3.Fja-2-4 ATGTGATTTCTAAATAAGTAAATAATTGA-----CTATATTAGATTTCTTAGCAAAAAAA

R4.Fja-2-4 ATATGATTACTAAATAAGTAAATAACTGACTTGGATATATTAGATTTTTTAG-CAAAAAA

R5.Fja-2-4 ATATGATTACTAAATAAGTAAATAACTGACTTGGATATATTAGATTTCTTAG-CAAAAAA

R1.Kor3 ATATGATTACTAAATAAGTAAACAATTGACTTGCTTATATTAGATTTCTTAGCAAAAAAA

R2.Kor3 ATGTGATTTCTAAATAAGTAAACAATTGACTTAACTATATTAGATTTCTTAG-CAAAAAA

R3.Kor3 ATGTGATTTCTAAATAAGTAAATAATTGA-----CTATATTAGATTTCTTAGCAAAAAAA

R4.Kor3 ATATGATTACTAAATAAGTAAATAACTGACTTGGATATATTAGATTTTTTAG-CAAAAAA

R1.Ty-1 ATATGATTACTAAATAAGTAAACAATTGACTTGCTTATATTAGATTTCTTAGCAAAAAAA

R2.Ty-1 ATGTGATTTCTAAATAAGTAAACAATTGACTTAAATATATTAGATTTCTTAG-CAAGAAA

R3.Ty-1 ATGTGATTTCTAAATAAGTAAACAATTGACTTAACTATATTAGATTTCTTAG-CAAAAAA

R4.Ty-1 ATGTGATTTCTAAATAAGTAAACAATTGACTTAACTATATTAGATTTCTTAG-CAAAAAA

R1.Col-0 ATATGATTACTAAATAAGTAAACAATTGACTTGCTTATATTAGATTTCTTAGCAAAAAAA

R2.Col-0 ATGTGATTTCTAAATAAGTAAACAATTGACTTAAATATATTAGATTTCTTAG-CAAGAAA

R3.Col-0 ATGTGATTTCTAAATAAGTAAACAATTGACTTAACTATATTAGATTTCTTAG-CAAAAAA

R4.Col-0 ATGTGATTTCTAAATAAGTAAACAATTGACTTAACTATATTAGATTTCTTAG-CAAAAAA

R1.Fly-2-2 ATATGATTACTAAATAAGTAAACAATTGACTTGCTTATATTAGATTTCTTAGCAAAAAAA

R2.Fly-2-2 ATGTGATTTCTAAATAAGTAAACAATTGACTTAACTATATTAGATTTCTTAG-CAAAAAA

R3.Fly-2-2 ATGTGATTTCTAAATAAGTAAACAATTGACTTAACTATATTAGATTTCTTAG--CAAAAA

R4.Fly-2-2 ATGTGATTTCTAAATAAGTAAACAATTGACTTAACTATATTAGATTTCTTAG-CAAAAAA

R1.Tur4 ATATGATTACTAAATAAGTAAACAATTGACTTGCTTATATTAGATTTCTTAGCAAAAAAA

R2.Tur4 ATGTGATTTCTAAATAAGTAAACAATTGACTTAACTATATTAGATTTCTTAG-CAAAAAA

R3.Tur4 ATGTGATTTCTAAATAAGTAAACAATTGACTTAACTATATTAGATTTCTTAG--CAAAAA

R4.Tur4 ATGTGATTTCTAAATAAGTAAACAATTGACTTAACTATATTAGATTTCTTAG-CAAAAAA

R1.Kyo -CAATTAATGAA---ATAAACAATTTATGATTTTGAACTTATTAAAGCAATAAAGGTTCA

R2.Kyo -CAATTTATGAA---ATAAATAGTTTATAATTTTGAACTTATTAAATCAATAAGGGTCCA

R3.Kyo -CAATTGACGAA---ATAAATAATTTACAATTT-GAACTTATTAAAGCAATAAGGGTCCA

R4.Kyo TAAAATAATTAATAGATAAATAATTTATGATTTTGAACTTATTAAAGCAATAAGGGTTCA

R1.Eri-1 -CAATTAATGAA---ATAAACAATTTATGATTTTGAACTTATTAAAGCAATAAAGGTCCA

R2.Eri-1 -CAATTTATGAA---ATAAATAGTTTATAATTTTGAACTTATTAAATCAATAAGGGTCCA

R3.Eri-1 -CAATTGACGAA---ATAAATAATTTACAATTT-GAACTTATTAAAGCAATAAGG-TCCA

R4.Eri-1 TAAAATAATTAATAGATAAATAATTTATGATTTTAAACTTATTAAAGCAATAAGGATTCA

R1.Ler -CAATTAATGAA---ATAAACAATTTATTATTTTGAACTTATTAAAGCAATAAAGGTCCA

R2.Ler -AAATTGATGAA---ATAAATAATTTATAATTTTAAACTTATTAAATCAATAAAGGTCCA

R3.Ler -CAATTTATGAA---ATAAATAGTTTATAATTTTGAACTTATTAAATCAATAAGGGTCTA

R4.Ler -CAATTGACGAA---ATAAATAATTTACAATTT-GAACTTATTAAAGCAATAAGGGTCCA

R5.Ler TAAAATAATTAATAGATAAATAATTTATGATTTTGAACTTATTAAAGTAATAAGGGTTCA

R1.C24 ACAATTAATGAA---ATAAACAATTTATGATTTTAAACTTATTAAAGCAATAAAGGTCCA

R2.C24 -AAATTGATGAA---ATAAATAATTTATAATTTTGAACTTATTAAATCAATAAAGGTCCA

R3.C24 -AAATTGATGAA---ATAAATAATTTACAATTTTGAACTTATTAAATCAATAAGGGTTCA

R4.C24 -CAATTTATGAA---ATAAATAGTTTATAATTTTGAACTTATTAAATCAATAAGGGTCCA

R5.C24 -CAATTGACGAA---ATAAATAATTTACAATTT-GAACTTATTAAAGCAATAAGGGTCCA

R6.C24 TAAAATAATTAATAGATAAATAATTTATGATTTTGAATTTATTAAAGCAATAAGGGTTCA

R1.Tos82-387 -CAATTAATGAA---ATAAACAATTTATGATTTTGAACTTATTAAAGCAATAAAGGTCCA

R2.Tos82-387 -AAATTGATGAA---ATAAATAATTTATAATTTTCAACTTATTAAATCAATAAAGGTCCA

R3.Tos82-387 -AAATTGATGAA---ATAAATAATTTACAATTTTGAACTTATTAAATCAATAAGGGTTCA

R4.Tos82-387 -CAATTTATGAA---ATAAATAGTTTATAATTTTGAACTTATTAAATCAATAAGGGTCCA

R5.Tos82-387 -CAATTGACGAA---ATAAATAATTTACAATTT-GAACTTATTAAAGCAATAAGGGTCCA

R6.Tos82-387 TAAAATAATTAATAGATAAATAATTTATGATTTTGAACTTATTAAAGCAATAAGGGTTCA

R1.An-1 -CAATTAATGAA---ATAAACAATTTATGATTTTGAAATTATTAAAGCAATAAAGGTCCA

R2.An-1 -AAATTGATGAA---ATAAATAATTTATAATTTTGAACTTATTAAATAAATAAAGGTCCA

R3.An-1 -AAATTGATGAA---ATAAATAATTTACAATTTTGAACTTATTAAATCAATAAGGGTCCA

R4.An-1 -CAATTTATGAA---ATAAATAGTTTATAATTTTGAACTTATTAAATCAATAAGGGTCCA

R5.An-1 -CAATTGACGAA---ATAAATAATTTACAATTT-GAACTTATTAAAGCAATAAGGGTCCA

R6.An-1 TAAAATAATTAATAGATAAATAATTTATGATTT-GAACTTATTAAAGCAATAAGGGTTCA

R1.KBS-Mac-74 -CAATTAATGAA---ATAAACAATTTATGATTTTGAAATTATTAAAGCAATAAAGGTCCA

R2.KBS-Mac-74 -AAATTGATGAA---ATAAATAATTTATAATTTTGAACTTATTAAATAAATAAAGGTCCA

R3.KBS-Mac-74 -AAATTGATGAA---ATAAATAATTTACAATTTTGAACTTATTAAATCAATAAGGGTCCA

R4.KBS-Mac-74 -CAATTTATGAA---ATAAATAGTTTATAATTTTGAACTTATTAAATCAATAAGGGTCCA

R5.KBS-Mac-74 -CAATTGACGAA---ATAAATAATTTACAATTT-GAACTTATTAAAGCAATAAGGGTCCA

R6.KBS-Mac-74 TAAAATAATTAATAGATAAATAATTTATGATTT-GAACTTATTAAAGCAATAAGGGTTCA

R1.Cdm-0 -CAATTAATGAA---ATAAACAATTTATTATTTTGAACTTATTAAAGCAATAAAGGTCCA

R2.Cdm-0 -AAATTGATGAA---ATAAATAATTTATAATTTTAAACTTATTAAATCAATAAAGGTCCA

R3.Cdm-0 -CAATTTATGAA---ATAAATAGTTTATAATTTTGAACTTATTAAATCAATAAGGGTCCA

R4.Cdm-0 -CAATTGACGAA---ATAAATAATTTACAATTT-GAACTTATTAAAGCAATAAGGGTCCA

R5.Cdm-0 TAAAATAATTAATAGATAAATAATTTATGATTTTGAACTTATTAAAGTAATAAGGGTTCA

R1.Kn-0 -CAATTAATGAA---ATAAACAATTTATGATTTTGAACTTATTAAAGCAATAAAGGTCCA

R2.Kn-0 -AAATTGATGAA---ATAAATAATTTATAATTTTAAACTTATTAAATCAATAAAGGTCCA

R3.Kn-0 -CAATTTATGAA---ATAAATAGTTTATAATTTTGAACTTATTAAATCAATAAGGGTCCA

R4.Kn-0 -CAATTGACGAA---ATAAATAATTTACAATTT-GAACTTATTAAAGCAATAAGGGTCCA

R5.Kn-0 TAAAATAATTAATAGATAAATAATTTATGATTTTGAACTTATTAAAGCAATAAGGGTTCA

R1.Cvi -CAATTAATGAA---ATAAACAATTTATGATTTTGAACTTATTAAAGCAATAAAGGTCCA

R2.Cvi -AAATTGATGAA---ATAAATAATTTATAATTTTGAACTTATTAAACCAATAAAGGTCCA

R3.Cvi -TAATTCATGAA---ATAAATAGTTTATAATTTTGAACTTATTAAATCAATAAGGGTCCA

R4.Cvi ------------------------------------------------------------

R5.Cvi TAAAATAATTAATAGATAAATAATTTATGATTTTGAACTTATTAAAGCAATAAGGGTTCA

R1.Fja-2-4 -CAATTAATGAA---ATAAACAATTTATGATTTTGAACTTATTAAAGCAATAAAGGTCCA

R2.Fja-2-4 -CAATTTATGAA---ATAAATAGTTTATAATTTTGAACTTATTAAATCAATAAGGGTCCA

R3.Fja-2-4 -CAATTGACGAA---ATAAATAATTTACAATTT-GAACTTATTAAAGCAATAAGGGTCCA

R4.Fja-2-4 TAAAATAATTAATAGATAAATAATTTATGATTTTGAACTTATTAAAGCAATAAGGGTTCA

R5.Fja-2-4 TAAAATAATTAATAGATAAATAATTTATGATTTTGAACTTATTAAAGCAATAAGGGTTTA

R1.Kor3 -CAATTAATGAA---ATAAACAATTTATGATTTTGAACTTATTAAAGCAATAAAGGTTCA

R2.Kor3 -CAATTTATGAA---ATAAATAGTTTATAATTTTGAACTTATTAAATCAATAAGGGTCCA

R3.Kor3 -CAATTGACGAA---ATAAATAATTTACAATTT-GAACTTATTAAAGCAATAAGGGTCCA

R4.Kor3 TAAAATAATTAATAGATAAATAATTTATGATTTTGAACTTATTAAAGCAATAAGGGTTCA

R1.Ty-1 -CAATTAATGAA---ATAAACAATTTATGATTTTGAACTTATTAAAGCAATAAAGGTCCA

R2.Ty-1 -AAATTGATGAA---ATAAATAATTTATAATTTTGAACTTATTAAATCAATAAAGGTCCA

R3.Ty-1 -AAATTGATGAA---ATAAATAATTTACAATTTTGAACTTATTAAATCAATAAGGGTCCA

R4.Ty-1 -TAATTCATGAA---ATAAATAGTTTATAATTTTGAACTTATTAAATCAATAAGGGTTCA

R1.Col-0 -CAATTAATGAA---ATAAACAATTTATGATTTTGAACTTATTAAAGCAATAAAGGTCCA

R2.Col-0 -AAATTGATGAA---ATAAATAATTTATAATTTTGAACTTATTAAATCAATAAAGGTCCA

R3.Col-0 -AAATTGATGAA---ATAAATAATTTACAATTTTGAACTTATTAAATCAATAAGGGTCCA

R4.Col-0 -TAATTCATGAA---ATACATAGTTTATAATTTTGAACTTATTAAATCAATAAGGGTTCA

R1.Fly-2-2 -CAATTAATGAA---ATAAACAATTTATGATTTTGAACTTATTAAAGCAATAAAGGTCCA

R2.Fly-2-2 -AAATTGATGAA---ATAAATAATTTATAATTTTAAACTTATTAAATCAATAAAGGTCCA

R3.Fly-2-2 -AAATTGATGAA---ATAAATAATTTACAATTTTGAACTTATTAAATCAATAAGGGTCCA

R4.Fly-2-2 -TAATTCATGAA---ATAAATAGTTTATAATTTTGAACTTATTAAATCAATAAGGGTCCA

R1.Tur4 -CAATTAATGAA---ATAAACAATTTATGATTTTGAACTTATTAAAGCAATAAAGGTCCA

R2.Tur4 -AAATTGATGAA---ATAAATAATTTATAATTTTAAACTTATTAAATCAATAAAGGTCCA

R3.Tur4 -AAATTGATGAA---ATAAATAATTTACAATTTTGAACTTATTAAATCAATAAGGGTCCA

R4.Tur4 -TAATTCATGAA---ATAAATAGTTTATAATTTTGAACTTATTAAATCAATAAGGGTCCA

R1.Kyo TAGCTCAGTGGTAGAGCAATTGACTGCAGATCAATAGGTCACCGGTTCAAACCCGATTGG

R2.Kyo TAGCTCAGTGGTAGAGCAATTGACTGCAGATCAATAGGTCACCGGTTCGAACCCGGTTGG

R3.Kyo TAGCTCAGTGGTAGAGCAATTGACTGCAGATCAATAGGTCACCGGTTCGAATCCGATTGG

R4.Kyo TAGCTCAGTGGTAGAGCAATTGACTGCAGATCAATAGGTCACCGGTTTGAACCCGGTTGG

R1.Eri-1 TAGCTCAGTGGTAGAGCAATTGACTGCAGATCAATAGGTCACCGGTTCAAACCCGATTGG

R2.Eri-1 TAGCTCAGTGGTAGAGCAATTGACTGCAGATCAATAGGTCACCGGTTCGAACCCGGTTGG

R3.Eri-1 TAGCTCAGTGGTAGAGCAATTGACTGCAGATCAATAGGTCACCGGTTCGAACCCGGTTGG

R4.Eri-1 TAGCTCAGTGGTAGAGCAATTGACTGCAGATCAATAGGTCACCGGTTCGAATCCGGTTGG

R1.Ler TAGCTCAGTGGTAGAGCAATTGACTGCAGATCAATAGGTCACCGGTTCAAACCCGATTGG

R2.Ler TAGCTCAGTGGTAGAGCAATTGACTGCAGAACAATAGGTCACAGGTTCGAACCCGGTTGG

R3.Ler TAGCTCAGTGGTAGAGCAATTGACTGCAGATCAATAGGTCACCGGTTCGAACCCGGTTGG

R4.Ler TAGCTCAGTGGTAGAGCAATTGACTGCAGATCAATAGGTCACCGGTTCGAACCCGGTTGG

R5.Ler TAACTCAGTGGTAGAGCAATTGACTGCAGATCAATAGGTCACCGGTTTGAACCCGGTTAG

R1.C24 TAGCTCAGTGGTAGAGCAATTGACTGCAGATCAATAGGTCACCGGTTCAAACCCGATTGG

R2.C24 TAGCTCAGTGGTAGAGCAATTGACTGCAGATCAATAGGTCACCGGTTCGAACCCGGTTGG

R3.C24 TAGCTCAGTGATAGAGCAATTGACTGCAGATCAATAGGTCACCGGTTCGAACCCGGTTGG

R4.C24 TAGCTCAGTGGTAGAGCAATTGACTGCAGATCAATAGGTCACCGGTTTGAACCCGGTTGG

R5.C24 TAGTTCAGTGGTAGAGCAATTGACTGCAGATCAATAGGTCACCGGTTCGAATCCGGTTGG

R6.C24 TAGCTCAGTGGTAGAGCAATTGACTGCAGATCAATAGGTCACCGGTTCGAACCCGGTTGG

R1.Tos82-387 TAGCTCAGTGGTAGAGCAATTGATTGCAGATCAATAGGTCACCGGTTCAAACCTGATTGG

R2.Tos82-387 TAGCTCAGTGGTAGAGCAATTGACTGCAGATCAATAGGTCACCGGTTCGAACCCGGTTAG

R3.Tos82-387 TAGCTCAGTGATAGAGCAATTGACTGCAGATCAATAGGTCACCGGTTCGAACCCGGTTGG

R4.Tos82-387 TAGCTCAGTGGTAGAGCAATTAACTGCAGATCAATAGGTCACCGGTTCGAACCCGGTTGG

R5.Tos82-387 TACCTCAGTGGTAGAGCAATTGACTGCAGATCAATAGGTCACCGGTTCGAACCCGGTTGG

R6.Tos82-387 TAGCTCAGTGGTAGAGCAATTGACTGCAGATCAATAGATCACCGGTTCGAACCCGGTTGG

R1.An-1 TAGCTCAGTGGTAGAGCAATTGACTGCAGATCAATAGGTCACCGGTTTGAACCCGATTGG

R2.An-1 TAGCTCAGTGGTAGAGCAATTGACTGCAGATCAATAGGTTACCGGTTCGAACCCGGTTGG

R3.An-1 TAGCTCAGTGATAGAGCAATTGACTGCAGATCAATAGGTCACCGGTTCGAACCCGGTTGG

R4.An-1 TAGCTCAGTGGTAGAGCAATTGACTGCAGATCAATAGGTCACCGGTTCGAACCCGGTTGG

R5.An-1 TAGCTCAGTGGTAGAGCAATTGGCTGCAGATCAATAGGTCACTGGTTCGAACCCGGTTGG

R6.An-1 TAGCTCAGTGGTAGAGCAATTGACTGCAGATCAATAGGTCACCGGTTCGAATCCGGTTGG

R1.KBS-Mac-74 TAGCTCAGTGGTAGAGCAATTGACTGCAGATCAATAGGTCACCGGTTTGAACCCGATTGG

R2.KBS-Mac-74 TAGCTCAGTGGTAGAGCAATTGACTGCAGACCAATAGGTTACCGGTTCGAACCCGGTTGG

R3.KBS-Mac-74 TAGCTCAGTGATAGAGCAATTGACTGCAGATCAATAGGTCACCGGTTCGAACCCGGTTGG

R4.KBS-Mac-74 TAGCTCAGTGGTAGAGCAATTGACTGCAGATCAATAGGTCACCGGTTCGAACCCGGTTGG

R5.KBS-Mac-74 TAGCTCAGTGGTAGAGCAATTGGCTGCAGATCAATAGGTCACTGGTTCGAACCCGGTTGG

R6.KBS-Mac-74 TAGCTCAGTGGTAGAGCAATTGACTGCAGATCAATAGGTCACCGGTTCGAATCCGGTTGG

R1.Cdm-0 TAGCTCAGTGGTAGAGCAATTGACTGCAGATCAATAGGTCACCGGTTCAAACCCGATTGG

R2.Cdm-0 TAGCTCAGTGGTAGAGCAATTGACTGCAGAACAATAGGTCACAGGTTCGAACCCGGTTGG

R3.Cdm-0 TAGCTCAGTGGTAGAGCAATTGACTGCAGATCAATAGGTCACCGGTTCGAACCCGGTTGG

R4.Cdm-0 TAGCTCAGTGGTAGAGCAATTGACTGCAGATCAATAGGTCACCGGTTCGAACCCGGTTGG

R5.Cdm-0 TAACTCAGTGGTAGAGCAATTGACTGCAGATCAATAGGTCACCGGTTTGAACCCGGTTGG

R1.Kn-0 TAGCTCAGTGGTAGAGCAATTGACTGCAGATCAATAGGTCACCGGTTCAAACCCGATTGG

R2.Kn-0 TAGCTCAGTGGTAGAGCAATTGACTGCAGAACAATAGGTCACAGGTTCGAACCCGGTTGG

R3.Kn-0 TAGCTCAGTGGTAGAGCAATTGACTGCAGATCAATAGGTCACCGGTTCGAACCCGGTTGG

R4.Kn-0 TAGCTCAGTGGTAGAGCAATTGACTGCAGATCAATAGGTCACCGGTTCGAATCCGGTTGG

R5.Kn-0 TAGCTCAGTGGTAGAGCAATTGACTGCAGATCAATAGGTCACCGGTTTGAACCCGGTTGG

R1.Cvi TATCTCAGTGGTAGAGCAATTGACTGCAAATCAATAGGTCACCGGTTCGAACCCGATTGG

R2.Cvi TAGCACAGTGGTAGAGCAATTGATTGCAGATCAATAGGTCACCGGTTTGAACCCGGTTGG

R3.Cvi TAGCTCAGTGGTAGAGCAATTGACTGCAGATCAATAGGTCACCGGTTCGAACCCGGTTGG

R4.Cvi ------------------------------------------------------------

R5.Cvi TAGCTCAGTGGTAGAGCAATTGACTGCAGATCAATAGGTCACCGGTTCGAACCCGGTTGG

R1.Fja-2-4 TAGCTCAGTGGTAGAGCAATTGACTGCAGATCAATAGGTCACCGGTTCAAATCCGATTGG

R2.Fja-2-4 TAGCTCAGTGGTAGAGCAATTGACTGCAGATCAATAGGTCACCGGTTCGAACCCGGTTGG

R3.Fja-2-4 TAGCTCAGTGGTAGAGCAATTGACTGCAGATCAATAGGTCACCGGTTCGAATCCGATTGG

R4.Fja-2-4 TAGCTCAGTGGTAGAGCAATTGACTGCAGATCAATAGGTCACCGGTTTGAACCCGGTTGG

R5.Fja-2-4 TAGCTCAGTGGTAGAGCAATTGACTGCAGATCAATAGGTCACCGGTTTGAACCCGGTTGG

R1.Kor3 TAGCTCAGTGGTAGAGCAATTGACTGCAGATCAATAGGTCACCGGTTCAAACCCGATTGG

R2.Kor3 TAGCTCAGTGGTAGAGCAATTGACTGCAGATCAATAGGTCACCGGTTCGAACCCGGTTGG

R3.Kor3 TAGCTCAGTGGTAGAGCAATTGACTGCAGATCAATAGGTCACCGGTTCGAATCCGATTGG

R4.Kor3 TAGCTCAGTGGTAGAGCAATTGACTGCAGATCAATAGGTCACCGGTTTGAACCCGGTTGG

R1.Ty-1 TAGCTCAGTGGTAGAGCAATTGACTGCAGATCAATAGGTCACCGGTTCGAACCCGATTGG

R2.Ty-1 TAGCTCAGTGGTAGAGCAATTGACTGCAGATCAATAGGTCACCGGTTTGAACCTGGTTGG

R3.Ty-1 TAGCTCAGTGATAGAGCAATTGACTGCAGATCAATAGGTCACCGGTTCGAACCCGGTTGG

R4.Ty-1 TAGCTCAGTAGTAGAGCAATTGACTGCAGATCAATAGGTCACCGGTTCGAACCCGGTTGG

R1.Col-0 TAGCTCAGTGGTAGAGCAATTGACTGCAGATCAATAGGTCACCGGTTCGAACCCGATTGG

R2.Col-0 TAGCTCAGTGGTAGAGCAATTGACTGCAGATCAATAGGTCACCGGTTTGAACCTGGTTGG

R3.Col-0 TAGCTCAGTGATAGAGCAATTGACTGCAGATCAATAGGTCACCGGTTCGAACCCGGTTGG

R4.Col-0 TAGCTCAGTGGTAGAGCAATTGACTGCAGATCAATAGGTCACCGGTTCGAACCCGGTTGG

R1.Fly-2-2 TAGCTCAGTGGTAGAGCAATTGACTGCAGATCAATAGGTCACCGGTTCAAACCCGATTGG

R2.Fly-2-2 TAGCTCAGTGGTAGAGCAATTGACTGCAGATCAATAGGTCACCGGTTCAAACCCGGTTGG

R3.Fly-2-2 TAGCTCAGTGATAGAGCAATTGACTGCAGATCAATAGGTCACCGGTTCGAACCCGGTTGG

R4.Fly-2-2 TAGCTCAGTGGTAGAGCAATTGACTGCAGATCAATAGGTCACCGGTTCGAACCCGGTTGG

R1.Tur4 TAGCTCAGTGGTAGAGCAATTGACTGCAGATCAATAGGTCACCGGTTCAAACCCGATTGG

R2.Tur4 TAGCTCAGTGGTAGAGCAATTGACTGCAGATCAATAGGTCACCGGTTCAAACCCGGTTGG

R3.Tur4 TAGCTCAGTGATAGAGCAATTGACTGCAGATCAATAGGTCACCGGTTCGAACCCGGTTGG

R4.Tur4 TAGCTCAGTGGTAGAGCAATTGACTGCAGATCAATAGGTCACCGGTTCGAACCCGGTTGG

R1.Kyo GCCCT

R2.Kyo GCCCT

R3.Kyo GCCCT

R4.Kyo GCCCT

R1.Eri-1 GCCCT

R2.Eri-1 GCCCT

R3.Eri-1 GCCCT

R4.Eri-1 GCCCT

R1.Ler GCCCT

R2.Ler GCCCT

R3.Ler GCCCT

R4.Ler GCCCT

R5.Ler GCCCT

R1.C24 GCCCT

R2.C24 GCCCT

R3.C24 GCCCT

R4.C24 GCCCT

R5.C24 GCCTT

R6.C24 GCCCT

R1.Tos82-387 GCCCT

R2.Tos82-387 GCCCT

R3.Tos82-387 GCCCT

R4.Tos82-387 GCCCT

R5.Tos82-387 GCCCT

R6.Tos82-387 ATCCT

R1.An-1 GCCCT

R2.An-1 GCCCT

R3.An-1 GCCCT

R4.An-1 GCCCT

R5.An-1 GCCCT

R6.An-1 GCCCT

R1.KBS-Mac-74 GCCCT

R2.KBS-Mac-74 GCCCT

R3.KBS-Mac-74 GCCCT

R4.KBS-Mac-74 GCCCT

R5.KBS-Mac-74 GCCCT

R6.KBS-Mac-74 GCCCT

R1.Cdm-0 GCCCT

R2.Cdm-0 GCCCT

R3.Cdm-0 GCCCT

R4.Cdm-0 GCCCT

R5.Cdm-0 GCCCT

R1.Kn-0 GCCCT

R2.Kn-0 GCCCT

R3.Kn-0 GCCCT

R4.Kn-0 GCCCT

R5.Kn-0 GCCCT

R1.Cvi GCCCT

R2.Cvi GCCCT

R3.Cvi GCCCT

R4.Cvi -----

R5.Cvi GCTCT

R1.Fja-2-4 GCCCT

R2.Fja-2-4 GCCCT

R3.Fja-2-4 GCCCT

R4.Fja-2-4 GCCCT

R5.Fja-2-4 GCCCT

R1.Kor3 GCCCT

R2.Kor3 GCCCT

R3.Kor3 GCCCT

R4.Kor3 GCCCT

R1.Ty-1 GCCCT

R2.Ty-1 GCCCT

R3.Ty-1 GCCCT

R4.Ty-1 GCCCT

R1.Col-0 GCCCT

R2.Col-0 GCCCT

R3.Col-0 GCCCT

R4.Col-0 GCCCT

R1.Fly-2-2 GCCCT

R2.Fly-2-2 GCCCT

R3.Fly-2-2 GCCCT

R4.Fly-2-2 GCCCT

R1.Tur4 GCCCT

R2.Tur4 GCCCT

R3.Tur4 GCCCT

R4.Tur4 GCCCT

**Supplementary Figure S10.** Multiple sequence alignment of chromosome 5 cluster tRNA sequences used to construct the tree in Supplementary Figure S2.

R1.Kyo AGGTTCATAGCTCAGTGGTAGAGCAATTGACTGCAGATCAATAGGTCACCGGTTCAAACCCGATTGGGCCCT

R1.Kor3 AGGTTCATAGCTCAGTGGTAGAGCAATTGACTGCAGATCAATAGGTCACCGGTTCAAACCCGATTGGGCCCT

R1.Eri-1 AGGTCCATAGCTCAGTGGTAGAGCAATTGACTGCAGATCAATAGGTCACCGGTTCAAACCCGATTGGGCCCT

R1.Ler AGGTCCATAGCTCAGTGGTAGAGCAATTGACTGCAGATCAATAGGTCACCGGTTCAAACCCGATTGGGCCCT

R1.C24 AGGTCCATAGCTCAGTGGTAGAGCAATTGACTGCAGATCAATAGGTCACCGGTTCAAACCCGATTGGGCCCT

R1.Cdm-0 AGGTCCATAGCTCAGTGGTAGAGCAATTGACTGCAGATCAATAGGTCACCGGTTCAAACCCGATTGGGCCCT

R1.Kn-0 AGGTCCATAGCTCAGTGGTAGAGCAATTGACTGCAGATCAATAGGTCACCGGTTCAAACCCGATTGGGCCCT

R1.Fly-2-2 AGGTCCATAGCTCAGTGGTAGAGCAATTGACTGCAGATCAATAGGTCACCGGTTCAAACCCGATTGGGCCCT

R1.Tur4 AGGTCCATAGCTCAGTGGTAGAGCAATTGACTGCAGATCAATAGGTCACCGGTTCAAACCCGATTGGGCCCT

R1.Fja-2-4 AGGTCCATAGCTCAGTGGTAGAGCAATTGACTGCAGATCAATAGGTCACCGGTTCAAATCCGATTGGGCCCT

R2.Fly-2-2 AGGTCCATAGCTCAGTGGTAGAGCAATTGACTGCAGATCAATAGGTCACCGGTTCAAACCCGGTTGGGCCCT

R2.Tur4 AGGTCCATAGCTCAGTGGTAGAGCAATTGACTGCAGATCAATAGGTCACCGGTTCAAACCCGGTTGGGCCCT

R1.An-1 AGGTCCATAGCTCAGTGGTAGAGCAATTGACTGCAGATCAATAGGTCACCGGTTTGAACCCGATTGGGCCCT

R1.KBS-Mac-74 AGGTCCATAGCTCAGTGGTAGAGCAATTGACTGCAGATCAATAGGTCACCGGTTTGAACCCGATTGGGCCCT

R1.Ty-1 AGGTCCATAGCTCAGTGGTAGAGCAATTGACTGCAGATCAATAGGTCACCGGTTCGAACCCGATTGGGCCCT

R1.Col-0 AGGTCCATAGCTCAGTGGTAGAGCAATTGACTGCAGATCAATAGGTCACCGGTTCGAACCCGATTGGGCCCT

R2.Kyo GGGTCCATAGCTCAGTGGTAGAGCAATTGACTGCAGATCAATAGGTCACCGGTTCGAACCCGGTTGGGCCCT

R2.Eri-1 GGGTCCATAGCTCAGTGGTAGAGCAATTGACTGCAGATCAATAGGTCACCGGTTCGAACCCGGTTGGGCCCT

R4.Ler GGGTCCATAGCTCAGTGGTAGAGCAATTGACTGCAGATCAATAGGTCACCGGTTCGAACCCGGTTGGGCCCT

R4.An-1 GGGTCCATAGCTCAGTGGTAGAGCAATTGACTGCAGATCAATAGGTCACCGGTTCGAACCCGGTTGGGCCCT

R4.KBS-Mac-74 GGGTCCATAGCTCAGTGGTAGAGCAATTGACTGCAGATCAATAGGTCACCGGTTCGAACCCGGTTGGGCCCT

R3.Cdm-0 GGGTCCATAGCTCAGTGGTAGAGCAATTGACTGCAGATCAATAGGTCACCGGTTCGAACCCGGTTGGGCCCT

R4.Cdm-0 GGGTCCATAGCTCAGTGGTAGAGCAATTGACTGCAGATCAATAGGTCACCGGTTCGAACCCGGTTGGGCCCT

R3.Kn-0 GGGTCCATAGCTCAGTGGTAGAGCAATTGACTGCAGATCAATAGGTCACCGGTTCGAACCCGGTTGGGCCCT

R3.Cvi GGGTCCATAGCTCAGTGGTAGAGCAATTGACTGCAGATCAATAGGTCACCGGTTCGAACCCGGTTGGGCCCT

R2.Fja-2-4 GGGTCCATAGCTCAGTGGTAGAGCAATTGACTGCAGATCAATAGGTCACCGGTTCGAACCCGGTTGGGCCCT

R2.Kor3 GGGTCCATAGCTCAGTGGTAGAGCAATTGACTGCAGATCAATAGGTCACCGGTTCGAACCCGGTTGGGCCCT

R4.Fly-2-2 GGGTCCATAGCTCAGTGGTAGAGCAATTGACTGCAGATCAATAGGTCACCGGTTCGAACCCGGTTGGGCCCT

R4.Tur4 GGGTCCATAGCTCAGTGGTAGAGCAATTGACTGCAGATCAATAGGTCACCGGTTCGAACCCGGTTGGGCCCT

R3.Eri-1 AGGTCCATAGCTCAGTGGTAGAGCAATTGACTGCAGATCAATAGGTCACCGGTTCGAACCCGGTTGGGCCCT

R2.C24 AGGTCCATAGCTCAGTGGTAGAGCAATTGACTGCAGATCAATAGGTCACCGGTTCGAACCCGGTTGGGCCCT

R3.Ler GGGTCTATAGCTCAGTGGTAGAGCAATTGACTGCAGATCAATAGGTCACCGGTTCGAACCCGGTTGGGCCCT

R4.Tos82-387 GGGTCCATAGCTCAGTGGTAGAGCAATTAACTGCAGATCAATAGGTCACCGGTTCGAACCCGGTTGGGCCCT

R3.C24 GGGTTCATAGCTCAGTGATAGAGCAATTGACTGCAGATCAATAGGTCACCGGTTCGAACCCGGTTGGGCCCT

R3.Tos82-387 GGGTTCATAGCTCAGTGATAGAGCAATTGACTGCAGATCAATAGGTCACCGGTTCGAACCCGGTTGGGCCCT

R6.C24 GGGTTCATAGCTCAGTGGTAGAGCAATTGACTGCAGATCAATAGGTCACCGGTTCGAACCCGGTTGGGCCCT

R4.Col-0 GGGTTCATAGCTCAGTGGTAGAGCAATTGACTGCAGATCAATAGGTCACCGGTTCGAACCCGGTTGGGCCCT

R3.An-1 GGGTCCATAGCTCAGTGATAGAGCAATTGACTGCAGATCAATAGGTCACCGGTTCGAACCCGGTTGGGCCCT

R3.KBS-Mac-74 GGGTCCATAGCTCAGTGATAGAGCAATTGACTGCAGATCAATAGGTCACCGGTTCGAACCCGGTTGGGCCCT

R3.Ty-1 GGGTCCATAGCTCAGTGATAGAGCAATTGACTGCAGATCAATAGGTCACCGGTTCGAACCCGGTTGGGCCCT

R3.Col-0 GGGTCCATAGCTCAGTGATAGAGCAATTGACTGCAGATCAATAGGTCACCGGTTCGAACCCGGTTGGGCCCT

R3.Fly-2-2 GGGTCCATAGCTCAGTGATAGAGCAATTGACTGCAGATCAATAGGTCACCGGTTCGAACCCGGTTGGGCCCT

R3.Tur4 GGGTCCATAGCTCAGTGATAGAGCAATTGACTGCAGATCAATAGGTCACCGGTTCGAACCCGGTTGGGCCCT

R5.Cvi GGGTTCATAGCTCAGTGGTAGAGCAATTGACTGCAGATCAATAGGTCACCGGTTCGAACCCGGTTGGGCTCT

R4.Ty-1 GGGTTCATAGCTCAGTAGTAGAGCAATTGACTGCAGATCAATAGGTCACCGGTTCGAACCCGGTTGGGCCCT

R3.Kyo GGGTCCATAGCTCAGTGGTAGAGCAATTGACTGCAGATCAATAGGTCACCGGTTCGAATCCGATTGGGCCCT

R3.Fja-2-4 GGGTCCATAGCTCAGTGGTAGAGCAATTGACTGCAGATCAATAGGTCACCGGTTCGAATCCGATTGGGCCCT

R3.Kor3 GGGTCCATAGCTCAGTGGTAGAGCAATTGACTGCAGATCAATAGGTCACCGGTTCGAATCCGATTGGGCCCT

R4.Kn-0 GGGTCCATAGCTCAGTGGTAGAGCAATTGACTGCAGATCAATAGGTCACCGGTTCGAATCCGGTTGGGCCCT

R4.Eri-1 GGATTCATAGCTCAGTGGTAGAGCAATTGACTGCAGATCAATAGGTCACCGGTTCGAATCCGGTTGGGCCCT

R6.An-1 GGGTTCATAGCTCAGTGGTAGAGCAATTGACTGCAGATCAATAGGTCACCGGTTCGAATCCGGTTGGGCCCT

R6.KBS-Mac-74 GGGTTCATAGCTCAGTGGTAGAGCAATTGACTGCAGATCAATAGGTCACCGGTTCGAATCCGGTTGGGCCCT

R4.Kyo GGGTTCATAGCTCAGTGGTAGAGCAATTGACTGCAGATCAATAGGTCACCGGTTTGAACCCGGTTGGGCCCT

R5.Kn-0 GGGTTCATAGCTCAGTGGTAGAGCAATTGACTGCAGATCAATAGGTCACCGGTTTGAACCCGGTTGGGCCCT

R4.Fja-2-4 GGGTTCATAGCTCAGTGGTAGAGCAATTGACTGCAGATCAATAGGTCACCGGTTTGAACCCGGTTGGGCCCT

R4.Kor3 GGGTTCATAGCTCAGTGGTAGAGCAATTGACTGCAGATCAATAGGTCACCGGTTTGAACCCGGTTGGGCCCT

R4.C24 GGGTCCATAGCTCAGTGGTAGAGCAATTGACTGCAGATCAATAGGTCACCGGTTTGAACCCGGTTGGGCCCT

R5.Fja-2-4 GGGTTTATAGCTCAGTGGTAGAGCAATTGACTGCAGATCAATAGGTCACCGGTTTGAACCCGGTTGGGCCCT

R5.Ler GGGTTCATAACTCAGTGGTAGAGCAATTGACTGCAGATCAATAGGTCACCGGTTTGAACCCGGTTAGGCCCT

R5.Cdm-0 GGGTTCATAACTCAGTGGTAGAGCAATTGACTGCAGATCAATAGGTCACCGGTTTGAACCCGGTTGGGCCCT

R2.Tos82-387 AGGTCCATAGCTCAGTGGTAGAGCAATTGACTGCAGATCAATAGGTCACCGGTTCGAACCCGGTTAGGCCCT

R2.An-1 AGGTCCATAGCTCAGTGGTAGAGCAATTGACTGCAGATCAATAGGTTACCGGTTCGAACCCGGTTGGGCCCT

R2.KBS-Mac-74 AGGTCCATAGCTCAGTGGTAGAGCAATTGACTGCAGACCAATAGGTTACCGGTTCGAACCCGGTTGGGCCCT

R2.Ty-1 AGGTCCATAGCTCAGTGGTAGAGCAATTGACTGCAGATCAATAGGTCACCGGTTTGAACCTGGTTGGGCCCT

R2.Col-0 AGGTCCATAGCTCAGTGGTAGAGCAATTGACTGCAGATCAATAGGTCACCGGTTTGAACCTGGTTGGGCCCT

R1.Tos82-387 AGGTCCATAGCTCAGTGGTAGAGCAATTGATTGCAGATCAATAGGTCACCGGTTCAAACCTGATTGGGCCCT

R5.An-1 GGGTCCATAGCTCAGTGGTAGAGCAATTGGCTGCAGATCAATAGGTCACTGGTTCGAACCCGGTTGGGCCCT

R5.KBS-Mac-74 GGGTCCATAGCTCAGTGGTAGAGCAATTGGCTGCAGATCAATAGGTCACTGGTTCGAACCCGGTTGGGCCCT

R5.Tos82-387 GGGTCCATACCTCAGTGGTAGAGCAATTGACTGCAGATCAATAGGTCACCGGTTCGAACCCGGTTGGGCCCT

R5.C24 GGGTCCATAGTTCAGTGGTAGAGCAATTGACTGCAGATCAATAGGTCACCGGTTCGAATCCGGTTGGGCCTT

R1.Cvi AGGTCCATATCTCAGTGGTAGAGCAATTGACTGCAAATCAATAGGTCACCGGTTCGAACCCGATTGGGCCCT

R6.Tos82-387 GGGTTCATAGCTCAGTGGTAGAGCAATTGACTGCAGATCAATAGATCACCGGTTCGAACCCGGTTGGATCCT

R2.Cvi AGGTCCATAGCACAGTGGTAGAGCAATTGATTGCAGATCAATAGGTCACCGGTTTGAACCCGGTTGGGCCCT

R2.Ler AGGTCCATAGCTCAGTGGTAGAGCAATTGACTGCAGAACAATAGGTCACAGGTTCGAACCCGGTTGGGCCCT

R2.Cdm-0 AGGTCCATAGCTCAGTGGTAGAGCAATTGACTGCAGAACAATAGGTCACAGGTTCGAACCCGGTTGGGCCCT

R2.Kn-0 AGGTCCATAGCTCAGTGGTAGAGCAATTGACTGCAGAACAATAGGTCACAGGTTCGAACCCGGTTGGGCCCT
